# Manipulation of insulin signaling phenocopies evolution of a host-associated polyphenism

**DOI:** 10.1038/s41467-018-04102-1

**Published:** 2018-04-27

**Authors:** Meghan M. Fawcett, Mary C. Parks, Alice E. Tibbetts, Jane S. Swart, Elizabeth M. Richards, Juan Camilo Vanegas, Meredith Cenzer, Laura Crowley, William R. Simmons, Wenzhen Stacey Hou, David R. Angelini

**Affiliations:** 1 Department of Biology, Colby College, 5734 Mayflower Hill, Waterville, ME 04901 USA; 2 Department of Entomology, University of California, Davis, One Shields Avenue, Davis, CA 95616 USA; 3 Department of Genetics and Development, Columbia University Medical Center, 1130 Street Nicholas Avenue, Room 208B, New York, NY 10032 USA; 4 National Human Genome Research Institute, 49 Convent Drive, Bethesda, MD 20892 USA

## Abstract

Plasticity, the capacity of an organism to respond to its environment, is thought to evolve through changes in development altering the integration of environmental cues. In polyphenism, a discontinuous plastic response produces two or more phenotypic morphs. Here we describe evolutionary change in wing polyphenism and its underlying developmental regulation in natural populations of the red-shouldered soapberry bug, *Jadera haematoloma* (Insecta: Hemiptera: Rhopalidae) that have adapted to a novel host plant. We find differences in the fecundity of morphs in both sexes and in adult expression of insulin signaling components in the gonads. Further, the plastic response of ancestral-state bugs can be shifted to resemble the reaction norm of derived bugs by the introduction of exogenous insulin or RNA interference targeting the insulin signaling component encoded by *FoxO*. These results suggest that insulin signaling may be one pathway involved in the evolution of this polyphenism, allowing adaptation to a novel nutritional environment.

## Abstract (web summary)

The red-shouldered soapberry bug, *Jadera haematoloma*, is a potential model system for developmental plasticity. Here, the authors show that the reaction norm for wing polyphenism has evolved in a recently derived ecotype and identify insulin signaling as a candidate pathway underlying this adaptive change.

## Introduction

Phenotypic plasticity is a non-genetic source of biological variation^1–3^. Nevertheless, it arises from developmental systems, which are produced by gene products and subject to evolution^2–6^. The average response of individuals in a population to a given environmental influence describes their reaction norm^7,8^. Despite identification of regulatory mechanisms for some reaction norms^9–16^ and documentation of the evolution of plasticity in many systems^17–26^, mechanisms underlying reaction norm evolution in wild populations remain poorly understood. It remains unclear how genetic pathways that enable phenotypic sensitivity to the environment might change with evolution to modify a plastic response.

Wing polyphenism occurs in many insects, including in many true bugs (Hemiptera). In these species, adults develop with shortened wings and non-functional flight muscles. In most species, wing morphs are determined by environmental conditions, such as nutrition, or by a combination of genetic and environmental influences on development^5,11,27^. Although several developmental genetic pathways are associated with body size and organ growth regulation^28^, less is known about growth regulation in the context of polyphenism^29,30^. Recent studies of wing polyphenism in the brown planthopper *Nilaparvata lugens*^31^ and other species^32–34^ have identified a role for insulin signaling in the specification of wing morphs in these species.

The soapberry bug *Jadera haematoloma* (Hemiptera: Rhopalidae) is a promising model for integrative study into the regulation and evolution of a reaction norm. This insect exhibits a nutritionally regulated wing polyphenism, which has diverged among populations only in recent decades. Soapberry bugs are native to coastal dry hammock forest, along the Caribbean Sea from the US Gulf of Mexico to South America (Supplementary Fig. 4). *Jadera* feed on several plants of the soapberry family, native to the United States, including *Cardiospermum* sp. in Florida^35^. Since about 1950, a population of *J. haematoloma* has adapted to a novel host plant, the introduced goldenrain tree (*Koelreuteria* sp.)^36–38^. Therefore, traits associated with the introduced host are likely to be recently derived. *Cardiospermum* are perennial vines with relatively low but consistent seed production. In contrast, *Koelreuteria* are large trees that produce abundant seeds during a 3- to 4-month period. Compared with *Cardiospermum*, *Koelreuteria* seeds have a higher proportion of lipids and lower concentration of protein^38^. Many adaptations in morphology and life history have been documented for host-associated ecotypes of *J. haematoloma*^36–39^. The *Koelreuteria*-adapted ecotype is now widely distributed and expanding in the temperate United States, whereas populations resembling the ancestral state remain associated with native *Cardiospermum* vines in the Florida Keys (but see ref. ^40^).

Adult *J. haematoloma* of both sexes exhibit distinct wing morphs. Long-wing morphs have complete wings and functional flight muscles, whereas short-winged individuals are brachypterous, lack flight muscles, and are incapable of flight. The wings of each morph differ in their overall size, shape, and venation, and this variation is determined by environmental influences rather than genetic factors. Insect polyphenisms are typically mediated by one or more factors, including juvenile nutrition or density^5,27^. Previous work has suggested that *J. haematoloma* morphs are determined by juvenile nutrition, with a positive correlation between food level and frequency of the short-winged morph^41,42^. Host plants with different phenology present soapberry bugs with distinct nutritional environments^38^, potentially selecting for differences in wing morph frequencies or reaction norms.

Here we present evidence that *J. haematoloma* wing polyphenism is adaptive in females, but maladaptive in males of the native host ecotype. This sexual conflict is partially resolved in the derived *Koelreuteria* ecotype, in which the reaction norm offset has evolved to make short-winged individuals more common. The frequency of morphs is determined nutritionally and associated with differences in the expression of genes encoding insulin signaling components. Manipulation of the insulin signaling pathway alters this reaction norm, phenocopying its evolution in natural populations.

## Results

### Wing morphology

**Fig. 1:**
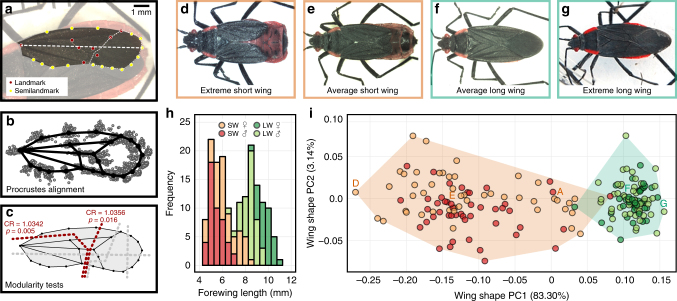
Two morphs of *J. haematoloma* differ most noticeably in the length and shape of the forewings. **a** Landmarks were defined at 24 locations across the wings, including 8 anatomical landmarks and 16 sliding semilandmarks. The dotted white line indicates the measurement of wing length. **b** Generalized Procrustes alignment of wings from 189 specimens. **c** Wings exhibit proximal and distal modules. Significant modular boundaries are marked by red dotted lines. Gray dotted lines demarcate alternative hypotheses that did not display significant modularity. **d**–**g** Extreme and average representatives of each wing morph, as determined from the first PC axis in **i**. **h** The distribution of wing length frequencies in 168 *J. haematoloma* adults is distinctly bimodal. Females are larger on average than males, but both sexes display polyphenic variation. Colors indicate sex and morph of sampled individuals. **i** Plot of 189 Procrustes-aligned specimens from **b** in tangent space, oriented to the first two principal component axes. The percentage of wing-shape variation along each axis appears in parentheses. Convex hulls highlight the disparity of each morph. Wing shape varies significantly by morph. Males and females do not differ significantly in wing shape. The individuals shown in **a** and **d**–**g** are labeled above their corresponding positions in morphospace

The range of wing phenotypes in *J. haematoloma* was characterized using linear and geometric morphometrics (Fig. 1 and Supplementary Figs. 1-3)^43^. Each sex has a bimodal distribution of wing lengths (Fig. 1h). Wing morphs are significantly different in wing length (permutation analysis of variance (ANOVA) with morph and sex as factors, *p* < 2.2 × 10^−16^). Wing morphs also differ dramatically in their static allometry^1^. Compared with head width (a proxy for body size), wing lengths of the two morphs have significantly different scaling relationships (Supplementary Fig. 1; analysis of covariance test for homogeneity of slopes *F*_1,162_ = 835.3, *p* = 7.96 × 10^−66^). Host-associated ecotype was not a significant factor in this relationship. Despite distinct differences in adult wing length among morphs, the size of juveniles or their external wing pads does not indicate an individual’s future morph (Supplementary Fig. 3).

Morphs differ significantly in wing shape as well as wing size. Variation in wing shape was characterized using landmark-based geometric morphometric analysis (Fig. 1a, b)^43–45^. Morphs differ significantly in their shape (Fig. 1i; permutation-based Procrustes ANOVA^44^ with morph and sex as factors, *F*_1,183_ = 335.1, *p* < 10^−4^). The wings of each morph differ most obviously in the shape of the distal wing region known as the membrane. Therefore, we reasoned that morph-specific cues might vary in their influence over different regions of the wing, highlighting anatomical and developmental modules. Several modularity hypotheses were tested by dividing the wing along proximal–distal and anterior–posterior axes, and evaluating the covariance ratio coefficient produced by comparisons within and between the proposed modules. As expected, the membrane and more proximal regions of the wing displayed significant modularity (covariance ratio test^45^, *p* < 0.05; Supplementary Note 1), whereas other groupings of wing landmarks did not (Fig. 1c).

Subjectively, short-wing morphs display a wide range of wing appearances, whereas long-wing morphs have more consistent appearance. Procrustes variance^44^ was significantly greater in short-wing morphs (difference in Procrustes variance = 0.00867; randomized residual permutation, *p* < 10^−6^). We also compared the wing shape disparity of each morph between ecotypes. Short-wing morphs differed slightly in their disparity between ecotypes (difference = 3.784 × 10^−3^, *p* = 0.0419) with greater disparity in *Koelreuteria* short-wing shapes. Among long-wing morphs, the difference in wing shape disparity for ecotypes was much greater (difference = 1.093 × 10^−3^, *p* = 1.69 × 10^−3^), with lower variation among *Cardiospermum*-associated long-wing bugs.

### Wing morph frequencies vary in the wild

To explore potential differences in wing polyphenism, we surveyed the frequency of each morph at numerous sites across the US range of *J. haematoloma*, including populations living on native and introduced hosts (Supplementary Fig. 4). The frequency of wing morphs varied widely, with some sites having only long-wing (11 of 64) or short-wing bugs (3 of 64), whereas most sites had both morphs. The geographic location of sites was not a significant factor in explaining variation in morph frequencies. However, morph ratios differed significantly among the most heavily sampled host plants: *Cardiospermum corindum*, a native host, and two introduced hosts, *Koelreuteria elegans* and *Koelreuteria paniculata* (permutation ANOVA, *p* < 2.2 × 10^−16^). Short-wing morphs were significantly more common on *K. paniculata* compared with each of the other two host plants (permutation test with Bonferroni correction; vs. *C. corindum*, *p* = 1.33 × 10^−3^; vs. *K. elegans*, *p* = 8.12 × 10^−5^).

### Mendelian inheritance does not explain morph frequencies

To test whether *J. haematoloma* wing morphs are determined genetically, we made 32 controlled crosses with all morph combinations (Supplementary Table 2). For each cross, we tested whether the resulting adult F_1_ morph ratio matched any Mendelian ratio (0:1, 1:3, 1:1, 3:1, or 1:0 short wing to long wing) or the prediction of a recessive lethal short-wing allele (2:1) using Fisher’s exact test with Bonferroni correction. These results were then compared for compatibility with Mendelian dominance of the long-wing or short-wing phenotypes and with the possibility of a recessive lethal allele. No Mendelian model consistently fit the data (Supplementary Table 2), consistent with a past study^41^ and supporting the conclusion that wing morphs in *J. haematoloma* are not primarily based on genetic factors.

### Nutrition-dependent wing polyphenism varies among ecotypes

**Fig. 2:**
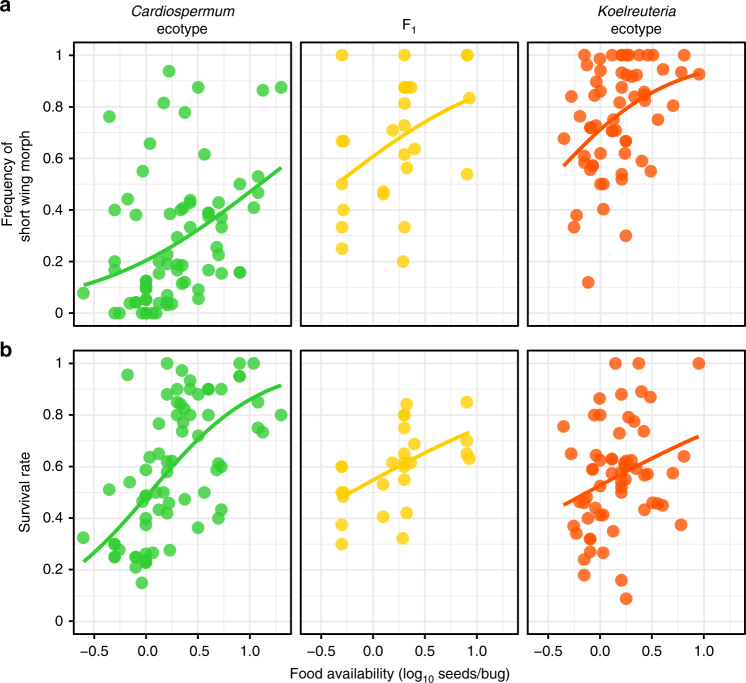
The frequency of wing morphs **a** and juvenile survival **b** correlates with juvenile food availability for two host-adapted ecotypes of *J. haematoloma*. The reaction norms vary among ecotypes. The ancestral *Cardiospermum* ecotype has a lower offset than the more recently adapted *Koelreuteria* ecotype. Each point represents a cohort raised under varying conditions of food availability. Survival rate also increases with food availability, but does not vary among ecotypes. The reaction norm of F_1_ ecotype hybrids strongly resembled the *Koelreuteria* ecotype. This dataset includes 3490 sampled adults from 133 treatments. Lines indicate logistic regression

As wing morph frequencies vary among wild populations on host plants with different phenology and resource availability (Supplementary Fig. 4), we wished to test whether ecotypes vary in their norms of reaction to food availability. Previous work has suggested that the *Cardiospermum* ecotype was more responsive to environmental conditions than soapberry bugs living on introduced *Koelreuteria*^41^. In field surveys, morph ratios are likely to be affected by environmental cues, genetic background, as well as selective forces that might bias the adults counted in field surveys. Therefore, we raised *J. haematoloma* cohorts in the lab under a wide range of food and conspecific density conditions, and surviving adults were scored by morph (Fig. 2a).

Using logistic regression, we compared models with predictive factors of seed number, the initial hatchling number in the cohort, and ecotype, alone, in combination, and allowing for interactions (Supplementary Table 3). We also considered null models predicting all short-wing or all long-wing morphs. This dataset included 3490 sampled adults from 133 independent treatments. An individual’s morph was best predicted by a model incorporating seed number, cohort size, and their interaction, as well as the bugs’ host ecotype. Each of these factors was a significant influence on the model fit (Supplementary Table 4 and Supplementary Fig. 5). Increasing seed number and lower conspecific competition (smaller cohorts) were correlated with increased frequencies of the short-wing morph (Fig. 2; 0.70% increased odds per seed; 0.95% decreased odds per bug). Compared with *Cardiospermum* ecotypes, populations of the *Koelreuteria* ecotype were 10–14 times more likely to be short winged (95% confidence on odds ratio). Models were tested in logistic regression for each ecotype alone, where seed number and cohort size were also found to be significant predictors of morph frequency (Supplementary Tables 5-8). These analyses reveal that ecotypes differ primarily in the offset of their reaction norms^46^, with *Koelreuteria* ecotypes having a lower nutritional threshold for short-wing development.

**Fig. 3:**
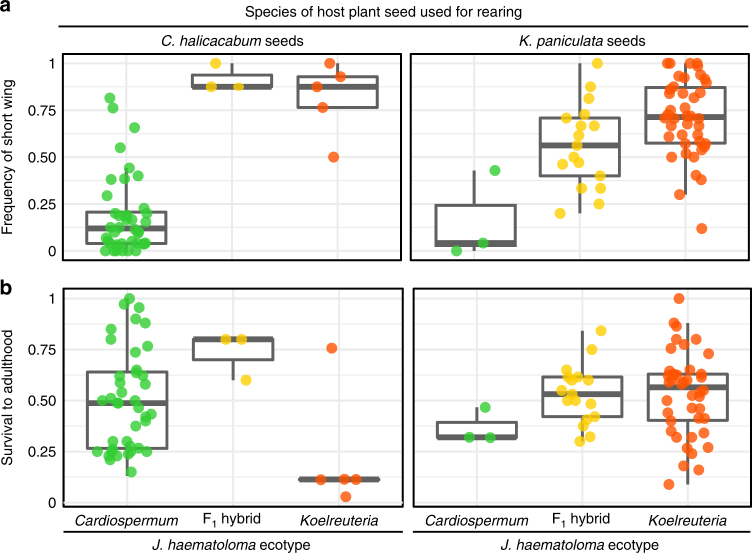
The effects of cross-rearing *J. haematoloma* on non-natal host plant seeds. **a** Wing morph frequencies were not significantly affected by cross-rearing. Treatments included in the natal groups (e.g., *Cardiospermum* bugs raised on *Cardiospermum* seeds) are taken from the full dataset with values of food availability comparable to the cross-reared treatments. **b** Survival to adulthood was reduced when bugs were raised on seeds of non-natal hosts. The distribution of values is shown by Tukey’s plots: boxes demarcate the upper and lower quartiles, whereas the heavy bar indicates the median value. Whiskers extend to 1.5 times the interquartile range or the most extreme value. Each superimposed point represents one cohort of multiple bugs

In the previous analysis, bugs were raised on seeds of the host plant associated with their ecotype. Therefore, we tested for potential effects that the host plants might have on morph determination by cross-rearing juveniles on non-natal seeds (Fig. 3). The resulting offspring were examined using permutation-based factorial ANOVA, considering the effects of ecotype and seed species on the frequency of short-wing morphs. Although the influence of ecotype on morph frequencies was significant (overall model main effect for ecotype, *F*_1,94_ = 47.24, *p* < 2 × 10^−16^), cross-reared bugs of each ecotype did not differ significantly in morph frequency (main effect of seed species, *F*_1,94_ = 0.635, *p* = 0.38; Fig. 3a, compare left and right panels for each ecotype). Interestingly, survival to adulthood was reduced for bugs raised on non-natal seeds (Fig. 3b). This decrease in survival was significant for *Koelreuteria*-adapted bugs raised on *Cardiospermum* seeds (overall model interaction of ecotype and seed species, *F*_1,94_ = 6.85, *p* = 6.00 × 10^−3^; two-sample permutation test for *Koelreuteria* ecotype, *Z* = − 2.90, *p* = 3.72 × 10^−3^).

The response of F_1_ hybrids from crosses of *Cardiospermum* and *Koelreuteria* ecotypes suggest that host plants can exert influence on wing morph frequency. Among F_1_ hybrids, the host seed species used for rearing was a significant factor in wing morph ratios (exact permutation, *p* = 9.12 × 10^−4^). However, the direction of this effect is opposite that seen for host-associated ecotypes, with more long-wing adults produced when hybrid bugs are raised on *K. paniculata* seeds. Survival of hybrids was higher on both seed species than for cross-reared parental ecotypes, although this difference is marginal (exact permutation, *p* = 0.0714). The reduced survival of hatchlings on non-natal seeds may present an obstacle to the colonization of new host plant species in the wild. However, the high survival of ecotype hybrids on both plants suggests that contemporary gene flow may still be possible among ecotypes^40–42^.

### Sexual conflict in wing polyphenism varies by ecotype

**Fig. 4:**
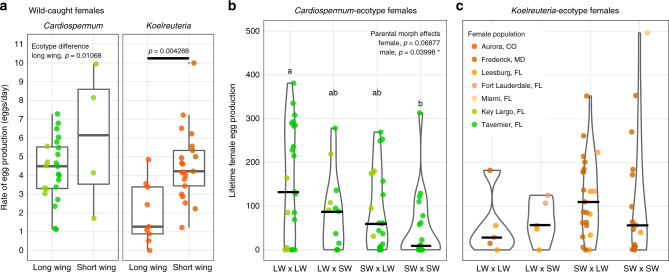
Reproductive output differs by morph and ecotype. **a** The rate of egg production among wild-caught females of different morph and ecotype is represented by individual points, colored by population of origin. Tukey’s box-whisker plots summarize the dispersion of values and the median for each group as in Fig. 3. **b**, **c** Lifetime female egg production from laboratory crosses of different morph and ecotype. The results of individual crosses are denoted by points, colored by population of origin. Violin plots summarize the dispersion of cross results. Heavy bars indicate the median outcome. Significant global effects, as determined by permutation-based ANOVA, are superimposed on the plot. Letters indicate significant difference among cross types based on post-hoc pairwise permutation tests. Abbreviations: LW, long-winged; SW, short-winged

As *J. haematoloma* ecotypes vary in their polyphenic reaction norms (Fig. 2), we sought to test whether the reproductive advantage predicted for short-winged females might vary among ecotypes. On average, for each ecotype, wild-caught short-winged females laid eggs faster than long-winged females, and this difference was significant among *Koelreuteria* ecotypes (Fig. 4a; Wilcoxon test with Bonferroni adjustment, *W* = 27, *p* = 0.00429). Among the *Cardiospermum* ecotype, the egg-laying rate was higher on average in short-winged females (Fig. 4a). Increased fecundity for short-winged females is consistent with the classic models of a dispersal-fecundity polyphenic trade-off^5,47^.

We also examined lifetime egg production from females in laboratory crosses (Fig. 4b, c). Effects on fecundity were tested using permutation-based factorial ANOVA, considering the morph and ecotype of each parent and potential interactions among morphs. Female morph was not a significant factor, although crosses of *Koelreuteria* ecotypes involving short-winged females did produce marginally more eggs (Fig. 4c; *Z* *=* −1.55, *p* = 0.12). Females raised in the lab have access to energy stores unavailable to wild females and these lab-reared long-winged females do not expend energy flying before being able to mate. For these reasons, polyphenism may still be adaptive in *Cardiospermum* ecotype females. Seed resources are typically much more widely distributed in *Cardiospermum* habitats^36^, suggesting a greater advantage to the dispersal ability conferred by flight.

It has been unclear whether wing polyphenisms in different species might be adaptive for males, or if male flightlessness represents a sexual conflict produced by pleiotropy^29,47^. Surprisingly, for the *Cardiospermum* ecotype, crosses produced significantly fewer offspring when males were short winged (Fig. 4b; permutation with Bonferroni correction, *Z* = 2.326, *p* = 0.0400). However, this effect was not found for the *Koelreuteria* ecotype, where short-wing bugs of both sexes are much more common in the wild. Differences in male fertility were not due to differences in the size of testes or accessory glands (Supplementary Fig. 6).

These results suggest a sexual conflict in wing polyphenism. Polyphenism appears to be adaptive for females, allowing faster egg production in high-nutrient environments. However, for populations on *Cardiospermum*, the polyphenism appears to be maladaptive for males, which suffer reduced fertility when short winged. Short-winged bugs are much more common in populations living on *K. paniculata*, which appear to have evolved a resolution to this sexual conflict, allowing more equal fitness for males of both morphs.

### Dominance of the derived reaction norm

In order to explore the underlying genetic basis for ecotype differences in nutritional reaction norms, crosses were made with virgin adults from Tavernier, Florida, which feed on *C. corindum* and have a reaction norm offset favoring long-wing morphs, and Frederick, Maryland, which live on *K. paniculata* and are more frequently short winged at comparable levels of juvenile food availability. Reciprocal crosses showed no evidence of maternal effects. Hybrid cohorts were raised with varying numbers of seeds.

If reaction norms have evolved by changes in mostly additive genetic variance, then hybrids should display an intermediate reaction norm compared with the parental ecotypes. Strong dominance, in which the F_1_ reaction norm resembles one of the parents, would be evidence that population differences in plasticity are produced by genetic differences with strong non-additive effects. The morph frequencies of F_1_ cohorts and their norm of reaction strongly resembled the *Koelreuteria* ecotype (Fig. 2, middle). We used a logistic model of morph determination in hybrid and parental populations to examine the effect of genetic background on the reaction norm (Supplementary Table 9). Compared with F_1_ hybrids, the odds that a bug will be short winged are four to seven times lower for the *Cardiospermum* parental population (95% confidence on odds ratio). In contrast, bugs from the *Koelreuteria* parental population were not significantly different in their predicted morphs from the F_1_.

Together, these results suggest that although the frequency of wing morphs is environmentally determined, the threshold for that response is determined by the genetic background. Responses of the ecotype hybrids demonstrate that this genetic difference may have evolved through changes in relatively few genes with large effect and strong dominance.

### Insulin pathway components in the soapberry bug

Insulin signaling mediates nutrition-dependent growth in various contexts^34,48,49^, making it a candidate mechanism for the evolution of nutrition-dependent polyphenism^32^. Functional tests in the planthopper *N. lugens* have also implicated the activities of paralogous insulin receptors in wing morph development in that species^31^. We assembled transcriptome sequences for each *J. haematoloma* ecotype. This allowed the identification of one-to-one orthologs for most insulin pathway components. Two genes encoding orthologs of the *Drosophila* insulin receptor were identified in *J. haematoloma* (Supplementary Fig. 7), as well as from the boxelder bug *Boisea trivittata* (Rhopalidae) and the milkweed bug *Oncopeltus fasciatus* (Lygaeidae). One insulin receptor ortholog (*InR1*) was also isolated from a species of monomorphic soapberry bug, *J. sanguinolenta*.

In order to explore whether insulin signaling components differ in their expression by morph, we measured transcript abundance in nascent adults using quantitative PCR (qPCR). At this stage, bugs are undergoing sexual maturation, making gene expression relevant to fecundity. The ovaries of nascent adult females showed differences in the expression of the insulin receptor genes by ecotype (Supplementary Fig. 8b, c). The relative expression of *FoxO*, a transcription factor downstream of the insulin signaling pathway^48^, also differed by morph among females of the *Cardiospermum* ecotype (Supplementary Fig. 8a). In the absence of insulin-like peptides, FoxO inhibits expression of genes related to protein synthesis and cell division^50,51^, while promoting its own transcription and that of the insulin receptor^52^.

### Manipulation of insulin signaling alters the reaction norm

**Fig. 5:**
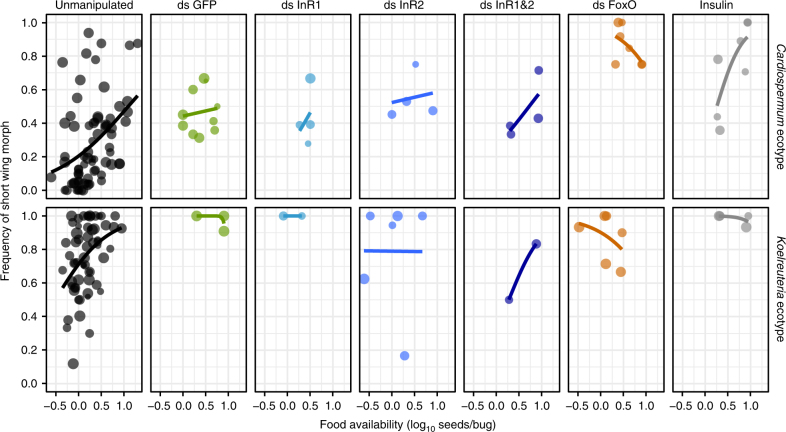
Manipulation of insulin signaling alters wing morph reaction norms. Effects of RNAi treatment on the frequency of short-wing morphs in *Cardiospermum* (top row) and *Koelreuteria* (bottom row) ecotypes. Unmanipulated bugs (left column) differ in their reaction norms based on ecotype. In the *Cardiospermum* ecotype, RNAi targeting *FoxO* or injection of exogenous insulin significantly alter the reaction norm, making it resemble the *Koelreuteria* ecotype. Knockdown of both insulin receptor genes in the *Koelreuteria* ecotype produces a marginal decrease in the rate of short-winged adults. Each point represents a treatment cohort of multiple individuals. Point size indicates relative survival rate. These data include 4218 scored adults (820 from RNAi) in 187 cohorts (59 RNAi treatments)

**Fig. 6:**
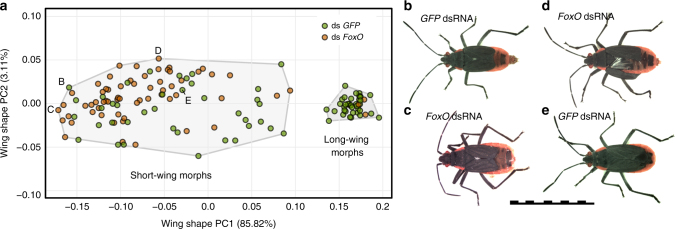
Knockdown of *FoxO* biases short-wing shapes. **a** Principle component analysis of wing shapes from *GFP* and *FoxO* RNAi specimens. RNAi treatment has a significant effect on wing shapes, with *FoxO* RNAi skewing them towards the upper left. *n* = 141. **b**–**e** Specimens representing different areas of the morphospace in panel A. *FoxO* RNAi specimens resemble extreme individuals from the control treatment. Control specimens with extreme **b** and average **e** wing shapes are shown in photographs, as are *FoxO* RNAi specimens with extreme positions on PC1 **c** and PC2 **d**. For each specimen the shape of the right (top) wing was analyzed. Warp grids representing the wing shapes of these and other specimens appear in Supplementary Fig. 14. The scale bar is 1 cm

We tested the influence of insulin signaling on *J. haematoloma* development and wing morph specification by treating fourth instars with exogenous insulin^53,54^ or via RNA interference (RNAi) targeting the insulin receptor genes (*InR1* and *InR2*), two signal transducers, *chico* and *Akt*, and *FoxO*. These experiments were conducted with juveniles raised under a range of food regimes, to examine developmental genetic control of wing morph reaction norms (Fig. 5) and wing shape (Fig. 6). RNAi knockdown was verified using quantitative reverse transcriptase-PCR (qRT-PCR) (Supplementary Fig. 9).

Activation of insulin signaling, by introduction of exogenous insulin or knockdown of *FoxO*, phenocopied the evolution of reaction norms from the high-threshold *Cardiospermum* host ecotype to resemble the *Koelreuteria* ecotype, which has a much lower threshold for short-wing development (Fig. 5a). *FoxO* RNAi significantly altered the reaction norm for wing morph frequencies such that they were 10–38 times more likely to be short winged than unmanipulated bugs (95% confidence intervals on odd ratio from logistic regression; Supplementary Table 12). This increase in the likelihood of the short-wing morph was significant in comparison with nonspecific *green fluorescent protein* (*GFP*) double-stranded RNA (dsRNA) controls (Wald’s test, *z* = 5.69, *p* = 1.31 × 10^−8^; Supplementary Table 13). Injection of roughly 0.5 ng insulin per mg body weight had a similar effect, increasing the odds of short-wing development by four to nine times, a significant difference from control injections (*z* = 3.51, *p* = 4.46 × 10^−4^; Supplementary Table 13). Similar effect sizes for insulin and *FoxO* RNAi were found within the *Cardiospermum* ecotype (Supplementary Table 14), although these treatments did not significantly influence wing morph frequencies in *Koelreuteria* ecotypes, where short-wing bugs are already common. Conversely, reduction of insulin signaling by RNAi targeting *InR1* and *InR2* simultaneously resulted in marginally lower frequencies of short-wing bugs among *Koelreuteria* ecotypes (*z* = − 1.818, *p* = 0.0691). In *Drosophila*, InR activation inhibits FoxO which normally acts to suppress target genes related to growth^49,51^. Therefore, these results are consistent with a model in which increased food consumption activates insulin signaling, potentially in concert with other pathways, to specify development of the short-wing morph.

Although *FoxO* RNAi during late juvenile development increases the frequency of short-wing morphs, *FoxO* expression is higher in ovaries and dorsal thorax of nascent adult short-wing morphs, compared with long-wing bugs (Supplementary Fig. 8). This apparent paradox likely reveals dynamic expression and function of *FoxO* between these different life stages.

Targeting other components of the insulin pathway by RNAi failed to alter development in obvious ways. Knockdown of *chico* and *Akt* (Supplementary Fig. 11), which encode intracellular transducers of insulin signaling, extended the fifth instar by five to ten times its normal duration, greatly extending their normal lifespans. However, this result precluded a determination of any influence these genes may have on morph specification.

### *FoxO* RNAi biases the development of wing shape

As* FoxO* RNAi significantly increased the frequency of short-wing morphs in *Cardiospermum* ecotypes (Fig. 5 and Supplementary Tables 13, 14), we examined whether knockdown of this gene might also influence the shape of wings. We utilized the same geometric morphometric analysis applied to unmanipulated bugs (Fig. 1) on 77 *GFP* dsRNA-treated control specimens and 64 *FoxO* RNAi individuals. Principal component analysis of RNAi wing shapes (Fig. 6a) placed short- and long-winged bugs in a similar relationship in morphospace, compared with unmanipulated bugs (Fig. 1i). However, RNAi treatment had a significant effect on wing shape overall (Procrustes ANOVA, *F*_1,138_ = 8.66, *p* *<* 10^−4^) and among short-wing morphs (*p* *<* 2 × 10^−4^ with Bonferroni adjustment). *FoxO* RNAi short-wing shapes occupied a similar region of morphospace as control wings, but the *FoxO*-knockdown group was biased toward extreme short-wing shapes. These wings were more severely reduced in the distal membrane region (low PC1 values in Fig. 6a, Fig. 6c, d compared with 6e, Supplementary Fig. 14) and narrower in the anterior–posterior dimension (high PC2 values). RNAi treatment did not have a significant effect on long-wing shapes; however, the power of this inference is limited by the small number of long-wing *FoxO* specimens (*n* = 3) in the shape analysis.

Other appendages were also examined for allometric effects of RNAi targeting *FoxO* and *InR1*. No significant effects were found for the relative lengths of antennae, the labrum, labium (beak), femora of the legs, and wings (Supplementary Note 1). However, body length and pronotum width were affected by knockdown of these genes, with *InR1* RNAi decreasing relative size and *FoxO* RNAi increasing relative size (Supplementary Fig. 12).

## Discussion

Genes act in an environmental context to determine phenotypes and the mechanisms by which environmental conditions exert an influence on development are subject to evolution^3,19,22,23,25,46^. Therefore, reaction norms can evolve over time, vary among populations, and adapt under the influences of selection. Here we have examined nutritionally dependent plasticity in the determination of wing morphs in the soapberry bug *J. haematoloma*, characterizing morphology, population-level variation in plasticity, and fitness implications, as well as developmental mechanisms.

Variation in the length and shape of *J. haematoloma* wings is discontinuous (Fig. 1), constituting two discrete morphs. Short-wing morphs display a significantly wider range of wing shapes than long-winged morphs (Fig. 1i). This difference may be non-adaptive, resulting from different degrees of canalization in each developmental trajectory. Alternatively, greater selection on long wings imposed by the need for flight performance may limit their range of shapes. A third explanation for greater short wing disparity is that the differing prevalence of the two morphs exposes them to different selection intensities. This last hypothesis predicts that ecotypes differing in the frequency of a morph should also have reduced disparity in the more common morph. Although short-wing morphs of the two ecotypes differ slightly, but significantly in disparity, long-wing morphs have a highly significant difference in the disparity of their wing shapes. Wing-shape disparity is lowest among *Cardiospermum*-associated long-wing bugs, which are the most common morph in that ecotype and which are used by the bugs for flight. These results suggest frequency-dependent selection on wing shapes among long-wing morphs.

There is strong support for an explanation of morph frequencies based on juvenile food availability. Raising juvenile cohorts of *J. haematoloma* under different resource conditions reveals differing reaction norms for two host-associated ecotypes (Fig. 2). Differences in these reaction norms appear to be genetically based, as evidenced by the persistence of the response when bugs are cross-reared on a non-natal host (Fig. 3) and by dominance of the reaction norm in F_1_ ecotype hybrids (Fig. 2).

Among ecotype hybrids, a higher frequency of short-wing morphs was produced when bugs were fed *Cardiospermum* seeds compared with rearing on *Koelreuteria* seeds. Interestingly, the direction of these hosts’ effect on hybrid wing morph frequencies is counter to the normal bias in morph frequencies displayed by the parental ecotypes adapted to those hosts. This suggests that compared with the F_1_ genetic background, the reaction norms for wing morph determination in each parental, host-adapted ecotype have been modified by selection over the generations since their divergence.

A maladaptive response to nutritional conditions in hybrids may present obstacles to gene flow between ecotypes. However, a recent study of *J. haematoloma* comparing beak length, egg weight, and developmental time among ecotypes in 2014 with studies ca. 1990 found indirect evidence of maladaptive gene flow from the more numerous *Koelreuteria* ecotype into *Cardiospermum*-associated populations in south Florida^40,42^. Poor survival of *Koelreuteria* ecotypes on *Cardiospermum* seeds suggests that secondary colonization of the ancestral native host by derived ecotypes may be unlikely without hybridization first.

The *Koelreuteria* ecotype of *J. haematoloma* has evolved to be predominantly short winged, which may reflect selection to allocate resources to increasing fecundity in this environment. *Koelreuteria* are larger plants with a higher abundance of seeds and higher lipid content than *Cardiospermum*. These factors make dispersal by flight potentially less advantageous on this host^47^, which may explain the differences in ecotype reaction norms. Short-winged females of the *Koelreuteria* ecotype produce eggs faster than long-winged females (Fig. 4a), helping to maximize fitness in a relatively stable, high-nutrient environment.

It has been unclear whether male wing polyphenism has any adaptive value in *J. haematoloma* or other species. In contrast, we find that in the *Cardiospermum* ecotype, where the short-wing morph is rare, short-winged males suffer reduced fitness, as crosses with these sires produced fewer offspring on average (Fig. 4b). Short-winged males are also unable to disperse by flight. These results suggest a sexual conflict, where males maximize fitness consistently with development to the long-winged morph, which provides higher fertility and the opportunity to disperse. Meanwhile females may produce the most surviving offspring by determining their morph based on local food availability. The threshold for short-wing development is lower and short-wing morphs are more common in both sexes of the *Koelreuteria* ecotype. In this ecotype, the male morph does not influence the fecundity of a cross. Therefore, it is likely that in adapting to *Koelreuteria*, soapberry bugs evolved a resolution to this sexual conflict, allowing wing polyphenism to remain unlimited by sex without reduced fitness in short-winged males.

Changes in expression (Supplementary Fig. 8) of insulin signaling components in *J. haematoloma* have accompanied the evolution of the reaction norm for wing morph determination among host ecotypes (Fig. 2). Activation of insulin signaling by exogenous insulin injection or *FoxO* RNAi can phenocopy this evolutionary change (Fig. 5). Insulin-like peptides are produced in the insect brain^55,56^ in response to feeding^55^. In fruit flies, insulin signaling regulates cell proliferation and protein synthesis^56,57^. Therefore, insulin signaling may provide a physiological mechanism by which individual soapberry bugs could gauge their nutritional resources in order to coordinate growth. In other insects, tissue-specific allometries result from the local levels of expression and activation of insulin pathway components^33,34,58–60^. Differences in insulin signaling have been identified in sex-specific allometric differences and in sex-limited plasticity in organ growth^33,61^.

Insulin signaling appears to regulate wing polyphenism in *J. haematoloma* differently than in the brown planthopper *N. lugens* (Hemiptera: Delphacidae)^31^. The cues determining wing morphs in *N. lugens* are not known, although nutrition, stress, and crowding have been suggested as causes^62^. In the planthopper, depletion of *InR1* and *InR2* cause development of almost entirely short- or long-wing morphs, respectively. Knockdown of *N. lugens FoxO* produces predominantly long-winged planthoppers, the opposite of the effect described here for *FoxO* RNAi in *J. haematoloma*. The reason for these dramatic differences is unclear and, although wing polyphenism is widespread among true bugs, rhopalids and delphacids diverged ~ 320 mya^63^. Further investigation of polyphenic regulation in these and other species will be necessary to identify themes common to Hemiptera.

The soapberry bug *J. haematoloma* is a promising model species for the study of polyphenic development and its evolution. Our data suggest that in this species, alternative morphs offer trade-offs maximizing either dispersal ability through flight or increased fecundity. Determination of morphs appears to be based on juvenile food availability and this reaction norm has diverged with adaptation to a novel host plant. Wing polyphenism in *J. haematoloma* is not sex limited. In the ancestral ecotype, short-winged males are rare and appear to suffer reduced fertility. The derived ecotype appears to have evolved a lower offset to the reaction norm, increasing the frequency of short-wing adults of both sexes. Males of this group have similar fitness, regardless of morph, in an apparent resolution to the sexual conflict. Finally, our results demonstrate that artificial activation of insulin signaling can phenocopy evolution of the derived reaction norm. Therefore, we suggest that changes in the insulin signaling pathway may underlie reaction norm evolution in this wild population. The involvement of insulin signaling in diverse developmental contexts, from wing polyphenism in planthoppers^31^ to polyphenism in sexually competitive weapons^33,64^ supports the hypothesis^32^ that this pathway is a hotspot for the evolution of discontinuous organ growth regulated by nutritional cues.

## Methods

### Insect culture

*Jadera haematoloma* were collected from host plants at several locations, including Tavernier, FL (January 2013 and February 2015), Key Largo, FL (February 2015), Aurora, CO (August 2015), and Frederick, MD (October 2014). Founding cohorts consisted of 50–100 individuals, and multiple cultures from each population-of-origin are maintained with similar numbers each generation. In the lab, soapberry bugs were raised in an incubator at 26 °C in 3.38 L plastic terrariums (Carolina Biological Supply Company, Burlington, North Carolina, USA). A tray of water was placed in the incubator to increase humidity. Spring water was continuously available in glass flasks with a paper towel wick. A 6-cm Petri dish containing a folded paper towel was wetted every 1–3 days to maintain high humidity. Bugs were fed either seeds of *K. paniculata* (F.W. Schumacher Co., Inc., Sandwich, Massachusetts, USA) or *Cardiospermum halicacabum* (Outsidepride.com, Inc., Independence, Oregon, USA). Due to the prevalence of cannibalism among hatchlings, eggs were removed and kept in Petri dishes until hatching, after which juveniles were removed to a separate container. Under these conditions, the generation time is 6–10 weeks. Ontogenetic allometry was assessed by raising bugs in isolation in 6-cm Petri dishes containing a folded paper towel, wetted daily, and exactly three seeds.

For the determination of reaction norms, soapberry bugs were collected from hatching dishes as second instars. Precise numbers of bugs and seeds of their natal host plant were combined in a terrarium or other container of known volume, with the water resources described above. Bugs were incubated at 26 °C until all juveniles molted to adulthood or died. Water was replaced daily, but no food was removed or added. Dead bugs were removed periodically. All adults were scored for sex and morph, and in most treatments individuals were imaged as vouchers and for morphometric analysis.

### Morphometric analysis

The sizes and shapes of *J. haematoloma* wings were analyzed using landmark-based geometric morphometric methods^43^ as implemented in the R package geomorph^44^. Bugs were anesthetized using CO_2_ and imaged on a trinocular stereo microscope (VWR International, Radnor, Pennsylvania, USA) with a Moticam 5 digital camera (Richmond, British Columbia, Canada). A millimeter-scale ruler was included in images to provide scale. Dorsal and ventral images of each specimen were recorded. Using ImageJ v1.46r5 linear pixel measurements were obtained for the lengths of body, labrum, labium, antennae, and femora of each leg. These values were then converted to metric distance using measurements of the scale. Twenty-four anatomical landmarks were placed on dorsal images of the wing (Fig. 1a, described in Supplementary Note 1). Generalized Procrustes analysis with partial Procrustes superimposition was preformed using minimized bending energy (Fig. 1b). Scaling coefficients of allometry were calculated from regression of log wing length to log head width relationships (Supplementary Figs. 1 and 3), as described by Huxley^1^. Modularity hypotheses were tested using a covariance ratio coefficient^45^ (Fig. 1c) in 1000 permutations. Morphological disparity within each morph was assessed using a permutation test of pairwise Procrustes distances with one million iterations. Tangent space positions for Procrustes-aligned specimens were determined along principal component axes (Fig. 1i). Procrustes ANOVA with permutation^44^ was used to assess hypotheses for patterns of shape variation among the aligned specimens, using 10,000 iterations.

### Fecundity assessments

Fecundity was assessed for isolated, wild-caught *J. haematoloma* females and for individuals paired in crosses. In both cases, bugs were kept in plastic Petri dishes of 10 cm diameter and 2.5 cm deep. So that food availability did not limit female egg production, water and at least ten seeds of the natal host species were provided in uncovered 3.5 cm diameter Petri dishes. Seeds were replaced if they grew mold. Eggs were counted and removed within 7 days, before hatching. We could not control the age of wild-caught females. Therefore, we kept females in isolation and determined their rate of egg-laying after capture, from the first day eggs were produced until the last day eggs were produced.

### Examination of testes and accessory glands

In an attempt to identify the mechanism for reduced fecundity in short-winged *Cardiospermum*-ecotype males, we examined the relative sizes of the testes and accessory glands from males of each ecotype. These organs were dissected from adult males at least 5 days old. The sizes of these organs were measured as their photomicrographic area.

### Isolation of candidate genes

A reference transcriptome was prepared from 12 nascent adults of the *Cardiospermum* ecotype from Tavernier, FL, and 12 adults from *K. paniculata* in Aurora, CO. RNA was isolated from whole bodies and sent to Beckman Coulter Genomics (Danvers, Massachusetts) for poly-A selection, preparation of TruSeq libraries, and sequenced using Illumina HiSeq for 125 bp paired-end reads. Reads from all samples were trimmed^65^ and assembled using Trinity^66^. The resulting transcriptome contained 258,322 contigs (*N*_50_ = 1596 bp; 89.8% BUSCO score^67^). Reciprocal BLAST searching was used to identify sequences orthologous to candidate genes of interest. Fragments of the candidate genes, *InR1*, *InR2*, *chico*, *Akt*, and *FoxO* were isolated by PCR or constructed by commercial Gibson assembly (Integrated DNA Technologies, Inc., Coralville, Iowa). PCR primers are listed in Supplementary Table 7. PCR products were ligated into the pCR4-Topo vector (ThermoFisher Scientific, Waltham, Massachusetts) and transformed into One Shot TOP10 chemically competent cells (ThermoFisher Scientific). Plasmids were then isolated using the Purelink Quick Plasmid Miniprep kit (ThermoFisher Scientific) and sequences were confirmed by Sanger sequencing (Beckman Coulter Genomics). Sequences for *Akt*, *Chico*, and *InR2* were obtained from assembled transcripts identified by BLAST homology and used for de novo Gibson assembly of linear DNAs (Integrated DNA Technologies, Inc.).

### Orthology assignments

Most candidate genes had single orthologs in *J. haematoloma* that could be unambiguously identified using BLAST searches. However, because of the possibility of confusion among members of the insulin receptor family, we determined orthology for these genes using phylogenetic inference. Predicted insulin receptor-like protein sequences were obtained from transcriptomes of three additional hemipterans, *Jadera sanguinolenta*, the box elder bug *B. trivittata*, and the milkweed bug *O. fasciatus*^68^, and from GenBank accessions for the fruit fly *Drosophila melanogaster* (P09208), the red flour beetle *Tribolium castaneum* (AHF20214; EFA02828), the pea aphid *Acyrthosiphon pisum* (XP_001952079; XP_001942660), and the brown planthopper *N. lugens* (AIY24638; AIY24639). Two putative insulin receptors were obtained from most of these species, except for *D. melanogaster* and *J. sanguinolenta*, from which only one InR was identified by BLAST. Four putative receptor tyrosine kinase sequences from *B. trivittata* and *J. sanguinolenta* were used as out-groups. Predicted amino acid sequences were aligned using ClustalW in Geneious 7.1.7 (Biomatters Ltd, Auckland, New Zealand) and manually trimmed to 1268 positions. The consensus protein tree was inferred using MrBayes v3.2.6. Amino acid substitution rates were modeled using the BLOSUM matrix, Γ-distributed rate variation across sites and a proportion of sites being invariable. Markov chain Monte Carlo analysis was run for 5 million generations with a 10% burn-in on a multi-processor core using 30 CPUs.

The consensus topology (Supplementary Fig. 7) strongly supports two paralogous groups, designated *InR1* and *InR2*, following Xu et al.^31^. All insulin receptors were strongly monophyletic. The single InR protein from *D. melanogaster* did not nest with either paralog group. As only one functional insulin receptor has been identified in the fruit fly, it is possible that its position in this tree results from a unique history of selection. The single *InR* sequence from *J. sanguinolenta* was strongly supported as orthologous to *InR1* from other species.

### Gene expression

Gene expression and RNAi validations were determined using quantitative real-time RT-PCR (qPCR). For each gene, exact primers were designed using the Primer3 algorithm, avoiding conserved functional domains and dsRNA regions. Whole nascent adults were frozen in liquid nitrogen and stored at −80 °C before homogenization and total RNA extraction. If necessary, dissection was performed after flash freezing in RNAlater. Multiple biological replicates were included for each sex and morph. Using a poly-dT primer, complementary DNA was prepared from 1 μg of total RNA using the iScript Select cDNA Synthesis Kit (BioRad, Hercules, California, USA). Gene expression was measured separately in the gonads and in the dorsal thorax, including forewings, hindwings, and flight muscle.

qPCR was performed using two methods: two-step SYBR green assays, including analysis of dissociation curves, and multiplex assays using dual-labeled probes. For the SYBR Green method, reactions used iTaq Universal SYBR Green Supermix (BioRad). Custom dual-labeled probes (Sigma-Aldrich, St. Louis, Missouri, USA) were used in reactions with iQ Multiplex Powermix (BioRad). All reactions were conducted on a CFX96 Touch qPCR System (BioRad). All assays included three to six DNA standards diluted from known concentrations of cloned or synthesized gene fragments with the addition of nonspecific salmon sperm DNA (ThermoFisher Scientific). For all tissues, we measured the expression of *InR1*, *InR2*, and *FoxO*, as well as the reference gene *β-actin*. For dorsal thorax samples, we also measured expression of *Distal-less* (*Dll*); *vitellogenin* (*vit*) expression was measured in gonad samples. Assays were normalized using the expression of *β-actin*. For validation of RNAi, expression was compared between gene-specific and nonspecific *GFP* control dsRNA treatments. All analyses were performed using the means of technical triplicates and included a minimum of three biological replicates for each group under comparison.

### RNA interference

Gene function was tested during juvenile-to-adult development in *J. haematoloma* using RNAi. A DNA template was amplified from gene fragments using primers with a 20-nucleotide T7 viral promoter sequence at the 5ʹ-end. This linear DNA was used as a template in bidirectional RNA synthesis using the MegaScript T7 transcription kit (ThermoFisher Scientific). The product was treated with DNase I to remove template DNA, then annealed by cooling and purified by precipitation in cold ammonium acetate and ethanol. After resuspension in nuclease-free water, dsRNA concentrations were determined using a nanoscale spectrophotometer (GE Life Sciences NanoVue) and diluted to 2 μg/μl. Buffered dsRNA was injected into fourth instar *J. haematoloma*. Bugs were anesthetized using CO_2_. Approximately 0.2 μl of 2 μg/μl dsRNA was injected into the ventral abdomen using a pulled-glass capillary needle. Nonspecific *GFP* sequence was used as a control for the potential effects of injection wounding and nonspecific dsRNA toxicity. Knockdown of gene activity was confirmed using qRT-PCR (Supplementary Fig. 9).

### Insulin treatment

Exogenous insulin treatments were based on previous experiments from two Lepidoptera, *Manduca sexta*^53^ and *Bombyx mori*^54^. Bovine pancreatic insulin (Sigma-Aldrich) was prepared in saline buffer at 160 ng/μl. Injection was made into the abdomen of fourth instars, in the same manner used for introduction of dsRNA. This method delivered roughly 0.5 ng insulin per mg body weight.

### Statistical analysis

All statistical tests were conducted in R (version 3.4.3). Plots were also generated using R, with some modifications to graphic layout being made using Illustrator CC 2015 (Adobe Systems, San Jose, California). Details of statistical analyses are given in the Supplementary Note 1. Supplementary Data File 1 includes R markdown code to reproduce each statistical test and the original plots.

### Code availability

All analysis scripts, including R code used for the production of the main and supplementary plots, are available in Supplementary Data File 1.

### Data availability

Gene sequences are archived under GenBank accession numbers MF620038 to MF620048. All other data that support the findings of this study are archived in Supplementary Data File 1.

## Electronic supplementary material

Supplementary Information

Description of Additional Supplementary Files

Supplementary Data 1
