# Supplementary material for: Manipulation of insulin signaling phenocopies evolution of a host-associated polyphenism
Source: Nat Commun. 2018 Apr 27;9:1699. doi: 10.1038/s41467-018-04102-1 (PMC5923257; doi:10.1038/s41467-018-04102-1)
Supplement: Supplementary file 1 — Supplementary Information [file 41467_2018_4102_MOESM1_ESM.pdf]

Supplementary Information

for 'Manipulation of insulin signaling phenocopies evolution of a host-associated polyphenism'

Fawcett, et al.

Contents

Supplementary Note 1: Details of the analyses **2**
Morphometric analysis . . . 2
Wing length distribution . . . 3
Wing static allometry . . . 5
Procrustes alignment . . . 7
Modularity tests . . . 9
Disparity analysis . . . 12
Wing shape variation . . . 13
Wing shape comparisons . . . 15
Wing shape allometry . . . 16
Ontogenetic allometries . . . 18
Morph frequencies in the wild . . . 20
Modeling determination of morph frequencies . . . 23
No evidence for Mendelian determination of wing morphs . . . 23
Polyphenic reaction norms of each ecotype . . . 25
Logistic regression . . . 27
Cross-rearing . . . 30
Ecotype hybrids . . . 31
Modeling F_1 response to food availability . . . 33
Fecundity assessments . . . 34
Wild-caught female fecundity . . . 34
Single-pair crosses . . . 35
Examination of testes & accessory glands . . . 38
Isolation of candidate genes . . . 39
Orthology assignments by phylogenetic inference . . . 40
Gene expression . . . 41
Gene expression in nascent adult tissues . . . 41
Gene interactions . . . 45
Manipulation of insulin signaling . . . 47
Modeling experimental effects on morph frequencies . . . 49
RNAi targeting *chico* and *Akt* . . . 52
Effects of RNAi on allometric growth . . . 52
Effects of *FoxO* RNAi on wing shape . . . 57

Supplementary Note 2: List of associated files **61**

Supplementary Note 3: Required R Packages **62**

Supplementary References **63**

Supplementary Note 1: Details of the analyses

This document provides supplemental information on experimental and analytical methods for the manuscript by Fawcett and coauthors. These analyses were started on 2016-06-16 by Dave Angelini at Colby College. This document has been generated by R Markdown on 2018-03-27 using R version 3.4.3. All code and output from statistical analyses is shown. Code for the preparation of plots is not displayed, but can be viewed in the `Rmd` files. To repeat analyses and generation of plots, all data files and scripts should be in the local folder (see Supplementary Note 2). Most of the figures are generated directly from the R code, except for Supplementary Figures 3, 5, 8 and 9, which include photographs or graphics rendered in Adobe *Illustrator*.

Abbreviations used in the R code

- **Jhae** - *Jadera haematoloma*
- **LW** - long-winged morph
- **SW** - short-winged morph
- **BV** - *Cardiospermum* (balloon vine) or *Cardiospermum*-associated ecotypes
- **GRT** - *Koelreuteria* (goldenrain tree) or *Koelreuteria*-associated ecotypes
- **rxn** - "reaction", as in reaction norm

Morphometric analysis

Anatomical landmarks

The sizes and shapes of *J. haematoloma* wings were analyzed using landmark-based geometric morphometric methods[1] as implemented in the R package `geomorph`[2]. `ImageJ`[3] was used to place landmarks on the right wing for geometric morphometric analysis. To aid in alignment, images were rotated so that the anterior-posterior body axis was left-to-right. Images were reflected vertically if the right-side wing was covered by the left wing. The following 24 landmarks were then placed on the right wing (Figure 1a):

1. Bifurcation of the radial and medial veins
2. Maximum curvature of clavus, near the posterior tip of the mesonotum *

3-4. Two equally spaced semilandmarks along the claval margin, anterior of landmark 2 *

5. Bifurcation of the radial vein and R2 crossvein
6. Intersection of the R2 crossvein and the medial vein
7. Intersection of the cubitus vein and the first anal vein
8. One semilandmark along the claval margin, between landmarks 2 and 7 *
9. Intersection of the medial vein and the posterior crossvein
10. Intersection of the radial vein and the anterior crossvein
11. Terminus of the radial-medial crossvein
12. First branch of the anterior membrane vein
13. Maximum posterior-distal curvature of the membrane region of the wing (roughly lateral to landmark 11 when held in place on the bug) *

14-16. Three equally spaced semilandmarks along the posterior wing margin, between landmarks 7 and 13 *

17-21. Five equally spaced semilandmarks along the distal wing margin, between landmarks 11 and 13 (landmark 19 should be the distal-most point on the wing of a long-winged bug) *

22-24. Three equally spaced semilandmarks along the anterior wing margin, between landmarks 1 and 11 *

* Indicates a semilandmark

Cartesian coordinates were obtained with the `measure` feature, and copied to a text file in the TPS format[4]. These data were imported into R using a modification of the geomorph package function `readland.tps()`, which allows a scale value recorded as pixels/mm to divide all Cartesian positions, rather the default multiplication.

```
cartesian.coords <- readland.tps.scale.option("Jhae.42LM.tps",
                                              specID="ID", multiplyscale = FALSE)
```

```
##      Scale applied by division
## [1] "Specimen names extracted from line ID="
```

Landmark data were digitized from 189 adult *J. haematoloma* dorsal images.

Specimen metadata

Metadata describing specimens in the `tps` data set are stored in a separate data file.

These metadata include information of the population-of-origin for each specimen, its rearing conditions, sex, morph and linear distance measurements of all appendages.

Some linear distances of interest were not directly measured, but can be calculated from landmark positions. This includes the dorsal anterior interocular distance (`daid` in the code), which is used as a proxy of overall body size. All linear distances are recorded in millimeters.

```
distance <- function(x,y,a,b) { d <- sqrt((x-a)^2+(y-b)^2); return(d) }
for (i in 1:dim(cartesian.coords)[3]) {
  metadata$daid[i] <- distance(cartesian.coords[3,1,i],cartesian.coords[3,2,i],cartesian.coords[7,1,i],c
  metadata$pronotum[i] <- distance(cartesian.coords[11,1,i],cartesian.coords[11,2,i],cartesian.coords[1
  metadata$winglength[i] <- distance(cartesian.coords[11,1,i],cartesian.coords[11,2,i],cartesian.coords
}
metadata$morph.sex <- as.factor(with(metadata,paste(morph,sex,sep="")))
metadata$morph.sex <- factor(metadata$morph.sex, levels = c("SWm","SWf","LWm","LWf"))
```

Wing length distribution

To confirm that wing lengths are bimodal, rather than normally continuous, we examined the frequency distribution of an arbitrarily collected group of bugs, chosen to have balanced representation of each sex and morph.

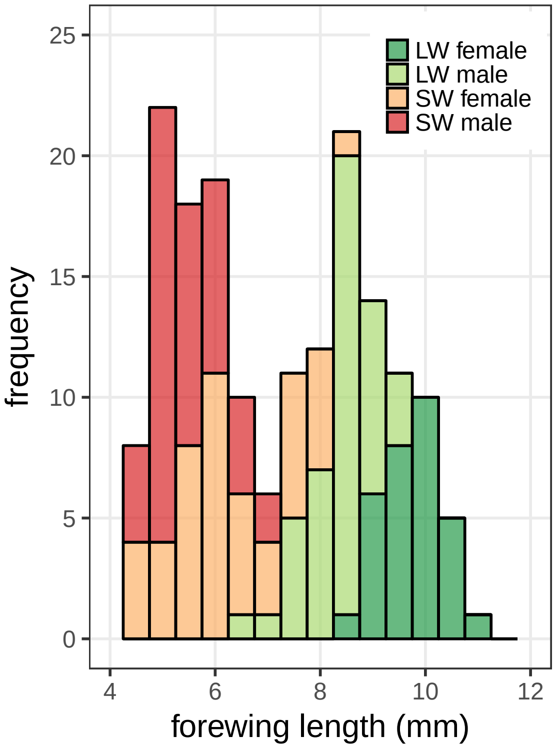

This plot was exported as a PDF (6.5 x 4 inches) and modified in *Abode Illustrator* to become Figure 1**h**. n = 168.

Adult wing lengths were not normally distributed overall (Shapiro-Wilk test, p = 2.63x10^{-6}) or within either sex (females p = 1.96x10^{-4}; males *p = 3.14x10^{-6}).

```
shapiro.test(metadata$winglength)
```

```
##
##  Shapiro-Wilk normality test
##
## data:  metadata$winglength
## W = 0.94256, p-value = 2.63e-06
```

```
with(metadata,by(winglength,sex,shapiro.test))
```

```
## sex: f
##
##  Shapiro-Wilk normality test
##
## data:  dd[x, ]
## W = 0.92479, p-value = 0.0001956
##
## --------------------------------------------------------
## sex: m
##
##  Shapiro-Wilk normality test
##
## data:  dd[x, ]
## W = 0.89752, p-value = 3.141e-06
```

Morphs differ in wing length

Wing length among morphs is significantly different. Short wing morphs are not normally distributed, as a whole or by sex. (e.g. `with(metadata,by(winglength,morph.sex,shapiro.test))`) So, comparisons are made using the Wilcoxon rank sum test.

```
with(metadata, wilcox.test(winglength ~ morph))
```

```
##
##  Wilcoxon rank sum test with continuity correction
##
## data:  winglength by morph
## W = 6893, p-value < 2.2e-16
## alternative hypothesis: true location shift is not equal to 0
```

Since sex is often a factor that influences insect body size, we tested for the effects of morph and sex on wing length using two-way permutation-based ANOVA.

```
summary(with(metadata, aovp(winglength ~ morph + sex)))
```

```
## [1] "Settings:  unique SS "
```

```
## Component 1 :
##              Df R Sum Sq R Mean Sq Iter  Pr(Prob)
## morph         1   412.63    412.63 5000 < 2.2e-16 ***
## sex           1    46.14     46.14 5000 < 2.2e-16 ***
## Residuals   165    99.80      0.60
## ---
## Signif. codes:  0 '***' 0.001 '**' 0.01 '*' 0.05 '.' 0.1 ' ' 1
```

Wing length differs significantly by both sex and morph ($p < 2.2\text{x}10^{-16}$).

Wing static allometry

The static allometry of adult wing lengths can be examined for each ecotype, morph and sex. The plot below excludes the same 21 specimens as above, plus two males of uncertain morph. The total n = 166.

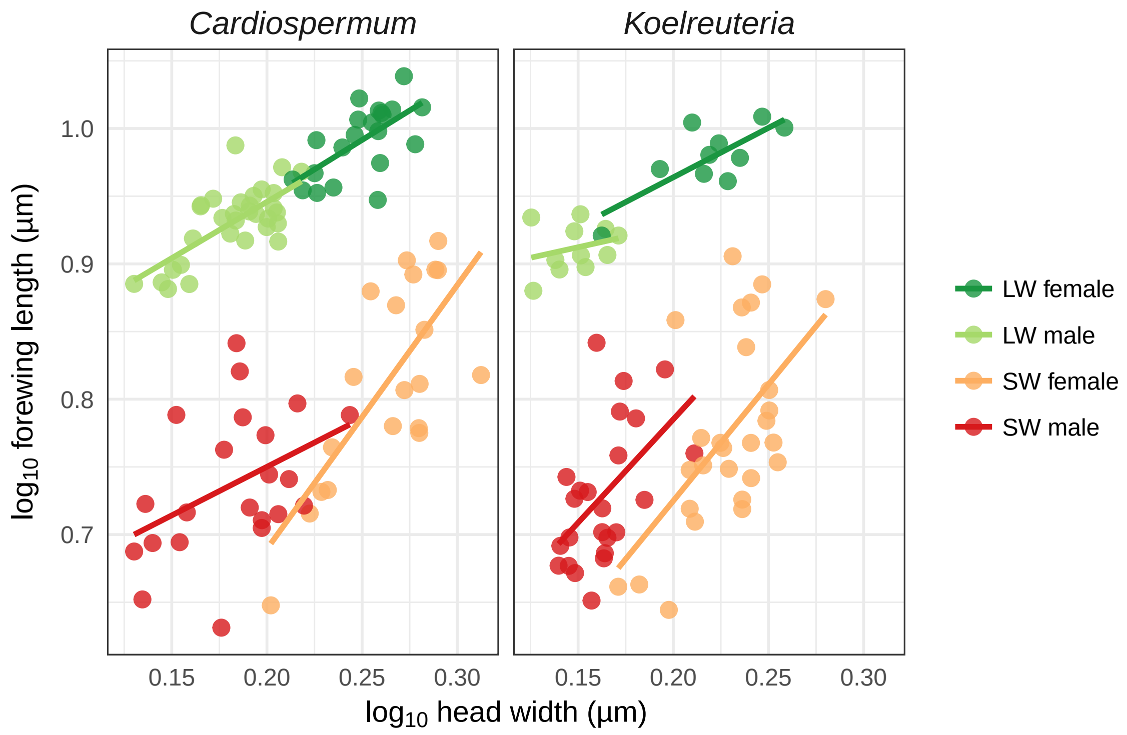

Supplementary Figure 1. Static allometry of adult wing lengths for each ecotype, morph and sex is represented on a log-log scale. Head width is used as a proxy for overall body size. n = 187.

We test for differences in scaling coefficients for static allometry by morph and ecotype using ANCOVA with a test for homogeneity of regression slopes. Since the head width (`daid`) values are different by sex, that factor can't be included in the analysis.

```
static.allom.Model <- aov(log10(winglength) ~ log10(daid) + morph + hostplant, data=metadata)
Anova(static.allom.Model, type="III")
```

```
## Anova Table (Type III tests)
##
## Response: log10(winglength)
##             Sum Sq  Df   F value Pr(>F)
## (Intercept) 3.5249   1 2022.5005 <2e-16 ***
## log10(daid) 0.2711   1  155.5451 <2e-16 ***
## morph       1.4558   1  835.2808 <2e-16 ***
## hostplant   0.0014   1    0.7871 0.3763
## Residuals   0.2823 162
## ---
## Signif. codes:  0 '***' 0.001 '**' 0.01 '*' 0.05 '.' 0.1 ' ' 1
```

Wing length is significantly associated with head width, confirming a scaling relationship exists. Morphs also differ significantly in their wing lengths. However, ecotype (`hostplant`) does not show a significant effect on wing length.

Testing for interactions between these factors and the covariate examines potential differences in the slope (allometric scaling).

```
static.allom.Model.x <- aov(log10(winglength) ~ log10(daid) + morph + hostplant
                          + log10(daid):morph + log10(daid):hostplant, data=metadata)
Anova(static.allom.Model.x, type="III")
```

```
## Anova Table (Type III tests)
```

```
##
## Response: log10(winglength)
##                        Sum Sq  Df  F value    Pr(>F)    
## (Intercept)           1.34625   1 769.4859 < 2.2e-16 ***
## log10(daid)           0.07700   1  44.0118 4.788e-10 ***
## morph                 0.09359   1  53.4961 1.176e-11 ***
## hostplant             0.00065   1   0.3699    0.5439    
## log10(daid):morph     0.00219   1   1.2503    0.2652    
## log10(daid):hostplant 0.00033   1   0.1859    0.6669    
## Residuals             0.27993 160                       
## ---
## Signif. codes:  0 '***' 0.001 '**' 0.01 '*' 0.05 '.' 0.1 ' ' 1
```

The interaction terms are not significant. Therefore, we cannot conclude scaling differs by morph or ecotype. However, it is possible that greater sampling might uncover such a relationship.

Procrustes alignment

Generalized Procrustes Analysis with Partial Procrustes Superimposition was preformed using minimized bending energy[2]. A matrix is used to define sliding semilandmarks for the wing. Each row lists the number of the semilandmark in column two. Columns 1 and 3 list the flanking landmarks.

```
wing.semilandmarks <- matrix(as.numeric(c( 1, 3, 4,
                                           3, 4, 2,
                                           2, 8, 7,
                                           7,14,15,
                                          14,15,16,
                                          15,16,13,
                                          16,13,17,
                                          13,17,18,
                                          17,18,19,
                                          18,19,20,
                                          19,20,21,
                                          20,21,11,
                                          11,22,23,
                                          22,23,24,
                                          23,24, 1))
                             , ncol=3, byrow=F)
```

The full `tps` data set includes landmarks from the dorsal body outside of the wings. Therefore the landmarks are sub-sampled to allow Procrustes alignment for wing landmarks alone.

```
wing.lm <- c(19:42) # The landmarks from the wing
wing.gpa <- gpagen(cartesian.coords[wing.lm,,],
                   curves=wing.semilandmarks,
                   ProcD=FALSE,
                   print.progress=FALSE)
# Remove centroid size for field-imaged specimens from Spring Palm Drive, Miami ("SP")
wing.gpa$Csize[which(metadata$pop=="SP")] <- NA
plot(wing.gpa)
```

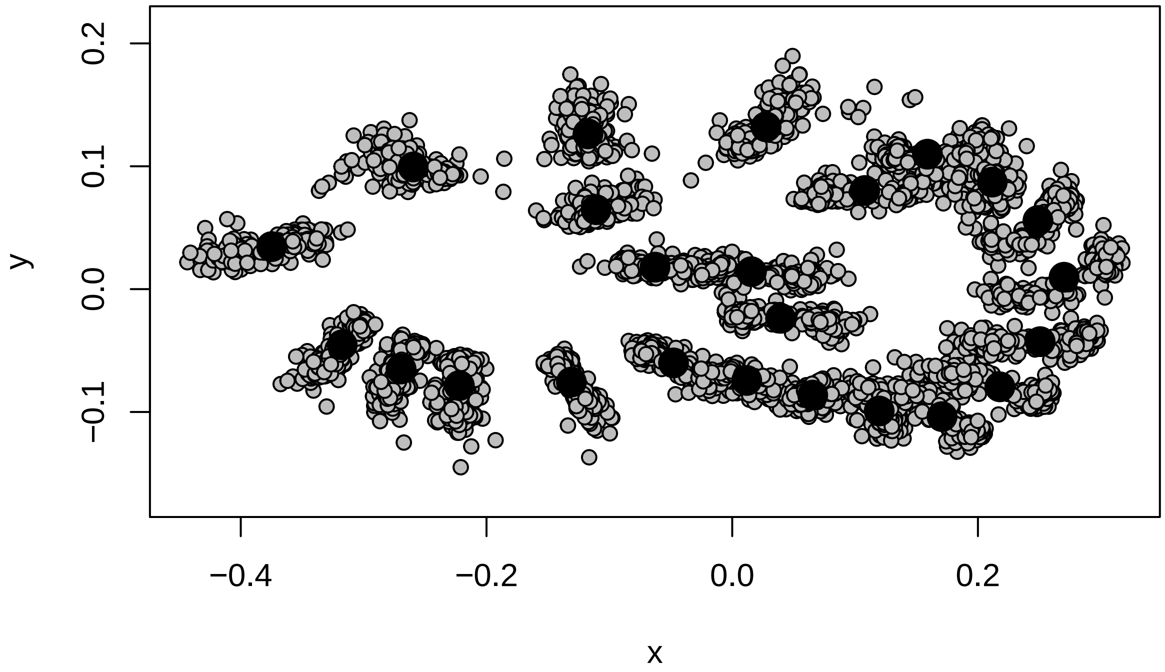

This plot appears in the main text as Figure 1**b**.

In order to compare wing shapes by different factors, we must first build a "geomorph dataframe" with the Procrustes-aligned coordinates, centroid size, and associated metadata. This data object cannot be subsetted, so separate geomorph data frames are also defined for long- and short-winged specimens.

```
gmm.metadata <- geomorph.data.frame(
  wing.gpa,
  morph = metadata$morph,
  sex = metadata$sex,
  morph.sex = metadata$morph.sex,
  pop = metadata$pop,
  ecotype = metadata$hostplant,
  beak = metadata$beak,
  antenna = metadata$antenna,
  femur1 = metadata$femur1,
  femur2 = metadata$femur2,
  femur3 = metadata$femur3,
  daid = metadata$daid,
  pronotum = metadata$pronotum,
  winglength = metadata$winglength)

subset.gpa <- function (gpa.obj, index) {
  return.obj <- gpa.obj
  return.obj$coords <- gpa.obj$coords[,,index]
  return.obj$Csize <- gpa.obj$Csize[index]
  return.obj$data <- gpa.obj$data[index,]
  return(return.obj)
}

lw.index <- which(metadata$morph=="LW")
sw.index <- which(metadata$morph=="SW")
```

```
gpa.lw <- subset.gpa(wing.gpa, lw.index)
gpa.sw <- subset.gpa(wing.gpa, sw.index)

gmm.lw <- geomorph.data.frame(
  gpa.lw,
  morph = metadata$morph[lw.index],
  sex = metadata$sex[lw.index],
  morph.sex = metadata$morph.sex[lw.index],
  pop = metadata$pop[lw.index],
  ecotype = metadata$hostplant[lw.index],
  beak = metadata$beak[lw.index],
  antenna = metadata$antenna[lw.index],
  femur1 = metadata$femur1[lw.index],
  femur2 = metadata$femur2[lw.index],
  femur3 = metadata$femur3[lw.index],
  daid = metadata$daid[lw.index],
  pronotum = metadata$pronotum[lw.index],
  winglength = metadata$winglength[lw.index])

gmm.sw <- geomorph.data.frame(
  gpa.sw,
  morph = metadata$morph[sw.index],
  sex = metadata$sex[sw.index],
  morph.sex = metadata$morph.sex[sw.index],
  pop = metadata$pop[sw.index],
  ecotype = metadata$hostplant[sw.index],
  beak = metadata$beak[sw.index],
  antenna = metadata$antenna[sw.index],
  femur1 = metadata$femur1[sw.index],
  femur2 = metadata$femur2[sw.index],
  femur3 = metadata$femur3[sw.index],
  daid = metadata$daid[sw.index],
  pronotum = metadata$pronotum[sw.index],
  winglength = metadata$winglength[sw.index])
```

Modularity tests

Several modularity hypotheses were tested against a null model in which landmarks demonstrated no modularity or integration. A permutation test of absolute values for pairwise Procrustes distances[5] was used to test the significance of each modularity hypothesis. Hypotheses focused on the distinction between the membrane region of the wing and more proximal landmarks (Figure 1c), because the membrane appears subjectively to be the region of greatest difference between morphs.

1. Proximal wing vs. membrane, including anal ridge (landmarks 1-8, 22-24 vs. 9-21)
2. Proximal/posterior wing vs. membrane and costal margin (landmarks 1-8 vs. 9-24)
3. Proximal wing, including anal ridge vs. membrane (landmarks 1-10,12,22-24 vs. 11,13-21)
4. Proximal wing vs. distal margin (landmarks 1-16,22-24 vs. 17-21)
5. Posterior vs. anterior wing (landmarks 1,5-6,9-11,19-24 vs. 2-4,7-8,12-18)

Each test calculated Adam's covariance ratio coefficient[5] using 10,000 permutations via the `modularity.test` function in the `geomorph` package.

```
i <- 999 # Set number of iterations (Adams' algorithm adds 1 iteration)
# Hypothesis 1 : membrane (including anal ridge LMs) v. proximal wing
```

```r
module1.lm <- c(rep('a',8),rep('b',13),rep('a',3))
MT1 <- modularity.test(wing.gpa$coords,module1.lm,CI=TRUE,iter=i, print.progress=FALSE)
summary(MT1)
```

```
##
## Call:
## modularity.test(A = wing.gpa$coords, partition.gp = module1.lm,
##     iter = i, CI = TRUE, print.progress = FALSE)
##
##
##
## CR: 1.0356
##
## P-value: 0.014
##
## Based on 1000 random permutations
##
## Confidence Intervals 1.0285
##
## Confidence Intervals 1.0407
```

```r
# Hypothesis 2 : membrane and costal wing v. proximal/posterior
module2.lm <- c(rep('a',8),rep('b',16))
MT2 <- modularity.test(wing.gpa$coords,module2.lm,CI=TRUE,iter=i, print.progress=FALSE)
summary(MT2)
```

```
##
## Call:
## modularity.test(A = wing.gpa$coords, partition.gp = module2.lm,
##     iter = i, CI = TRUE, print.progress = FALSE)
##
##
##
## CR: 1.0344
##
## P-value: 0.008
##
## Based on 1000 random permutations
##
## Confidence Intervals 1.025
##
## Confidence Intervals 1.0418
```

```r
# Hypothesis 3 : membrane v. proximal (including anal ridge landmarks)
module3.lm <- c(rep('a',10),'b','a',rep('b',9),rep('a',3))
MT3 <- modularity.test(wing.gpa$coords,module3.lm,CI=TRUE,iter=i, print.progress=FALSE)
summary(MT3)
```

```
##
## Call:
## modularity.test(A = wing.gpa$coords, partition.gp = module3.lm,
##     iter = i, CI = TRUE, print.progress = FALSE)
##
##
##
```

```
## CR: 1.0515
##
## P-value: 0.708
##
## Based on 1000 random permutations
##
## Confidence Intervals 1.0493
##
## Confidence Intervals 1.0534
```

```r
# Hypothesis 4 : distal margin v. proximal wing
module4.lm <- c(rep('a',16),rep('b',5),rep('a',3))
MT4 <- modularity.test(wing.gpa$coords,module4.lm,CI=TRUE,iter=i, print.progress=FALSE)
summary(MT4)
```

```
##
## Call:
## modularity.test(A = wing.gpa$coords, partition.gp = module4.lm,
##     iter = i, CI = TRUE, print.progress = FALSE)
##
##
##
## CR: 1.0748
##
## P-value: 0.632
##
## Based on 1000 random permutations
##
## Confidence Intervals 1.0733
##
## Confidence Intervals 1.0761
```

```r
# Hypothesis 5 : anterior v. posterior
module5.lm <- c('a',rep('b',3),'a','a','b','b',rep('a',3),rep('b',7),rep('a',6))
MT5 <- modularity.test(wing.gpa$coords,module5.lm,CI=TRUE,iter=i, print.progress=FALSE)
summary(MT5)
```

```
##
## Call:
## modularity.test(A = wing.gpa$coords, partition.gp = module5.lm,
##     iter = i, CI = TRUE, print.progress = FALSE)
##
##
##
## CR: 1.0434
##
## P-value: 0.094
##
## Based on 1000 random permutations
##
## Confidence Intervals 1.04
##
## Confidence Intervals 1.0466
```

As predicted, the membrane and more proximal regions of the wing displayed significant modularity (Hypotheses 1 and 2, $p < 0.05$), while other groupings of wing landmarks did not. These hypotheses and results

are summarized in the manuscript in Figure 1c.

Disparity analysis

Morphological disparity within each morph was assessed using a permutation test of absolute values for pairwise Procrustes distances, implemented with the `morphol.disparity` function using 1 million iterations.

```
i <- 1e6-1 # Set number of iterations for morphol.disparity
morphol.disparity(coords ~ morph, iter=i, data=gmm.metadata, print.progress=FALSE)
```

```
##
## Call:
## morphol.disparity(f1 = coords ~ morph, iter = i, data = gmm.metadata,
##     print.progress = FALSE)
##
##
##
## Randomized Residual Permutation Procedure Used
## 1e+06 Permutations
##
## Procrustes variances for defined groups
##         LW         SW
## 0.00214383 0.01081386
##
##
## Pairwise absolute differences between variances
##            LW         SW
## LW 0.00000000 0.00867003
## SW 0.00867003 0.00000000
##
##
## P-Values
##       LW    SW
## LW 1e+00 1e-06
## SW 1e-06 1e+00
```

Next, we tested whether disparity differs by ecotype within each morph.

```
morphol.disparity(coords ~ ecotype, iter=i, data=gmm.sw, print.progress=FALSE)
```

```
##
## Call:
## morphol.disparity(f1 = coords ~ ecotype, iter = i, data = gmm.sw,
##     print.progress = FALSE)
##
##
##
## Randomized Residual Permutation Procedure Used
## 1e+06 Permutations
##
## Procrustes variances for defined groups
##          BV         GRT
## 0.008480025 0.012264129
##
##
```

```
## Pairwise absolute differences between variances
##               BV         GRT
## BV  0.000000000 0.003784104
## GRT 0.003784104 0.000000000
##
##
## P-Values
##            BV      GRT
## BV  1.000000 0.041851
## GRT 0.041851 1.000000
```

Disparity among wings of short-winged bugs is significantly greater among *Koelreuteria* ecotypes.

```
morphol.disparity(coords ~ ecotype, iter=i, data=gmm.lw, print.progress=FALSE)
```

```
##
## Call:
## morphol.disparity(f1 = coords ~ ecotype, iter = i, data = gmm.lw,
##     print.progress = FALSE)
##
##
##
## Randomized Residual Permutation Procedure Used
## 1e+06 Permutations
##
## Procrustes variances for defined groups
##          BV         GRT
## 0.001639380 0.002732339
##
##
## Pairwise absolute differences between variances
##               BV         GRT
## BV  0.000000000 0.001092959
## GRT 0.001092959 0.000000000
##
##
## P-Values
##            BV      GRT
## BV  1.000000 0.001689
## GRT 0.001689 1.000000
```

Disparity is significantly different among long-winged bugs of each ecotype, with *Cardiospermum* bugs having less shape variation. Since long-winged bugs are more common among the *Cardiospermum* ecotype, this result is consistent with the hypothesis that this morph experiences greater exposure to purifying selection in this ecotype, which has narrowed the shape variation among those bugs.

Wing shape variation

Wing shape variation can be described by projection into Kendall's shape space and orientation along principal component axes (Figure 1i).

```
group.color <- morph.sex.colors # c("#1a9641", "#a6d96a", "#fdae61", "#d7191c")
names(group.color) <- levels(metadata$morph.sex)
group.color <- group.color[match(metadata$morph.sex, names(group.color))]
wing.pca <- plotTangentSpace(wing.gpa$coords, warpgrids = T, groups = group.color)
```

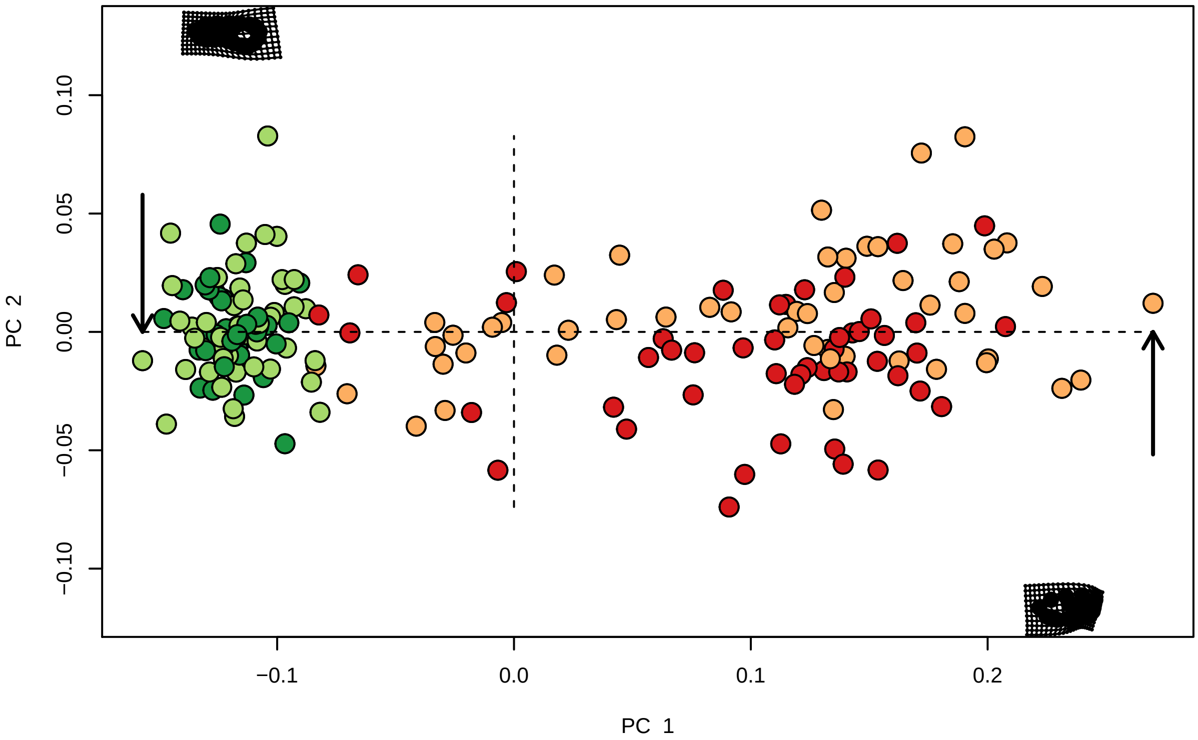

The first PC axis corresponds to the major differences in shape between long and short wing bugs. This plot was flipped along its x-axis in *Adobe Illustrator*, so that "short" wing shapes were to the left and "long" wing shapes are to right. Convex hulls were added manually to highlight the morphospace occupied by each morph. The image appears as Figure 1**i** in the main text There is no obvious separation by sex or by population of origin.

The proportion of variance explained by each PC axis can be calculated.

```
pvar <- (wing.pca$sdev^2)/(sum(wing.pca$sdev^2))
round(pvar[1:5],4) # The variation explained by the first 5 PC axes.
```

```
## [1] 0.8330 0.0314 0.0244 0.0220 0.0161
```

The majority of the variation (83.3%) is explained by PC1.

To provide examples of typical wings shapes for each morph and sex, we use the `findMeanSpec` function.

```
findMeanSpec(wing.gpa$coords) # The most average specimen (Fig. 1a)
```

```
## 334_SWf_Jhae_PK15_DRA
##                   112
```

```
findMeanSpec(wing.gpa$coords[,,which(gmm.metadata$morph=="LW" & gmm.metadata$sex=="f")]) # Fig. 1f
```

```
## 139_LWf_Jhae_PK15_DRA
##                    23
```

```
findMeanSpec(wing.gpa$coords[,,which(gmm.metadata$morph=="LW" & gmm.metadata$sex=="m")])
```

```
## 140_LWm_Jhae_PK15_DRA
##                    32
```

```
findMeanSpec(wing.gpa$coords[,,which(gmm.metadata$morph=="SW" & gmm.metadata$sex=="f")]) # Fig. 1e
```

```
## gdr16_13_SWf_PK15_DRA
```

```
##                     46
```

```
findMeanSpec(wing.gpa$coords[,,which(gmm.metadata$morph=="SW" & gmm.metadata$sex=="m")])
```

```
## gdr11_13_SWm_gh_DRA
##                   44
```

The most extreme wing shapes can be found using the base R functions `which.max` and `which.min` on PC1 values.

```
which.max(wing.pca$pc.scores[which(gmm.metadata$morph=="SW"),1]) # Fig. 1d
```

```
## AC15_010SWF_SMG
##              23
```

```
which.min(wing.pca$pc.scores[which(gmm.metadata$morph=="LW"),1]) # Fig. 1g
```

```
## MiamiSilverPalmDr_0459_LWm_DRA
##                               15
```

Images of these specimens were included in Figure 1 as representatives of wing shape disparity.

Wing shape comparisons

Procrustes ANOVA with permutation[2] was used to assess hypotheses for patterns of shape variation among the aligned specimens.

```
i <- 9999 # number of iterations
procD.lm(coords ~ morph * ecotype * sex, data=gmm.metadata,
         iter=i, RRPP=TRUE, print.progress=F)
```

```
##
## Call:
## procD.lm(f1 = coords ~ morph * ecotype * sex, iter = i, RRPP = TRUE,
##     data = gmm.metadata, print.progress = F)
##
## Type I (Sequential) Sums of Squares and Cross-products
## Randomized Residual Permutation Procedure Used
## 10000 Permutations
## ANOVA effect sizes and P-values based on empirical F distributions
##
##
##                    Df     SS      MS     Rsq        F      Z Pr(>F)
## morph               1 2.2710 2.27096 0.64330 335.0667 6.2216  1e-04 ***
## ecotype             1 0.0116 0.01159 0.00328   1.7099 2.7499 0.0047 **
## sex                 1 0.0112 0.01115 0.00316   1.6453 2.6447 0.0058 **
## morph:ecotype       1 0.0088 0.00880 0.00249   1.2987 2.3233 0.0163 *
## morph:sex           1 0.0083 0.00833 0.00236   1.2291 2.2672 0.0169 *
## ecotype:sex         1 0.0030 0.00300 0.00085   0.4424 0.7417 0.2270
## morph:ecotype:sex   1 0.0032 0.00316 0.00090   0.4666 0.8168 0.2095
## Residuals         179 1.2132 0.00678
## Total             186 3.5302
## ---
## Signif. codes:  0 '***' 0.001 '**' 0.01 '*' 0.05 '.' 0.1 ' ' 1
```

While wing shapes differ significantly by morph, sex and ecotype are not significant factors in wing shape.

Wing shape allometry

Shape and size are often related to one another, with the shape of a structure varying as a function of it size. This allometric relationship can be examined by permutation-based Procrustes ANOVA. This relationship can be visualized by graphing a shape metric against the log of centroid size.

```
# Repeat PCA without Miami specimens, which lack scale.
Y.gpa <- gpagen(cartesian.coords[wing.lm,,-c(1:21)], print.progress=F)
# Redefine a geomorph data frame
group.color2 <- morph.sex.colors # c("#1a9641", "#a6d96a", "#fdae61", "#d7191c")
names(group.color2) <- levels(metadata$morph.sex)
group.color2 <- group.color2[match(metadata$morph.sex[-c(1:21)], names(group.color2))]
gdf <- geomorph.data.frame(
  Y.gpa,
  morph = metadata$morph[-c(1:21)],
  sex = metadata$sex[-c(1:21)],
  morph.sex = metadata$morph.sex[-c(1:21)],
  pop = metadata$pop[-c(1:21)],
  ecotype = metadata$hostplant[-c(1:21)],
  winglength = metadata$winglength[-c(1:21)],
  group.color = group.color2
) # geomorph data frame

i <- 9999 # number of iterations
wing.allometry <- procD.allometry(coords~Csize, logsz = TRUE, data=gdf,
                                  iter=i, RRPP=TRUE, print.progress=F)
```

```
##
## Allometry Model
```

```
plot(wing.allometry, method = "RegScore", pt.col = gdf$group.color)
```

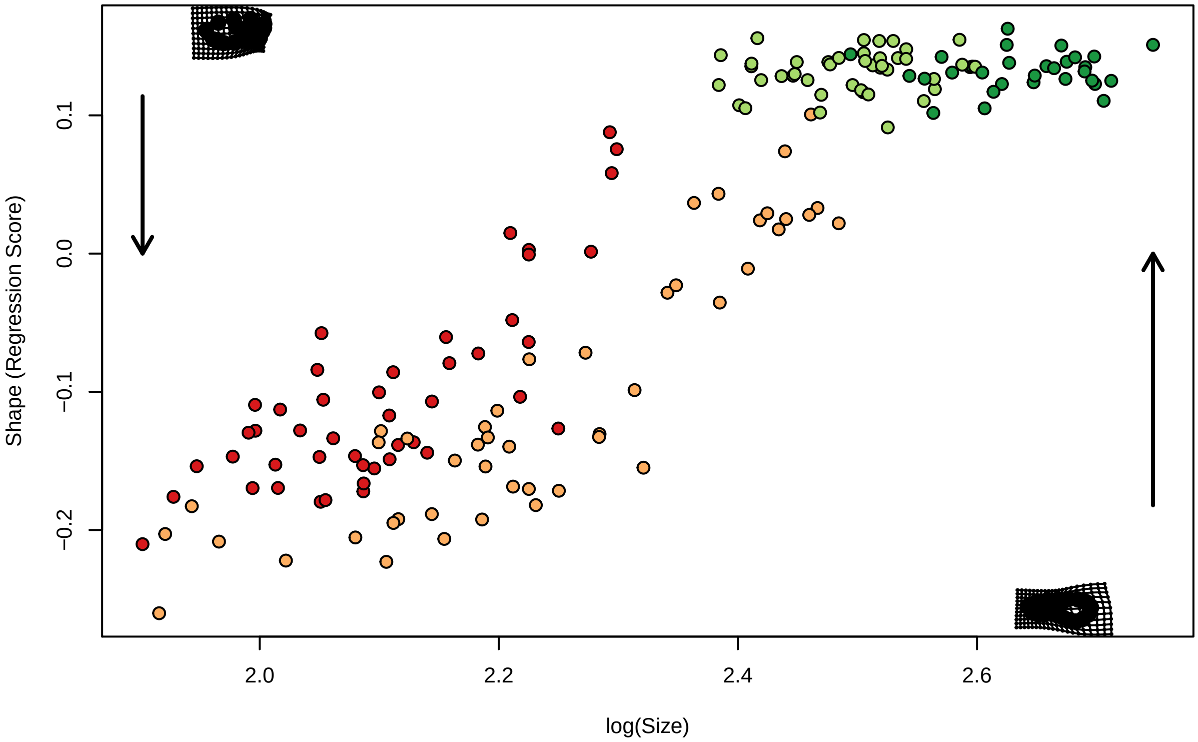

Supplementary Figure 2. Allometry curve of wing shape score regression on log centroid size. Points are colored by morph and sex as in Supplementary Figure 1.

```
wing.allometry.by.group <- procD.allometry(coords~Csize, ~morph*ecotype*sex, logsz = TRUE, data=gdf,
                                   iter=i, RRPP=TRUE, print.progress=F)
```

```
##
## Homogeneity of Slopes Test
##
## Allometry Model
```

```
summary(wing.allometry.by.group)
```

```
##
## Call:
## procD.allometry(f1 = coords ~ Csize, f2 = ~morph * ecotype *
##     sex, logsz = TRUE, iter = i, RRPP = TRUE, print.progress = F,
##     data = gdf)
##
##
##
## Homogeneity of Slopes Test
##                     Df     SSE       SS       R2      F      Z Pr(>F)
## Common Allometry  157 0.71142
## Group Allometries 150 0.62278 0.088638 0.025569 3.0498 12.213  1e-04 ***
## ---
## Signif. codes:  0 '***' 0.001 '**' 0.01 '*' 0.05 '.' 0.1 ' ' 1
##
## The null hypothesis of parallel slopes is rejected
##   based on a significance criterion of alpha = 0.05
##
## Based on the results of this test, the following ANOVA table is most appropriate
##
## Type I (Sequential) Sums of Squares and Cross-products
## Randomized Residual Permutation Procedure Used
## 10000 Permutations
## ANOVA effect sizes and P-values based on empirical F distributions
##
##
##                              Df     SS      MS     Rsq        F      Z
## log(size)                     1 2.4081 2.40811 0.69467 580.0049 6.5344
## morph                         1 0.1607 0.16072 0.04636  38.7104 7.9807
## ecotype                       1 0.0138 0.01385 0.00399   3.3351 4.6830
## sex                           1 0.1508 0.15082 0.04351  36.3267 8.6432
## morph:ecotype                 1 0.0039 0.00386 0.00111   0.9292 3.1358
## morph:sex                     1 0.0110 0.01099 0.00317   2.6480 5.1725
## ecotype:sex                   1 0.0049 0.00493 0.00142   1.1867 3.6564
## log(size):morph               1 0.0572 0.05724 0.01651  13.7855 8.3250
## log(size):ecotype             1 0.0122 0.01224 0.00353   2.9490 5.7193
## log(size):sex                 1 0.0057 0.00573 0.00165   1.3789 4.2697
## morph:ecotype:sex             1 0.0019 0.00190 0.00055   0.4585 2.1380
## log(size):morph:ecotype       1 0.0042 0.00420 0.00121   1.0110 3.5532
## log(size):morph:sex           1 0.0022 0.00219 0.00063   0.5277 2.3951
## log(size):ecotype:sex         1 0.0031 0.00312 0.00090   0.7515 3.1037
## log(size):morph:ecotype:sex   1 0.0039 0.00386 0.00111   0.9305 3.4597
```

```
## Residuals                      150 0.6228 0.00415
## Total                          165 3.4665
##                                Pr(>F)
## log(size)                       1e-04 ***
## morph                           1e-04 ***
## ecotype                         1e-04 ***
## sex                             1e-04 ***
## morph:ecotype                  0.0008 ***
## morph:sex                       1e-04 ***
## ecotype:sex                    0.0002 ***
## log(size):morph                 1e-04 ***
## log(size):ecotype               1e-04 ***
## log(size):sex                   1e-04 ***
## morph:ecotype:sex              0.0179 *
## log(size):morph:ecotype         1e-04 ***
## log(size):morph:sex            0.0100 *
## log(size):ecotype:sex          0.0013 **
## log(size):morph:ecotype:sex 0.0003 ***
## Residuals
## Total
## ---
## Signif. codes:  0 '***' 0.001 '**' 0.01 '*' 0.05 '.' 0.1 ' ' 1
```

Significant interaction effects suggest allometric shape differences based on morph and ecotype. Wing shapes vary considerably with size among short-wing morphs (red and orange in the plot above), while long-wing morphs vary very little overall size (light and dark green).

Ontogenetic allometries

Growth of the forewing is strongly hyperallometric (Supplementary Figure 3). Wing primordia are pigmented at all stages, allowing measurements of ontogenetic allometry. As hemimetabolous insects, newly hatched soapberry bugs resemble adults in overall anatomy, but they lack adult pigmentation patterns, wings, and genitalia. Juvenile development proceeds through five instars, during which time wing growth is hyperallometric (Supplementary Figure 3).

The final adult wing length (and the wing morph) cannot be predicted by the fifth instar head size or length of the wing pad. Despite differences in adult wing length, the length of fifth instar wing pads does not appear to indicate a juvenile's future wing morph (lines in Supplementary Figure 3).

```
oa <- read.csv("Jhae.oa.csv", header=T)
```

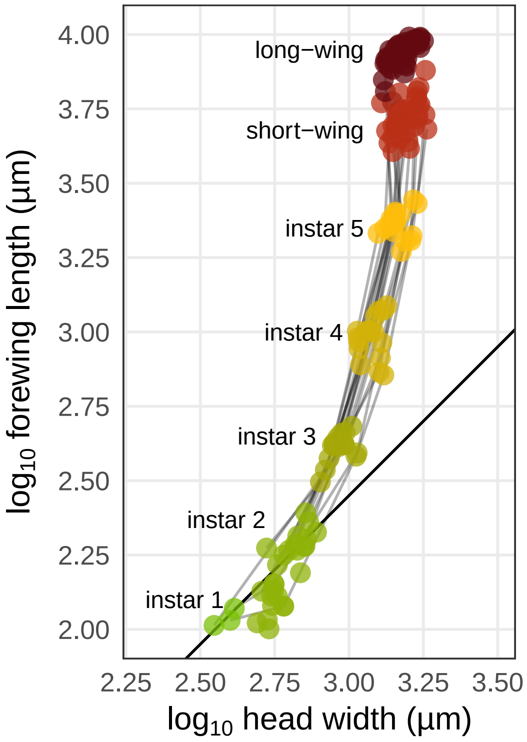

Supplementary Figure 3. A log-log plot of wing pad length in *J. haematoloma* versus head width, a proxy for overall body size, reveals hyperallometric growth of wing primordia. Proportional (isometric) growth is indicated by the black line. The plot includes 201 measurements, with trajectories for 78 individuals measured at multiple instars.

Ontogenetic scaling coefficients

Scaling coefficients are the slope of the regression line for specimens in a log-log plot of structure size to overall size[6]. We have used head width as a proxy for overall body size, since the bug abdomen can expand and contract with feeding, water retention and growth within an instar. The scaling coefficient for ontogenetic allometry can be calculated over any developmental interval.

Stage means will be used here to eliminate the bias introduced by varying sample numbers from each instar. We also average all adults.

```
stage.means <- data.frame(row.names=stage.names[1:5])
for (i in 1:5) {
  stage.means[i,1] <- mean(na.omit(oa$daid[which(oa$stage==i)]))
  stage.means[i,2] <- mean(na.omit(oa$winglength[which(oa$stage==i)]))
}
colnames(stage.means) <- colnames(oa)[4:5]

stage.means[6,1] <- mean(na.omit(oa$daid[which(oa$stage>5)]))
stage.means[6,2] <- mean(na.omit(oa$winglength[which(oa$stage>5)]))
rownames(stage.means)[6] <- "adult"

ontogenetic.scaling <- vector()
for (i in 1:5) {
  ontogenetic.scaling[i] <- with(stage.means[c(i,i+1),], lm(log10(winglength) ~ log10(daid)))$coefficie
  names(ontogenetic.scaling)[i] <- paste(rownames(stage.means)[i],"to",rownames(stage.means)[i+1],sep="
}
```

```
ontogenetic.scaling[6] <- ontogenetic.scaling[5]
ontogenetic.scaling[5] <- with(stage.means[3:6,],
                              lm(log10(winglength) ~ log10(daid)))$coefficients[2]
ontogenetic.scaling[7] <- with(stage.means,
                              lm(log10(winglength) ~ log10(daid)))$coefficients[2]
names(ontogenetic.scaling)[5:7] <- c("instar 3 to adult","instar 5 to adult","overall")
ontogenetic.scaling <- as.data.frame(ontogenetic.scaling)
colnames(ontogenetic.scaling) <- c("scaling coefficient")
knitr::kable(ontogenetic.scaling, digits = 3)
```

	scaling coefficient
instar 1 to instar 2	0.911
instar 2 to instar 3	2.236
instar 3 to instar 4	3.446
instar 4 to instar 5	4.298
instar 3 to adult	5.174
instar 5 to adult	23.589
overall	2.794

Supplementary Table 1. Scaling coefficients of each molt in *J. haematoloma*. Values represent the slope between each instar in Supplementary Figure 3.

Ontogenetic scaling coefficients were greater than 1 at each molt after the first (Supplementary Table 1). The primordia are external from the third instar to adulthood, with a scaling coefficient of 5.1 over this period, averaging both adult wing morphs.

Morph frequencies in the wild

Soapberry bugs are native to the coast of the Gulf of Mexico and Caribbean Islands, where they live on plants of the soapberry family (Supplementary Figure 4)[7,8]. *Jadera haematoloma* are found on *Cardiospermum corindum* in the Florida Keys and Everglades, and farther north in peninsular Florida on *Cardiospermum microcarpum*. In coastal Louisiana, Texas and Mexico, soapberry bugs are known to exploit another herbaceous sapind, *Serjania brachycarpa*. In sheltered interior river valleys, *J. haematoloma* can be found on the native soapberry tree, *Sapindus saponaria drummondii*.

After 1950, *J. haematoloma* made a host shift to introduced ornamental trees, *Koelreuteria* sp.. The most cold-tolerant and widely planted of these trees is *K. paniculata*, which can survive into New England and the Pacific Northwest. *Koelreuteria bipinnata* is less commonly planted and more limited in its hardiness. *Koelreuteria elegans* is limited to Florida, the Gulf coast, and parts of California and Arizona. All *Koelreuteria* sp. are known to support soapberry bug populations. However, any specialization among these hosts has not been explored.

While *Koelreuteria* sp. are in broad cultivation throughout the United States, *J. haematoloma* has not yet colonized all areas occupied by these host plants. It is unclear whether active planting by humans has allowed *Koelreuteria* to expand faster than the soapberry bugs, or whether *J. haematoloma* is limited by winter temperatures or low humidity in northern and dry interior regions.

We used permutation-based ANOVA and pairwise post hoc permutation tests to examine the relationship of wing morph frequencies with host plants. The frequency of wing morphs in the wild was significantly associated with their host plant species at that location (Supplementary Figure 4).

```
wild.bugs <- read.csv("Jhae.wild.ratios.csv", header=TRUE)
wild.bugs <- dplyr::filter(wild.bugs, census>3 & !is.na(pSW))
```

```r
summary(with(wild.bugs[x,], aovp(pSW ~ hostplant)))
```

```
## [1] "Settings:  unique SS "

## Component 1 :
##             Df R Sum Sq R Mean Sq Iter  Pr(Prob)
## hostplant    2   2.2629   1.13145 5000 < 2.2e-16 ***
## Residuals   57   3.3486   0.05875
## ---
## Signif. codes:  0 '***' 0.001 '**' 0.01 '*' 0.05 '.' 0.1 ' ' 1
```

```r
with(wild.bugs[x,], pairwise.perm.test(pSW,hostplant))
```

```
## Pairwise permutation tests
## Levels: Cardiospermum corindum Koelreuteria elegans Koelreuteria paniculata
## Multiple test correction using the  bonferroni  method
##
## Pairwise p-values
##                        v. Koelreut v. Koelreut
## Cardiospermum corindum     0.20619  0.00133440
## Koelreuteria elegans            NA  0.00008121

## $p.values
##                        v. Koelreut v. Koelreut
## Cardiospermum corindum     0.20619  0.00133440
## Koelreuteria elegans            NA  0.00008121
##
## $results
##                        v. Koelreut       p v. Koelreut          p
## Cardiospermum corindum      1.8202 0.20619    -3.51199 0.00133440
## Koelreuteria elegans                    NA    -4.19679 0.00008121
##
## $methods
##                        v. Koelreut Stat             Method
## Cardiospermum corindum               Z Asymptotic Approx.
## Koelreuteria elegans
##                        v. Koelreut Stat             Method
## Cardiospermum corindum               Z Asymptotic Approx.
## Koelreuteria elegans                 Z Asymptotic Approx.
##
## $p.adj
## [1] "bonferroni"
##
## $comp
##  Cardiospermum corindum    Koelreuteria elegans Koelreuteria paniculata
##                     "a"                     "a"                     "b"
##
## $time
## Time difference of 0.1262341 secs
```

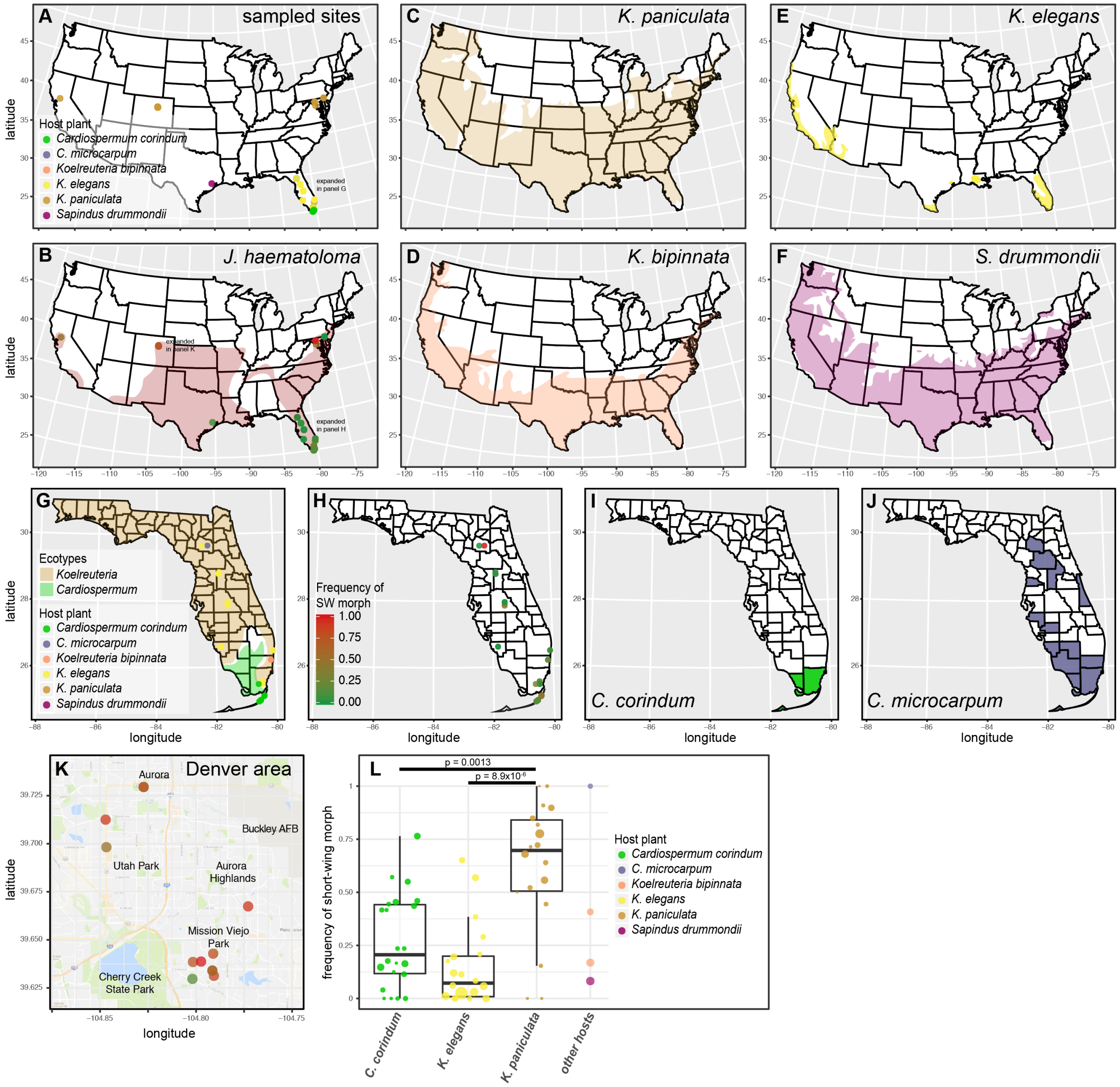

Supplementary Figure 4. Range maps for *Jadera haematoloma* and its host plants. Maps generated in R and combined in *Adobe Illustrator*, where shading for host ranges was added based on information from the Florida Cooperative Extension Service[9–12]. **(a)** Sites sampled in this study, color coded by host plant species. **(b)** The current known range of *Jadera haematoloma*, based on the review by 8, data presented here, and citizen science contributions to iNaturalist.org[13]. Points indicate sites sampled in this study, colored to represent the frequency of wing morphs (red for short wing; green for long wing). **(c-f)** The ranges of *K. paniculata*, *K. bipinnata*, *K. elegans*, and *S. drummondii*. **(g)** In Florida, *J. haematoloma* persist on the ancestral host plant, *Cardiospermum corindum*, shaded in light green, while most of the state (the rest of the country) have the *Koelreuteria* ecotype of *J. haematoloma* (shaded in light red). Collection sites in Florida are color coded by host plant species. **(h)** Florida collection sites colored by the frequency of wing morphs, as in panel B. **(i-j)** Records of *Cardiospermum corindum* and *C. microcarpum* are indicated in the shaded counties. **(k)** Multiple sites were sampled in the area of Aurora, Colorado. Points are colored to represent the variation in the frequency of wing morphs as in panel B. **(l)** Boxplot summarizing the frequencies of wing morphs on different host plants. Significant

differences exist in these frequencies for the three most heavily sampled hosts. p-values produced by permutation.

Modeling determination of morph frequencies

No evidence for Mendelian determination of wing morphs

We first tested whether the occurrence of long- and short-wing morphs was consistent with the expectations of Mendelian inheritance.

```
# Import data from single-pair crosses
crosses <- read.csv("Jhae.crosses.csv", header=T)
# Filter only data from crosses that scored F1 adult morphs
xf1 <- dplyr::filter(crosses, !is.na(F1.LWf))
# Add up the number of males, females, long-wing and short-wing bugs
xf1$F1.LW <- xf1$F1.LWf + xf1$F1.LWm
xf1$F1.SW <- xf1$F1.SWf + xf1$F1.SWm
xf1$F1.females <- xf1$F1.LWf + xf1$F1.SWf
xf1$F1.males <- xf1$F1.LWm + xf1$F1.SWm
xf1$F1.census <- xf1$F1.LW + xf1$F1.SW
xf1$mfratio <- xf1$F1.males / xf1$F1.females
xf1$mfratio[is.infinite(xf1$mfratio)] <- NA
xf1$F1.pSW <- xf1$F1.SW / xf1$F1.census
xf1 <- xf1[with(xf1, order(crosstype, F1.pSW)),]
```

We scored the morphs of adult offspring in the F_1 generation produced by 32 crosses of virgin adults of all possible morph combinations. Using Fisher's exact test, the number of observed long- and short-winged offspring was compared to expected numbers from several Mendelian ratios. Each cross was tested for consistency with all long- or short-wing offspring, 1:1, 3:1 either way, and the possibility of a 2:1 ratio due to a recessive lethal short-wing allele. After the tests of Mendelian ratios, crosses were examined for consistency with certain modes of Mendelian inheritance, including complete dominance for either long- or short-wing morphs, or the possibility of recessive lethal short-wing inheritance (Supplementary Table 2).

```
alpha.p <- 0.05
for (i in 1:dim(xf1)[1]) {
  # Test whether morph numbers fit predictions of Mendelian ratios
  xf1$p.MF[i] <- with(xf1, fisher.test(matrix(c(F1.females[i], F1.males[i],
                                             rep(F1.census[i]/2,2)),
                                           nrow=2)))$p.value
  xf1$p.SW0[i] <- with(xf1, fisher.test(matrix(c(F1.SW[i], F1.LW[i],
                                              0,F1.census[i]),
                                           nrow=2)))$p.value
  xf1$p.SW25[i] <- with(xf1, fisher.test(matrix(c(F1.SW[i], F1.LW[i],
                                              F1.census[i]*0.25,F1.census[i]*0.75),
                                            nrow=2)))$p.value
  xf1$p.SW50[i] <- with(xf1, fisher.test(matrix(c(F1.SW[i], F1.LW[i],
                                               F1.census[i]*0.5,F1.census[i]*0.5),
                                             nrow=2)))$p.value
  xf1$p.SW75[i] <- with(xf1, fisher.test(matrix(c(F1.SW[i], F1.LW[i],
                                               F1.census[i]*0.75,F1.census[i]*0.25),
                                             nrow=2)))$p.value
  xf1$p.SW67[i] <- with(xf1, fisher.test(matrix(c(F1.SW[i], F1.LW[i],
                                               F1.census[i]*(2/3),F1.census[i]*(1/3)),
                                             nrow=2)))$p.value
```

```r
  xf1$p.SW100[i] <- with(xf1, fisher.test(matrix(c(F1.SW[i], F1.LW[i],
                                                     F1.census[i],0),
                                                   nrow=2)))$p.value
  # Adjust p-values for multiple test
  xf1[i,25:29] <- p.adjust(xf1[i,25:29], method = "bonferroni", n=6)
  # Are adjusted p-values above the critical value?
  xf1$is.SW0[i] <- xf1$p.SW0[i] > alpha.p
  xf1$is.SW25[i] <- xf1$p.SW25[i] > alpha.p
  xf1$is.SW50[i] <- xf1$p.SW50[i] > alpha.p
  xf1$is.SW75[i] <- xf1$p.SW75[i] > alpha.p
  xf1$is.SW67[i] <- xf1$p.SW67[i] > alpha.p
  xf1$is.SW100[i] <- xf1$p.SW100[i] > alpha.p
  # For each type of cross, are the results consistent with expectations of
  # different scenarios of Mendelian inheritance?
  if (xf1$crosstype=="LL") {
    xf1$LWdominant[i] <- xf1$is.SW0[i] | xf1$is.SW25[i]
    xf1$SWdominant[i] <- xf1$is.SW0[i]
    xf1$SWlethal[i] <- xf1$is.SW0[i]
  } else
    if (xf1$crosstype=="SS") {
      xf1$LWdominant[i] <- xf1$is.SW100[i]
      xf1$SWdominant[i] <- xf1$is.SW100[i] | xf1$is.SW75[i]
      xf1$SWlethal[i] <- xf1$is.SW67[i]
    } else {
      xf1$LWdominant[i] <- xf1$is.SW0[i] | xf1$is.SW50[i]
      xf1$SWdominant[i] <- xf1$is.SW100[i] | xf1$is.SW50[i]
      xf1$SWlethal[i] <- xf1$is.SW50[i]
    }
}
# Extract the most important results to a new table for display
xf1.results <- xf1[,c(2,21,23,25:30,37:39)]
xf1.results$crosstype <- plyr::revalue(xf1$crosstype, c("LL"="LW x LW","LS"="LW x SW",
                                                        "SL"="SW x LW","SS"="SW x SW"))
xf1.results[,2:8] <- signif(xf1.results[,2:8],3)
colnames(xf1.results) <- c("Cross type","n","freq. F~1~ SW","0% SW","25% SW",
                           "50% SW","67% SW","75% SW","100% SW",
                           "Consistent with dominant LW", "dominant SW", "recessive lethal SW")
knitr::kable(xf1.results[,c(1:3,10:12)], row.names = FALSE, digits = 4)
```

Cross type	n	freq. F_1 SW	Consistent with dominant LW	dominant SW	recessive lethal SW
LW x LW	22	0.0000	TRUE	TRUE	TRUE
LW x LW	41	0.2680	TRUE	FALSE	FALSE
LW x LW	7	0.2860	TRUE	TRUE	TRUE
LW x LW	8	0.3750	TRUE	TRUE	TRUE
LW x LW	37	0.5140	TRUE	FALSE	FALSE
LW x LW	29	0.5520	TRUE	FALSE	FALSE
LW x LW	15	0.6000	TRUE	FALSE	FALSE
LW x LW	44	0.7050	FALSE	FALSE	FALSE
LW x SW	32	0.0312	TRUE	TRUE	TRUE
LW x SW	14	0.3570	TRUE	TRUE	TRUE
LW x SW	15	0.4670	TRUE	FALSE	FALSE
LW x SW	35	0.7430	FALSE	FALSE	FALSE
LW x SW	2	1.0000	TRUE	TRUE	TRUE

Cross type	n	freq. F_1 SW	Consistent with dominant LW	dominant SW	recessive lethal SW
LW x SW	6	1.0000	TRUE	FALSE	FALSE
SW x LW	18	0.6110	TRUE	FALSE	FALSE
SW x LW	8	0.6250	TRUE	TRUE	TRUE
SW x LW	35	0.6290	FALSE	FALSE	FALSE
SW x LW	19	0.7890	FALSE	FALSE	FALSE
SW x LW	34	0.8530	FALSE	FALSE	FALSE
SW x LW	1	1.0000	TRUE	TRUE	TRUE
SW x LW	1	1.0000	TRUE	TRUE	TRUE
SW x LW	5	1.0000	TRUE	FALSE	FALSE
SW x LW	2	1.0000	TRUE	TRUE	TRUE
SW x SW	21	0.3810	TRUE	FALSE	FALSE
SW x SW	5	0.4000	TRUE	TRUE	TRUE
SW x SW	20	0.6000	TRUE	FALSE	FALSE
SW x SW	46	0.6960	FALSE	FALSE	FALSE
SW x SW	43	0.7670	FALSE	FALSE	FALSE
SW x SW	10	0.8000	TRUE	FALSE	FALSE
SW x SW	5	0.8000	TRUE	TRUE	TRUE
SW x SW	19	1.0000	FALSE	FALSE	FALSE
SW x SW	2	1.0000	TRUE	TRUE	TRUE

Supplementary Table 2. Controlled crosses were used to test hypotheses of Mendelian inheritance for wing morphs. Fisher's exact test compared the results of each cross to six different possible phenotypic ratios for the short- vs. long-morph (0, 1:3, 1:1, 2:1, 3:1, 1). The resulting *p*-values were adjusted for multiple tests using the Bonferroni method. For each cross-type, it was determined based on the adjusted *p*-values, whether the cross results were consistent with dominance of the long-wing (LW) state, dominance of the short-wing (SW) state, or a recessive lethal short-wing allele.

None of the hypotheses of Mendelian inheritance is consistently supported by these data. Of the 32 crosses, 8 are inconsistent with inheritance of the long-wing morph by a dominant allele, 19 crosses are inconsistent with dominance of the short-wing morph and 19 with the hypothesis of a recessive lethal short-wing allele. Since genetic factors do not obviously explain the occurrence of wing morphs, we tested the influence of environmental factors.

Polyphenic reaction norms of each ecotype

Insect polyphenisms are typically mediated by one or more factors, including juvenile nutrition, density, photoperiod or temperature. Each of these can be reliable cues to facilitate dispersal/fecundity trade-offs[14,15]. Previous work has suggested that *J. haematoloma* morphs are determined by juvenile nutrition, with a positive correlation between food level and frequency of the short-winged morph[16]. In contrast, no evidence for the influence of temperature or photoperiod exists[16].

In order to better understand the cues that specify each morph in *J. haematoloma*, we raised cohorts under a wide range of food and individual density conditions in the lab, varying the number of seeds available and the size of cohorts. The surviving adults from each of these treatments were then scored for morph and sex. Comparison of generalized linear models supports the influence of both factors, their interactions, and the bugs' population of origin. Regression of wing morph frequencies to these factors revealed that increasing food availability and lower conspecific competition (smaller cohorts) were correlated with increased frequencies of the short-wing morph (Figure 2a).

To examine the determination of morphs, soapberry bugs were collected from lab cultures as first or second instars. Precise numbers of bugs and seeds of their natal host plant were combined in a 3.38-L terrarium. Bugs were incubated at 26°C until all juveniles molted to adulthood or died. Water was replaced daily, but

no food was removed or added. Dead bugs were removed periodically. Adults were then scored for sex and morph.

```
rxn.all.data <- read.csv("reaction.norms.csv", header=TRUE, stringsAsFactors = FALSE)
# Subset data for cohorts not used in RNAi or cross-rearing experiments
rxn <- dplyr::filter(rxn.all.data, treatment=="unmanip" &
                                as.character(ecotype)==as.character(seedspecies))
# Create a version including the F1 data
rxn.with.f1 <- rbind(rxn, dplyr::filter(rxn.all.data, pop=="F1"))

# Subsets for each ecotype alone
bv <- rxn[which(rxn$ecotype=="BV"),]
grt <- rxn[which(rxn$ecotype=="GRT"),]
```

The response of *J. haematoloma* cohorts to the environmental conditions of seed number and cohort size can be represented as reaction norms for each population.

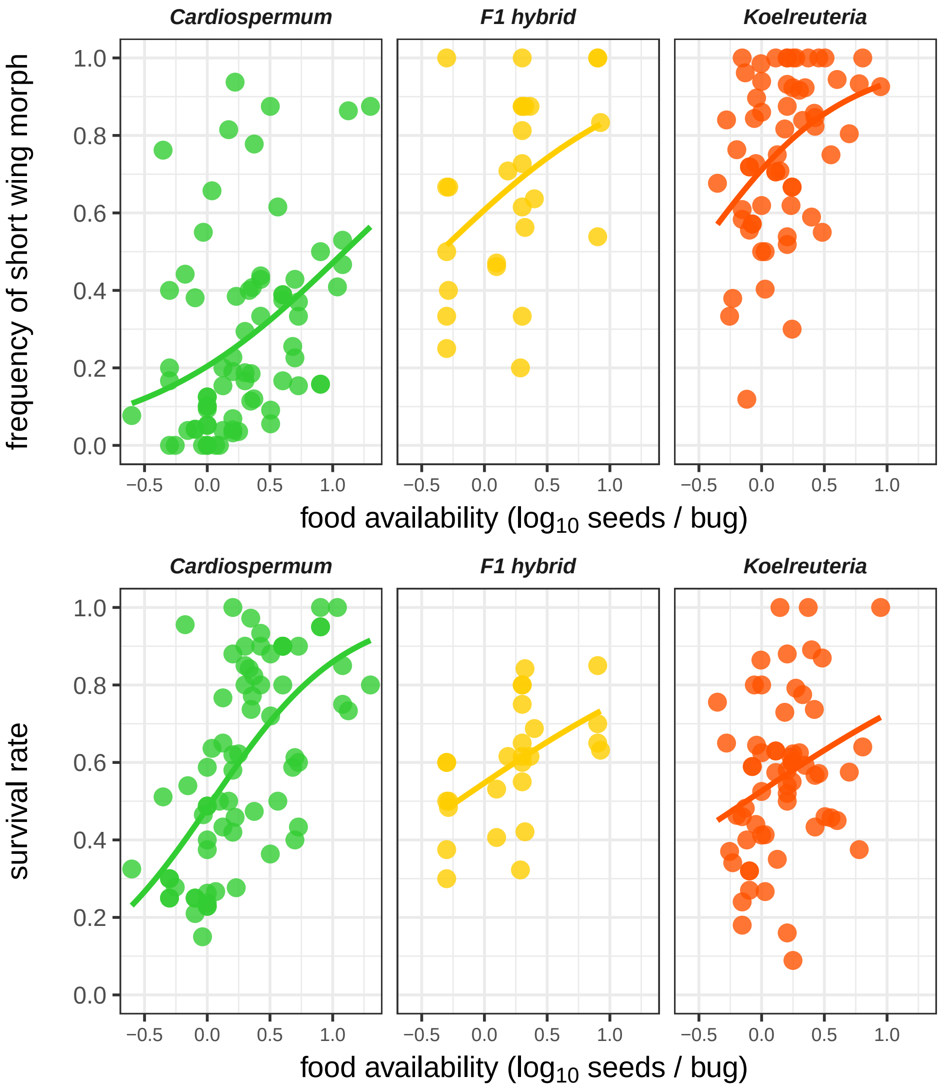

These plots were adjusted in *Adobe Illustrator* and appear in the main text as Figure 2.

Logistic regression

Here short- or long-winged morphs are modeled as two binary outcomes for the individuals in a cohort. Logistic regression was used to predict the frequency of wing morphs in lab-raised cohorts of the *Cardiospermum* and *Koelreuteria* ecotypes, in combination and separately. The combined dataset includes 3490 sampled adults from 133 treatments.

We define a function (`logistic.reg.analysis` in the code below) to compare a given number of models in logistic regression, recording the Akaike information criteria[17], χ^2, degrees of freedom and associated p-value, as well as the pseudo-R^2 of Hosmer & Lemeshow[18]. The function also retains coefficients and other results from the most favorable model. This method uses a generalized linear model with a logit link function (`glm(formula, family = binomial(link = "logit"))`). The code for this R function can be viewed in the accompanying script file, `logistic.regression.analysis.R`.

We considered all possible models combining the number of seeds provided as food (`seeds`), the number of conspecifics initially set-up in the cohort (`cohort`), and the bugs' ecotype (`ecotype`) or their population-of-origin (`pop`).

```
# Define models
models <- c("1","0","seeds","cohort","ecotype","seeds + cohort","seeds * cohort",
            "seeds + ecotype","seeds * ecotype","cohort + ecotype","cohort * ecotype",
            "seeds + cohort + ecotype","seeds * cohort + ecotype",
            "seeds + cohort * ecotype","seeds * cohort * ecotype","pop")

# Order populations by increasing latitude
# (PlantationKey and KeyLargo are Cardiospermum ecotypes.
#  Other populations are Koelreuteria ecotypes.)
rxn$pop <- factor(rxn$pop, levels = c("PlantationKey","KeyLargo",
                                      "Greenbrier","Frederick","Aurora"))
# Run the modeling function
lra <- logistic.reg.analysis(rxn, models)
# Display results
knitr::kable(lra$results.by.aic)
```

	AIC	x^2	df	p	$R^2{}_{\mathrm{H\&L}}$
seeds * cohort + ecotype	1131.658	956.30	4	0.0000000	0.5551
seeds * cohort * ecotype	1134.450	959.51	7	0.0000000	0.5570
seeds + cohort + ecotype	1140.835	945.13	3	0.0000000	0.5486
seeds + cohort * ecotype	1142.432	945.53	4	0.0000000	0.5489
cohort + ecotype	1203.244	880.72	2	0.0000000	0.5113
cohort * ecotype	1204.323	881.64	3	0.0000000	0.5118
seeds + ecotype	1259.699	824.26	2	0.0000000	0.4785
pop	1259.717	828.25	4	0.0000000	0.4808
seeds * ecotype	1260.671	825.29	3	0.0000000	0.4791
ecotype	1294.350	787.61	1	0.0000000	0.4572
seeds * cohort	2068.121	17.84	3	0.0004746	0.0104
seeds	2077.058	4.90	1	0.0268600	0.0028
seeds + cohort	2078.021	5.94	2	0.0513000	0.0034
1	2079.962	0.00	0	1.0000000	0.0000
0	2080.536	0.00	0	1.0000000	0.0000
cohort	2081.471	0.49	1	0.4839000	0.0003

Supplementary Table 3. Models tested in logistic regression for specification of wing morphs. Abbreviations: df, degrees of freedom; $R^2{}_{\mathrm{H\&L}}$, Hosmer & Lemeshow's correlation[18].

The fitness of models was compared using AIC. Of the 16 models considered, the favored model includes seed number, cohort size, their interaction, and ecotype as predictors of an individual bug's morph. For each predictor it is possible to find 95% confidence intervals on the odds ratio. We define a function to do so.

```
lra.or <- odds.confidence(lra)
knitr::kable(lra.or)
```

	B	std. err.	p		2.5% CI	odds ratio	97.5% CI
(Intercept)	-1.0300000	0.1610000	0.0000000	***	0.2599	0.3571	0.4886
seeds	0.0072770	0.0012050	0.0000000	***	1.0050	1.0070	1.0100
cohort	-0.0095840	0.0026220	0.0002565	***	0.9854	0.9905	0.9956
ecotypeGRT	2.4560000	0.0895700	0.0000000	***	9.7970	11.6600	13.9200
seeds:cohort	-0.0000582	0.0000177	0.0009910	***	0.9999	0.9999	1.0000

Supplementary Table 4. Coefficients (log odds; B) and odds ratios for the best model identified by AIC in logistic regression for specification of wing morphs among all specimens. 95% confidence intervals (CI) are given for the odds ratios.

While the number of seeds and conspecifics make significant contributions to the odds of a bug developing as short-winged, their effects are relatively small (1.007 and 0.9905, respectively). In contrast, the odds ratio based on ecotype (membership in the *Koelreuteria* ecotype) is 11.66.

```
rxn$predicted.prob <- fitted(lra$best.model)
rxn$res <- resid(lra$best.model)
binnedplot(rxn$predicted.prob,rxn$res, main="", cex.pts=1.2)
```

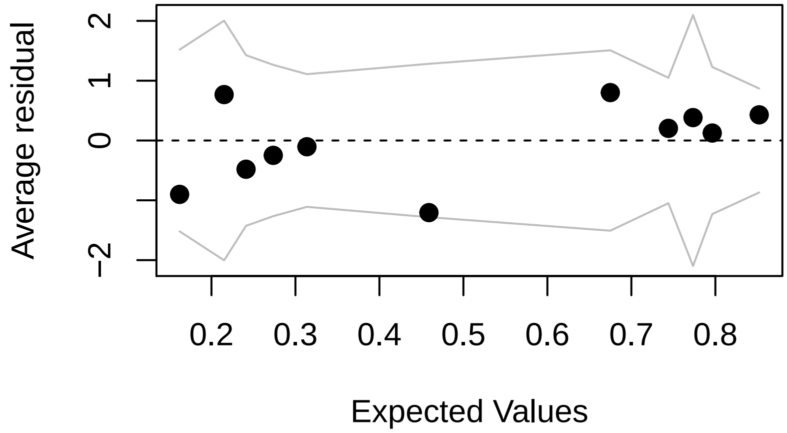

Supplementary Figure 5. The model fit can be judged from a binned residual plot[19]. Here, data are grouped by predicted values and average residuals are plotted with gray lines demarcating 2 standard errors. For a good model, 95% of residuals should fall within these bounds. The favored model, seeds * cohort + ecotype, accommodates the data well.

The *Cardiospermum* ecotype

Next, we wished to test whether the factors predicting morphs were the same within each ecotype.

```
models.within.ecotype <- c("1","0","seeds","cohort","seeds+cohort","seeds*cohort","pop")
lra.bv <- logistic.reg.analysis(bv, models.within.ecotype)
knitr::kable(lra.bv$results.by.aic)
```

	AIC	x^2	df	p	$R^2_{\text{H\&L}}$
seeds*cohort	614.4597	81.89	3	0.0000000	0.1642

	AIC	x^2	df	p	$R^2{}_{\text{H\&L}}$
seeds+cohort	621.7548	72.59	2	0.0000000	0.1456
seeds	659.9330	32.41	1	0.0000000	0.0650
cohort	664.2858	28.06	1	0.0000001	0.0563
pop	689.8552	2.49	1	0.1146000	0.0050
1	690.3458	0.00	0	1.0000000	0.0000
0	1047.7327	0.00	0	1.0000000	0.0000

Supplementary Table 5. Models tested in logistic regression for specification of wing morphs among *Cardiospermum* ecotypes. Abbreviations as in Supplementary Table 3.

```
knitr::kable(odds.confidence(lra.bv))
```

	B	std. err.	p		2.5% CI	odds ratio	97.5% CI
(Intercept)	-1.1490000	0.2114000	0.0000001	***	0.2086	0.3171	0.4781
seeds	0.0072280	0.0013570	0.0000001	***	1.0050	1.0070	1.0100
cohort	-0.0062960	0.0038340	0.1005000		0.9862	0.9937	1.0010
seeds:cohort	-0.0000633	0.0000211	0.0027380	**	0.9999	0.9999	1.0000

Supplementary Table 6. Coefficients and odds ratios for the best model identified by logistic regression for specification of wing morphs among *Cardiospermum* ecotypes.

The *Koelreuteria* ecotype

```
lra.grt <- logistic.reg.analysis(grt, models.within.ecotype)
knitr::kable(lra.grt$results.by.aic)
```

	AIC	x^2	df	p	$R^2{}_{\text{H\&L}}$
seeds*cohort	519.9899	90.01	3	0.0000	0.2063
seeds+cohort	522.4589	85.55	2	0.0000	0.1961
cohort	540.0371	65.97	1	0.0000	0.1512
pop	569.8615	38.14	2	0.0000	0.0874
seeds	600.7384	5.27	1	0.0217	0.0121
1	604.0042	0.00	0	1.0000	0.0000
0	1032.8030	0.00	0	1.0000	0.0000

Supplementary Table 7. Models tested in logistic regression for specification of wing morphs among *Koelreuteria* ecotypes. Abbreviations as in Supplementary Table 3.

```
knitr::kable(odds.confidence(lra.grt))
```

	B	std. err.	p		2.5% CI	odds ratio	97.5% CI
(Intercept)	1.3400000	0.2991000	0.0000075	***	2.1340	3.8170	6.8990
seeds	0.0097360	0.0029150	0.0008368	***	1.0040	1.0100	1.0160
cohort	-0.0095480	0.0042940	0.0261800	*	0.9821	0.9905	0.9988
seeds:cohort	-0.0000784	0.0000374	0.0363200	*	0.9998	0.9999	1.0000

Supplementary Table 8. Coefficients and odds ratios for the best model identified by logistic regression for specification of wing morphs among *Koelreuteria* ecotypes.

For each ecotype subset, the best model, according to AIC, combined the number of individuals and the

number of seeds, as well as their interaction.

Based on these results, seed number and cohort size were combined into a metric of food availability, which we defined as the $\log_{10}$ of seed number per bug (Figure 2).

Cross-rearing

In the previous analysis, we identified that ecotype (genetic background) was a significant factor in determining individual morphs. However, in those treatments, bugs of each ecotype were raised on seeds of the host plant associated with their population-of-origin. Therefore, we wanted to test for potential effects that seed species might have on morph determination by cross-rearing juveniles on non-natal seeds. We also produced F_1 ecotype hybrids, which were cross-reared on both species of seeds.

```
cross.reared.and.f1 <- dplyr::filter(rxn.all.data, treatment=="unmanip" &
                              as.character(ecotype)!="GFT" &
                              (seeds/cohort)>0.25 &
                              (seeds/cohort)<2.25)
grp <- as.factor(paste(cross.reared.and.f1$ecotype,cross.reared.and.f1$seedspecies,sep="/"))
```

```
## pdf
##   2
```

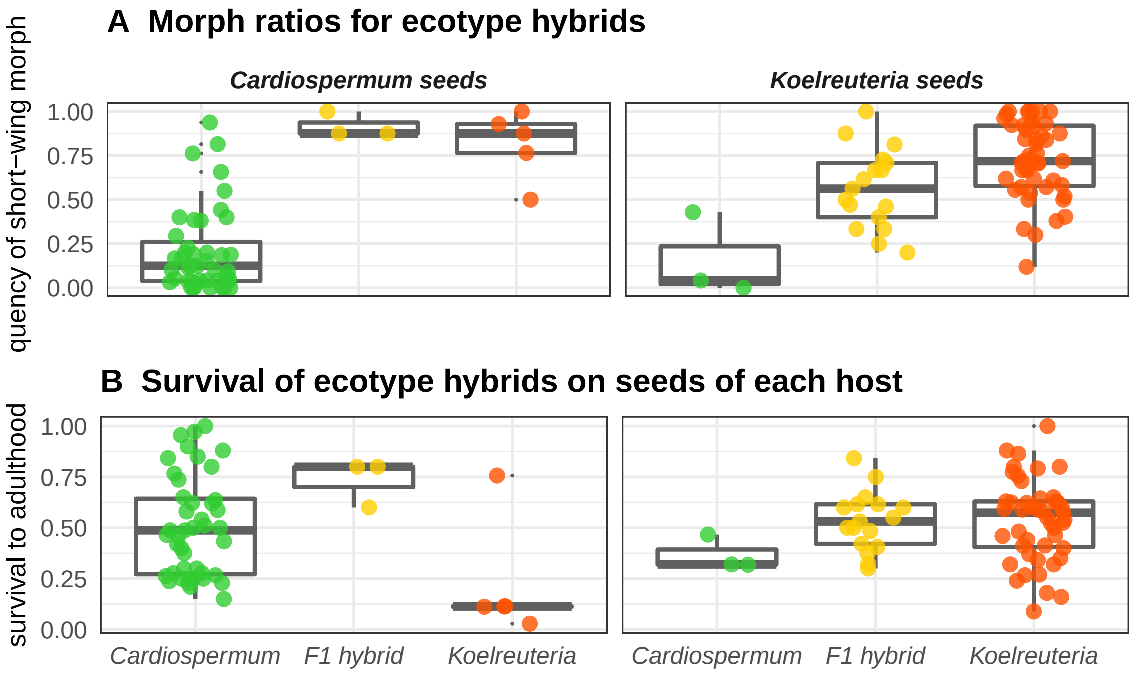

This plot was adjusted in *Adobe Illustrator* and the result was used as Figure 3.

Differences among cross-rearing groups and controls were tested using permutation. We began by testing for overall differences in factorial permutation ANOVA, considering ecotype and seed species.

```
cross.reared <- dplyr::filter(cross.reared.and.f1, ecotype!="F1")
summary(with(cross.reared, aovp(pSW ~ ecotype * seedspecies)))
```

```
## [1] "Settings:  unique SS "

## Component 1 :
##                          Df R Sum Sq R Mean Sq Iter Pr(Prob)
## ecotype1                  1   2.3985   2.39853 5000   <2e-16 ***
```

```
## seedspecies1           1   0.0322   0.03225   51   0.8235
## ecotype1:seedspecies1  1   0.0029   0.00285   51   0.6667
## Residuals             94   4.7728   0.05077
## ---
## Signif. codes:  0 '***' 0.001 '**' 0.01 '*' 0.05 '.' 0.1 ' ' 1
```

Given the significant effect of ecotype, we conduct a post-hoc two-sample permutation test.

```
with(cross.reared, permTS(pSW ~ ecotype))
```

```
##
##  Permutation Test using Asymptotic Approximation
##
## data:  pSW by ecotype
## Z = -7.5607, p-value = 4.009e-14
## alternative hypothesis: true mean ecotype=BV - mean ecotype=GRT is not equal to 0
## sample estimates:
## mean ecotype=BV - mean ecotype=GRT
##                          -0.5320363
```

```
summary(with(cross.reared, aovp(survival ~ ecotype * seedspecies)))
```

```
## [1] "Settings:  unique SS "
```

```
## Component 1 :
##                       Df R Sum Sq R Mean Sq Iter Pr(Prob)
## ecotype1               1   0.0220   0.02197  129   0.4419
## seedspecies1           1   0.0523   0.05234  148   0.4054
## ecotype1:seedspecies1  1   0.3463   0.34630 5000   0.0076 **
## Residuals             94   4.7501   0.05053
## ---
## Signif. codes:  0 '***' 0.001 '**' 0.01 '*' 0.05 '.' 0.1 ' ' 1
```

```
with(cross.reared[which(cross.reared$ecotype=="GRT"),], permTS(survival ~ seedspecies))
```

```
##
##  Permutation Test using Asymptotic Approximation
##
## data:  survival by seedspecies
## Z = -2.9012, p-value = 0.003717
## alternative hypothesis: true mean seedspecies=BV - mean seedspecies=GRT is not equal to 0
## sample estimates:
## mean seedspecies=BV - mean seedspecies=GRT
##                                 -0.3106274
```

For morph frequencies, ecotypes are significantly different within this range of food availability (Z = -7.56, p = 4.01x10^{-14}). However, the seed species alone does not appear to have a significant effect on morph frequencies ($p > 0.05$). Interestingly, survival was lower for bugs raised on non-natal host seeds. The reduction in survival was significant for *Koelreuteria*-ecotype bugs raised on *Cardiospermum* seeds (Z = -2.90, p = 0.00372).

Ecotype hybrids

Ecotype F_1 hybrids were created by crossing virgin adults from Frederick, MD and Tavernier, FL (Plantation Key). Bugs were isolated by sex during the fifth instar to insure that none had previously mated. Reciprocal mass crosses were then set up with 10 males and 10 females from each population of origin. The resulting F_1 eggs were removed prior to hatching. New hatchlings were removed to separate dishes to reduce cannibalism, as in our standard husbandry methods. Cohorts of 20 second instar F_1's were then used to establish treatments

under high or low food regimes with 160 or 40 seeds, respectively. Since host plant seeds are known to differ in nutritional value[20] replicate treatments were conducted with seeds of *Cardiospermum* and *Koelreuteria*. Potential maternal effects on wing morph frequencies may be revealed by differences among reciprocal crosses.

```
hybrids <- dplyr::filter(rxn.all.data, treatment=="unmanip" &
                         as.character(ecotype)=="F1")
# Extract from notes, the populatin of origin for the dams and sires from each cohort
hybrids$food <- hybrids$seeds/hybrids$cohort
for (i in 1:dim(hybrids)[1]) {
  n <- as.character(hybrids$note[i])
  n <- gsub("F1: ", "", n)
  n <- gsub(" x ", " ", n)
  hybrids$dampop[i] <- unlist(strsplit(n,"\\s"))[1]
  hybrids$sirepop[i] <- unlist(strsplit(n,"\\s"))[2]
}
# with(hybrids,by(dampop,dampop,length))   # AC: 2   FR: 5   PK: 19
# with(hybrids,by(sirepop,sirepop,length)) # AC: 7   FR: 12   PK: 7

# Permuation ANOVA
summary(with(hybrids, aovp(pSW ~ dampop + sirepop)))
```

```
## [1] "Settings:  unique SS "
```

```
## Component 1 :
##             Df  R Sum Sq R Mean Sq Iter Pr(Prob)
## dampop1      2  0.18725  0.093623  760   0.1684
## sirepop1     1  0.06970  0.069704  364   0.2170
## Residuals   22  1.28195  0.058271
```

The direction of the cross is not a significant factor for wing morph frequency. This suggests that maternal effects are not an influence on wing morphs.

Hybrid cohorts were raised on each of the two host plant species' seeds. These data provide another opportunity to examine the influence of the host plant species on morph determination.

```
with(hybrids, permTS(pSW ~ seedspecies))
```

```
##
##  Exact Permutation Test (network algorithm)
##
## data:  pSW by seedspecies
## p-value = 0.0009121
## alternative hypothesis: true mean seedspecies=BV - mean seedspecies=GRT is not equal to 0
## sample estimates:
## mean seedspecies=BV - mean seedspecies=GRT
##                                     0.3563667
```

The species of host plant seed is a significant factor. For these hybrids, *Cardiospermum* seeds produce more short-wing bugs than with *Koelreuteria* seeds. Interestingly, the direction of this effect is the opposite of the observed difference in the reaction norms of the each ecotype in which *Koelreuteria*-adapted bugs are more inclined to be short-winged, regardless of the nutritional environment. This suggests that in the same (F_1) genetic background, nutritional influences on wing morph determination may actually be maladaptive relative to wing morph-responses that may have been selected for over time within each host-adapted ecotype. If so, this maladaptive effect would present obstacles to the colonization of *Koelreuteria* by *Cardiospermum*-ecotype bugs and may also limit gene flow in that direction.

```
# Test for seed species effects on F1 survival
with(hybrids, permTS(survival ~ seedspecies))
```

```
##
##  Exact Permutation Test (network algorithm)
##
## data:  survival by seedspecies
## p-value = 0.005078
## alternative hypothesis: true mean seedspecies=BV - mean seedspecies=GRT is not equal to 0
## sample estimates:
## mean seedspecies=BV - mean seedspecies=GRT
##                                  0.1953616
```

```
# Compare the survival of F1's and cross-reared Koelreuteria ecotypes
# on Cardiospermum seeds
with(cross.reared.and.f1[which(cross.reared.and.f1$seedspecies=="BV"
                               & cross.reared.and.f1$ecotype!="BV"),],
     permTS(survival ~ ecotype))
```

```
##
##  Exact Permutation Test (network algorithm)
##
## data:  survival by ecotype
## p-value = 0.07143
## alternative hypothesis: true mean ecotype=F1 - mean ecotype=GRT is not equal to 0
## sample estimates:
## mean ecotype=F1 - mean ecotype=GRT
##                          0.5083333
```

```
# Compare the survival of F1's and cross-reared Cardiospermum ecotypes
# on Koelreuteria seeds
with(cross.reared.and.f1[which(cross.reared.and.f1$seedspecies=="GRT"
                               & cross.reared.and.f1$ecotype!="GRT"),],
     permTS(survival ~ ecotype))
```

```
##
##  Exact Permutation Test (network algorithm)
##
## data:  survival by ecotype
## p-value = 0.05789
## alternative hypothesis: true mean ecotype=BV - mean ecotype=F1 is not equal to 0
## sample estimates:
## mean ecotype=BV - mean ecotype=F1
##                        -0.1648131
```

Hybrid survival is significantly better on *Cardiospermum* seeds, compared to *Koelreuteria* (exact permutation $p = 0.00508$). While hybrid survival on each host plant is higher than for cross-reared parental ecotypes, the difference is not significant.

Modeling F_1 response to food availability

If we include F_1 ecotype hybrids among the population-of-origin (`population`) factor in our logistic models, we can test whether F_1 hybrids are significantly different in their responses from either parent population. First, we filter the complete dataset to include only F_1 hybrids of the two most commonly used distinct ecotype populations, Frederick, Maryland, and Plantation Key, Florida, and data from these parental populations. We also remove cohorts raised on food availability metrics more than 20% of the maximum for hybrids or less than 80% of the minimum for hybrids.

```r
f1 <- dplyr::filter(hybrids, (as.character(dampop)=="PK" | as.character(dampop)=="FR") &
                             (as.character(sirepop)=="PK" | as.character(sirepop)=="FR"))
f1.and.p <- dplyr::filter(rxn, as.character(pop)=="PlantationKey" |
                               as.character(pop)=="Frederick")
f1.and.p <- rbind(f1.and.p[,1:25],f1[,1:25])
f1.and.p$pop <- droplevels(f1.and.p$pop)
f1.and.p$foodavailability <- f1.and.p$seeds / f1.and.p$cohort
max.f1.food <- max(f1.and.p$foodavailability[which(f1.and.p$pop=="F1")])
min.f1.food <- min(f1.and.p$foodavailability[which(f1.and.p$pop=="F1")])
f1.and.p <- dplyr::filter(f1.and.p, (foodavailability < 1.2*max.f1.food) &
                                    (foodavailability > 0.8*min.f1.food))
```

For the purposes of testing similarity among F_1 hybrids and their parental populations we examine a model with one categorical factor, `pop`, with F_1, Frederick, and Plantation Key as its categories.

```r
lra.f1 <- logistic.reg.analysis(f1.and.p, "pop")
lra.f1.or <- odds.confidence(lra.f1)
knitr::kable(lra.f1.or)
```

	B	std. err.	*p*		2.5% CI	odds ratio	97.5% CI
(Intercept)	-1.051	0.06833	0	***	0.3052	0.3495	0.399
popFrederick	1.960	0.09261	0	***	5.9310	7.1020	8.527
popF1	1.639	0.15530	0	***	3.8100	5.1510	7.008

Supplementary Table 9. Coefficients (B, the log odds) and odds ratios comparing the wing morph ratios of F_1 hybrids to the parental populations, Frederick (*Koelreuteria* ecotype) and Plantation Key (*Cardiospermum* ecotype). Plantation Key acts as the baseline in this analysis.

Under the model, comparison of the F_1 hybrid response to that of Plantation Key bugs is significantly different (odds ratio = 5.15, p = 4.776x10^{-26}).

```r
wald.or.comparison(lra.f1.or, ref=2,comp=3)[3,8:9]
```

```
##       *z* (v.popFrederick)    *p*
## popF1                -1.775 0.0759
```

However, status as a Frederick bug does not predict significant difference compared to the F_1 hybrids (Wald test, z = -1.775, p = 0.0759).

Fecundity assessments

Fecundity was assessed for isolated, wild-caught *J. haematoloma* females and for individuals paired in crosses.

```r
fecund <- read.csv("Jhae.fecundity.csv", header=TRUE)
```

Wild-caught female fecundity

This dataset includes 57 adult females observed daily for egg production for up to 66 days. We could not control the age of wild-caught females. Therefore we kept females in isolation and determined their rate of egg-laying after capture, from the first day eggs were produced until the last day eggs were produced. (Several females eventually stopped laying eggs, but continued to live for several days or weeks.)

Non-parametric methods are used to test for differences, since fecundity is time-dependent and unlikely to fit a normal distribution. The Kruskal-Wallis test was used to identify overall differences in the data, followed by

Wilcoxon rank sum tests for specific comparisons. Bonferroni correction was used when data were included in multiple tests.

```
grp <- as.factor(paste(fecund$host,fecund$morph))
kruskal.test(fecund$rate ~ grp)
```

```
##
##  Kruskal-Wallis rank sum test
##
## data:  fecund$rate by grp
## Kruskal-Wallis chi-squared = 10.994, df = 3, p-value = 0.01176
```

```
with(fecund, wilcox.test(rate ~ ecotype))
```

```
##
##  Wilcoxon rank sum test with continuity correction
##
## data:  rate by ecotype
## W = 482.5, p-value = 0.2185
## alternative hypothesis: true location shift is not equal to 0
```

```
with(fecund[which(fecund$morph=="LW"),], wilcox.test(rate ~ ecotype))$p.value %>%
  p.adjust(method="bonferroni",n=3) %>% signif(4) %>% print() -> p.wild.lw
```

```
## [1] 0.01068
```

```
with(fecund[which(fecund$morph=="SW"),], wilcox.test(rate ~ ecotype))$p.value %>%
  p.adjust(method="bonferroni",n=3) %>% signif(4) %>% print()
```

```
## [1] 1
```

```
with(fecund[which(fecund$ecotype=="Cardiospermum"),], wilcox.test(rate ~ morph))$p.value %>%
  p.adjust(method="bonferroni",n=3) %>% signif(4) %>% print()
```

```
## [1] 1
```

```
with(fecund[which(fecund$ecotype=="Koelreuteria"),], wilcox.test(rate ~ morph))$p.value %>%
  p.adjust(method="bonferroni",n=3) %>% signif(4) %>% print() -> p.wild.grt
```

```
## [1] 0.004288
```

The rate of egg-laying was not different overall among females of each ecotype, however long-wing *Cardiospermum* females laid eggs significantly faster than *Koelreuteria* females (Wilcoxon rank sum test with Bonferroni correction, $p = 0.01068$). The rate of egg-laying differs significantly among wild-caught females by wing morph in the *Koelreuteria* ecotype ($p = 0.004288$), with faster egg production among short-winged bugs. Females from *C. corindum* host plants also show a similar difference in means, but the low sample size of short-winged females from this host plant reduce the power of this comparison.

Single-pair crosses

```
crosses <- read.csv("Jhae.crosses.csv", header=T)
# Subset the data by female ecotype
bv.x <- dplyr::filter(crosses, femaleecotype=="BV")
grt.x <- dplyr::filter(crosses, femaleecotype=="GRT")
```

Wing polyphenism in insects has classically been explained as allowing a dispersal-or-fecundity trade-off[15,21]. Because females typically invest a greater mass into gametes and offspring[22], this sex is likely to benefit most from polyphenism. This potential sexual conflict may explain why wing polyphenism is limited to females in

many insects[15]. Wing morph frequencies don't differ significantly by sex in *J. haematoloma*. Therefore, we wished to investigate whether males of each morph might differ in their reproductive success. The previous analysis focused only on females and did not control for the morph of males. Therefore, we set-up males and females of each morph in 124 individual crosses.

Egg production is not normally distributed. Therefore we used permutation-based ANOVA to explore the influence of morph and ecotype for each parent on total egg production. The model included consideration of a potential interaction among the parental morphs.

```
summary(aovp(eggs ~ femalemorph * malemorph + femaleecotype + maleecotype, data=crosses))
```

```
## [1] "Settings:  unique SS "

## Component 1 :
##                        Df R Sum Sq R Mean Sq Iter Pr(Prob)
## femalemorph             1     2187      2187   51  0.74510
## malemorph               1    59347     59347 4911  0.02016 *
## femalemorph:malemorph   1    14048     14048   85  0.54118
## femaleecotype           1    50043     50043 4260  0.02300 *
## maleecotype             1    44550     44550 4220  0.02322 *
## Residuals             118  1321191     11197
## ---
## Signif. codes:  0 '***' 0.001 '**' 0.01 '*' 0.05 '.' 0.1 ' ' 1
```

The most significant factor in total egg production was the morph of the male parent. To explore this influence further, we tested for differences based on parental morphs within each ecotype.

```
summary(aovp(eggs ~ femalemorph * malemorph, data=bv.x))
```

```
## [1] "Settings:  unique SS "

## Component 1 :
##                       Df R Sum Sq R Mean Sq Iter Pr(Prob)
## femalemorph            1    34743     34743  971  0.09372 .
## malemorph              1    52786     52786 3514  0.02789 *
## femalemorph:malemorph  1     3064      3064   51  0.86275
## Residuals             72   763656     10606
## ---
## Signif. codes:  0 '***' 0.001 '**' 0.01 '*' 0.05 '.' 0.1 ' ' 1
```

```
permTS(eggs ~ femalemorph, data=bv.x)$p.value %>%
  p.adjust(method="bonferroni",n=2) %>% signif(4) %>% print() -> p.x.bv.fmorph
```

```
## [1] 0.06877
```

```
permTS(eggs ~ malemorph, data=bv.x)$p.value %>%
  p.adjust(method="bonferroni",n=2) %>% signif(4) %>% print() -> p.x.bv.mmorph
```

```
## [1] 0.03998
```

```
bv.x.comparisons <- with(bv.x, pairwise.perm.test(eggs,crosstype))
```

```
## Pairwise permutation tests
## Levels: LL LS SL SS
## Multiple test correction using the  bonferroni  method
##
## Pairwise p-values
##      v. LS  v. SL    v. SS
## LL 0.36524 0.2922 0.030976
## LS      NA 1.0000 1.000000
```

```
## SL       NA      NA 0.620800
```

```r
summary(aovp(eggs ~ femalemorph * malemorph, data=grt.x)) # NS
```

```
## [1] "Settings:  unique SS "
## Component 1 :
##                         Df R Sum Sq R Mean Sq Iter Pr(Prob)
## femalemorph              1    27963     27963  776   0.1147
## malemorph                1      167       167   51   0.7843
## femalemorph:malemorph    1      300       300   51   0.8039
## Residuals               44   524100     11911
```

```r
permTS(eggs ~ femalemorph, data=grt.x)
```

```
##
##  Permutation Test using Asymptotic Approximation
##
## data:  eggs by femalemorph
## Z = -1.5536, p-value = 0.1203
## alternative hypothesis: true mean femalemorph=LW - mean femalemorph=SW is not equal to 0
## sample estimates:
## mean femalemorph=LW - mean femalemorph=SW
##                                 -59.88421
```

```
## pdf
##   2
```

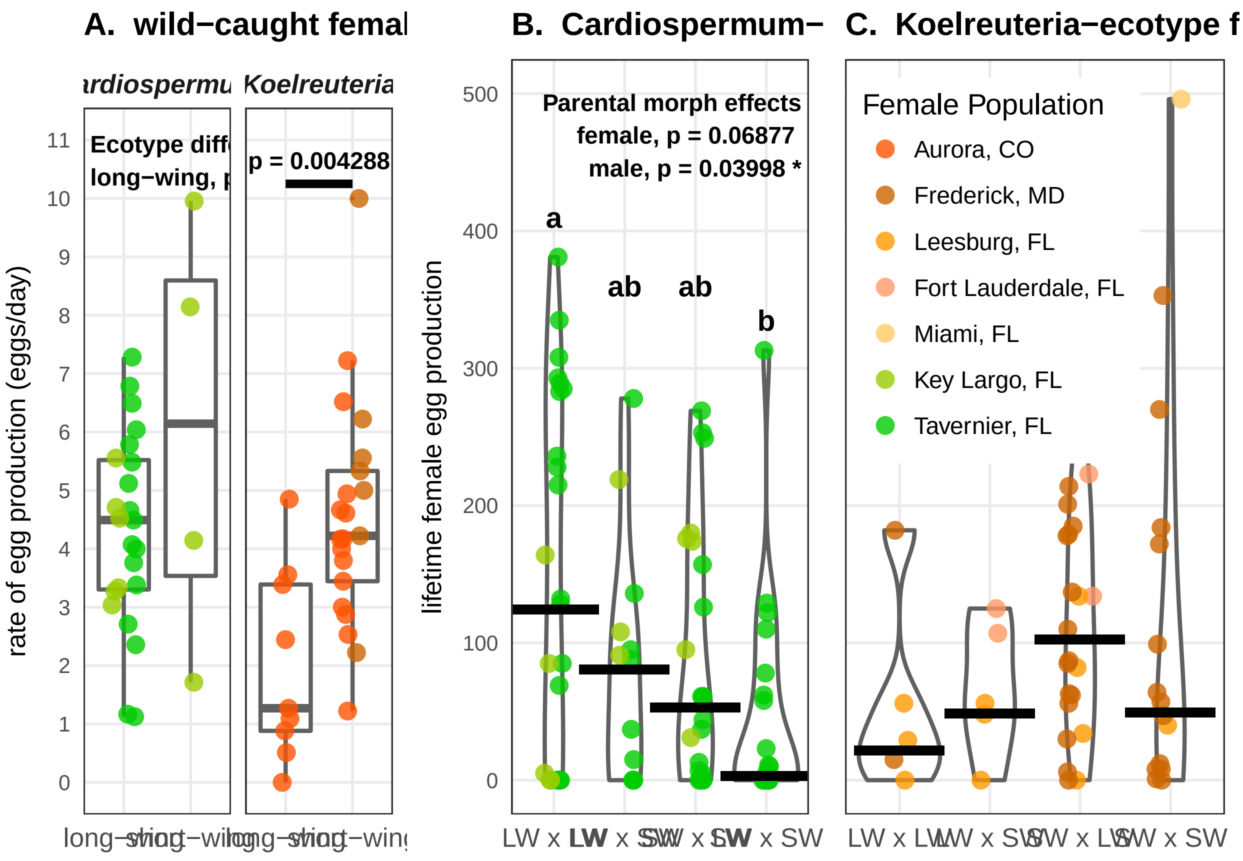

This plot appears in the main text as Figure 4.

Male morph had a significant effect on lifetime egg production for crosses. The effect is limited to crosses involving *Cardiospermum* ecotype females. There is no interaction among the morphs of the two sexes, so it appears to be an effect that is independent of the female's morph. These results imply that while wing polyphenism is adaptive in females, it may be maladaptive in males, at least for the *Cardiospermum* ecotype. Among *Koelreuteria* ecotype bugs, neither morph is a significant factor for egg production.

Examination of testes & accessory glands

In an attempt to identify the mechanism for reduced fecundity in short-winged *Cardiospermum*-ecotype males, we examined the relative sizes of the testes and accessory gland (AG) of males. There was no obvious visual difference in testes and AG by morph or ecotype. However, we compared the sizes of these organs, obtained by tracing outlines of images taken after dissection.

```
testes <- read.csv("Jhae.testes.AG.csv", header=T)
```

This dataset includes measurements from 58 testes and 32 accessory glands.

```
grp <- as.factor(with(testes, paste(organ,ecotype,morph,sep=".")))
te <- testes[which(testes$organ=="testes"),]
grp.te <- as.factor(with(testes[which(testes$organ=="testes"),], paste(organ,ecotype,morph,sep=".")))
ag <- testes[which(testes$organ=="AG"),]
grp.AG <- as.factor(with(testes[which(testes$organ=="AG"),], paste(organ,ecotype,morph,sep=".")))
```

```
with(testes, by(size,grp,length))
# AG samples are balanced, but there are 2-3 times more testes samples from
# Cardiospermum ecotype males than for Koelreuteria ecotypes.
with(testes, by(size,grp,shapiro.test))
# All groups are normally distributed
with(te, leveneTest(size,grp.te))
# Significant differences in testes size variation
with(ag, leveneTest(size,grp.AG))
# Similar variation
```

Sample sizes differ by ecotype-morph groups, but organ size measurements are all normally distributed. Significantly more variation exists in long-wing *Koelreuteria*-ecotype testes sizes. Given these violations of ANOVA assumptions, we made comparisons using nonparametric tests.

```
with(te, kruskal.test(size~grp.te))
```

```
##
##  Kruskal-Wallis rank sum test
##
## data:  size by grp.te
## Kruskal-Wallis chi-squared = 2.3711, df = 3, p-value = 0.499
```

```
with(ag, kruskal.test(size~grp.AG))
```

```
##
##  Kruskal-Wallis rank sum test
##
## data:  size by grp.AG
## Kruskal-Wallis chi-squared = 8.1596, df = 3, p-value = 0.04283
```

```
with(ag, wilcox.test(size~ecotype))
```

```
##
##  Wilcoxon rank sum test
```

```
##
## data:  size by ecotype
## W = 195, p-value = 0.01076
## alternative hypothesis: true location shift is not equal to 0
```

```r
with(ag, wilcox.test(size~morph))
```

```
##
##  Wilcoxon rank sum test
##
## data:  size by morph
## W = 155, p-value = 0.3126
## alternative hypothesis: true location shift is not equal to 0
```

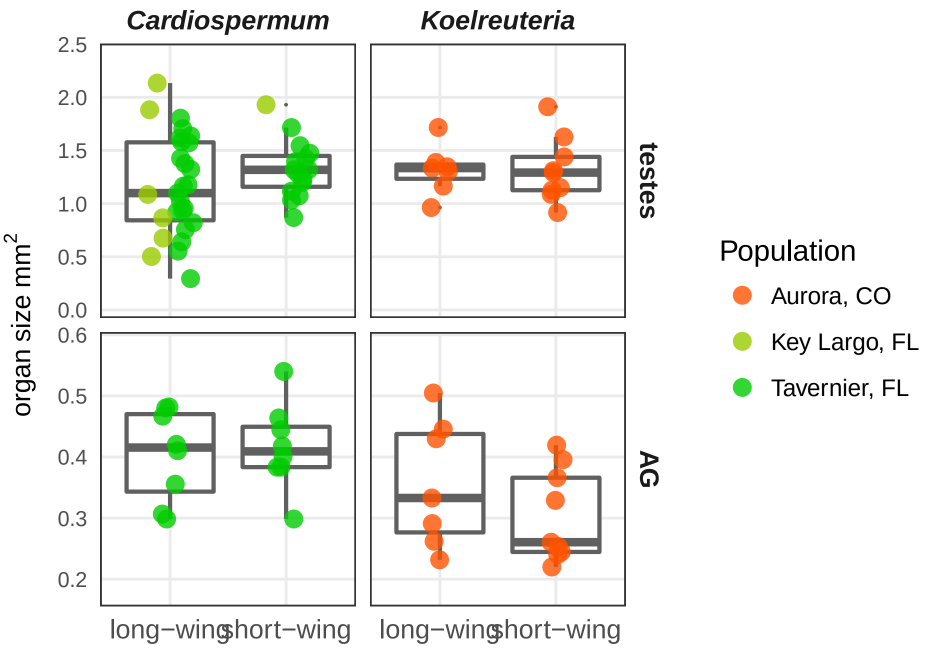

Supplementary Figure 6. Testes and accessory gland sizes in 58 nascent adult males, from different ecotypes and populations, as indicated by point color.

No significant differences exist for testes size by morph or ecotype. Interestingly, the size of the accessory gland differs significantly by ecotype, being larger in *Cardiospermum*-ecotype males. Comparison of ecotypes within each morph, also identified a significant difference in the size of the AG in short-wing males, which are larger in *Cardiospermum* ecotypes.

Since no differences were identified between morphs, overall or within each ecotype, the size of the reproductive organs does not help explain why short-wing *Cardiospermum*-ecotype males have reduced fertility.

Isolation of candidate genes

InR1 and *FoxO* were amplified by PCR using the primers listed in Supplementary Table 10.

target gene	primer name	sequence	direction	amplicon (bp)
InR1	Jh'InR-df5	TGTATCCACGATGTACGTACCAgaygantggga	forward	612
	Jh'InR-dr5	CCATCCTTCAGGGATTCAggnsncatcca	reverse	
	Jh'InR-df6	AACTGGGCCAAGGAACCttyggnayngt	forward	471

target gene	primer name	sequence	direction	amplicon (bp)
	Jh'InR-dr6	ccnctraarccGTACTGGGCTCTATAGATAC	reverse	
FoxO	Jh'FxO-df6	CGACTAACTCTGTCTCAAATTTACgartggatgrt	forward	273
	Jh'FxO-dr10a	AGCATTGGAAGAAGCTCGCkgnckraartc	reverse	
	Jh'foxo-36F	TCAGAATGTCACTTACTTCAAGG	forward	378
	Jh'FxO-dr10	GGAGGCTCGCGGCckraartcngg	reverse	

Supplementary Table 10. PCR primers used in the isolation of candidate genes.

Orthology assignments by phylogenetic inference

Most candidate genes had single orthologs in *J. haematoloma* that could be unambiguously identified using BLAST searches. However, because of the possibility of confusion among members of the insulin receptor family, we determined orthology for these genes using phylogenetic inference (Supplementary Figure 7).

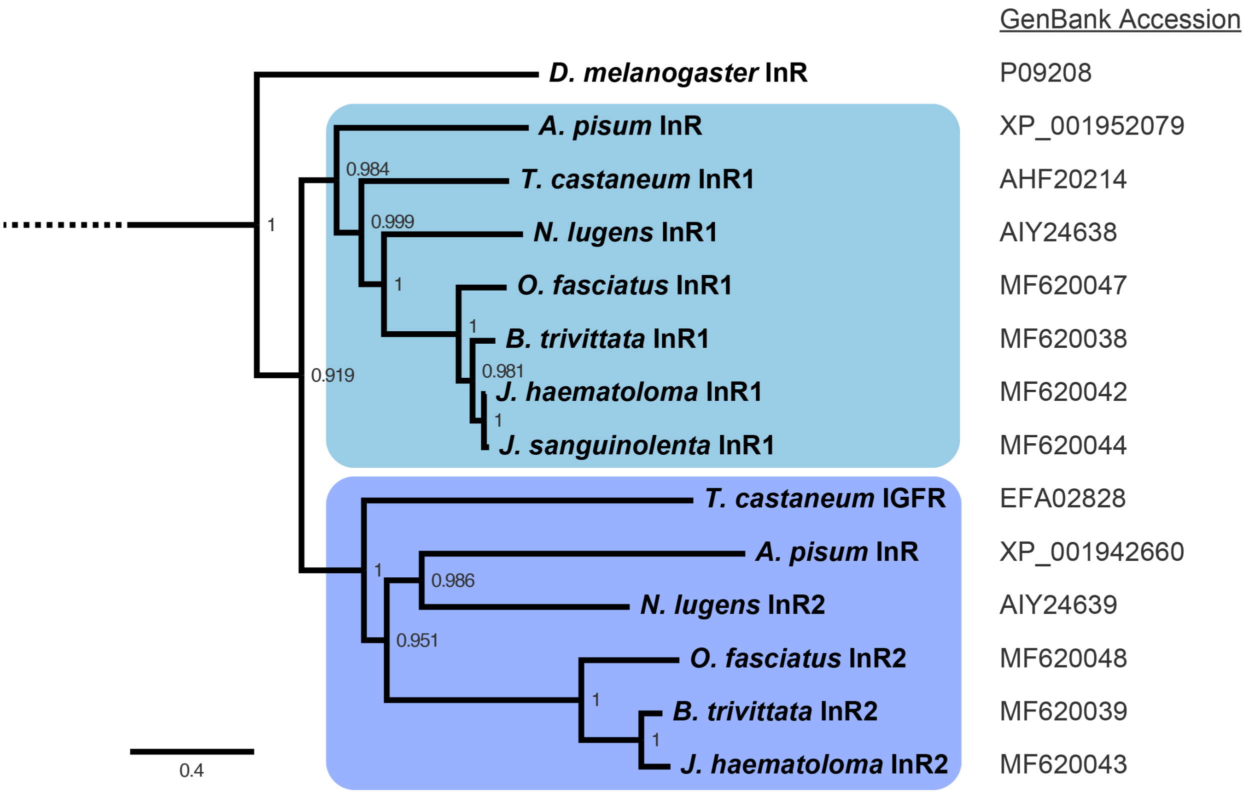

Supplementary Figure 7. Concensus phylogram based on amino acid sequences from 14 putative *InR* genes and 4 outgroup receptor tyrosine kinases strongly supports orthology of InR1 and InR2 proteins in most insects, denoted by the colored boxes. One exception is *Drosophila melanogaster*, which has a single InR protein without clear orthology to either receptor in other species. Nodes are labeled with Bayesian posterior support. Out-groups are omitted from the tree shown here. GenBank accession numbers are provided for reference. The consensus topology strongly supports two paralog groups.

Gene expression

Gene expression in nascent adult tissues

```
q <- read.csv("qPCR.data.csv", header=T) # read in the dataset
q$pop <- as.factor(strtrim(q$pop,2)) # remove collection year from population code
dt <- q[which(q$tissue=="thorax"),] # subset data from the dorsal thorax
gon <- q[which(q$tissue=="gonad"),] # subset data from the gonad
ov <- gon[which(gon$sex=="f"),] # subset data from the ovary
te <- gon[which(gon$sex=="m"),] # subset data from the testes
# Group identifiers combining sex and morph or ecotype and morph
dt.grp <- as.factor(paste(dt$sex,dt$morph,sep=""))
gon.grp <- as.factor(paste(gon$sex,gon$morph,sep=""))
ov.grp <- as.factor(paste(gon$hostplant[which(gon$sex=="f")],
                          gon$morph[which(gon$sex=="f")],sep="."))
te.grp <- as.factor(paste(gon$hostplant[which(gon$sex=="m")],
                          gon$morph[which(gon$sex=="m")],sep="."))
host.mrph.grp <-  as.factor(paste(dt$hostplant,dt$morph,sep="."))
```

Before making comparisons within these data, we tested for violations of parametric assumptions, using the Shapiro-Wilk normality test and Levene's test for homogeneity of variance. (These tests are not shown here, but can be viewed in the R markdown code.) When possible, we used ANOVA with individual planned contrasts, although nonparametric methods were used when required by violations of ANOVA assumptions. Gene expression measurements of *InR1*, *InR2* and *FoxO* from the ovaries fit parametric assumptions, but measurements of *FoxO* from the thorax and *vitellogenin* did not.

```
# Dummy variables for planned constrasts
by.host      <- c(1, 1,-1,-1)
by.BV.morph  <- c(1,-1, 0, 0)
by.GRT.morph <- c(0, 0, 1,-1)
contrasts(ov.grp) <- cbind(by.host,by.BV.morph,by.GRT.morph)

# FoxO in ovaries
foxo.ov.aov <- summary.lm(aov(foxo-actb ~ ov.grp, data=ov))
foxo.ov.aov
```

```
##
## Call:
## aov(formula = foxo - actb ~ ov.grp, data = ov)
##
## Residuals:
##      Min       1Q   Median       3Q      Max
## -0.74939 -0.54500  0.08258  0.34066  1.11770
##
## Coefficients:
##                     Estimate Std. Error t value Pr(>|t|)
## (Intercept)          -0.8185     0.1297  -6.309 7.85e-06 ***
## ov.grpby.host        -0.1853     0.1297  -1.428    0.171
## ov.grpby.BV.morph    -0.3831     0.1693  -2.263    0.037 *
## ov.grpby.GRT.morph  -0.0152     0.1967  -0.077    0.939
## ---
## Signif. codes:  0 '***' 0.001 '**' 0.01 '*' 0.05 '.' 0.1 ' ' 1
##
## Residual standard error: 0.5863 on 17 degrees of freedom
## Multiple R-squared:  0.2976, Adjusted R-squared:  0.1736
```

```
## F-statistic: 2.401 on 3 and 17 DF,  p-value: 0.1035
```

```r
foxo.ov.bv.p <- signif(foxo.ov.aov$coefficients["ov.grpby.BV.morph","Pr(>|t|)"],3)

# InR1 in ovaries
inr1.ov.aov <- summary.lm(aov(InR1-actb ~ ov.grp, data=ov))
inr1.ov.aov
```

```
##
## Call:
## aov(formula = InR1 - actb ~ ov.grp, data = ov)
##
## Residuals:
##     Min      1Q  Median      3Q     Max
## -1.0112 -0.6185 -0.1201  0.4300  1.6318
##
## Coefficients:
##                      Estimate Std. Error t value Pr(>|t|)
## (Intercept)           -1.3098     0.1668  -7.854 4.69e-07 ***
## ov.grpby.host         -0.2924     0.1668  -1.753   0.0976 .
## ov.grpby.BV.morph     -0.4439     0.2176  -2.040   0.0572 .
## ov.grpby.GRT.morph     0.1702     0.2528   0.673   0.5097
## ---
## Signif. codes:  0 '***' 0.001 '**' 0.01 '*' 0.05 '.' 0.1 ' ' 1
##
## Residual standard error: 0.7537 on 17 degrees of freedom
## Multiple R-squared:  0.3065, Adjusted R-squared:  0.1842
## F-statistic: 2.505 on 3 and 17 DF,  p-value: 0.09386
```

```r
inr1.ov.eco.p <- signif(inr1.ov.aov$coefficients["ov.grpby.host","Pr(>|t|)"],3)
inr1.ov.bv.p <- signif(inr1.ov.aov$coefficients["ov.grpby.BV.morph","Pr(>|t|)"],3)

# InR2 in ovaries
inr2.ov.aov <- summary.lm(aov(InR2-actb ~ ov.grp, data=ov))
inr2.ov.aov
```

```
##
## Call:
## aov(formula = InR2 - actb ~ ov.grp, data = ov)
##
## Residuals:
##      Min       1Q   Median       3Q      Max
## -1.06682 -0.61071  0.05167  0.54278  1.42840
##
## Coefficients:
##                    Estimate Std. Error t value Pr(>|t|)
## (Intercept)        -1.62724    0.17560  -9.267 4.67e-08 ***
## ov.grpby.host      -0.43232    0.17560  -2.462   0.0248 *
## ov.grpby.BV.morph  -0.38616    0.22909  -1.686   0.1101
## ov.grpby.GRT.morph -0.05161    0.26618  -0.194   0.8486
## ---
## Signif. codes:  0 '***' 0.001 '**' 0.01 '*' 0.05 '.' 0.1 ' ' 1
##
## Residual standard error: 0.7936 on 17 degrees of freedom
## Multiple R-squared:  0.3478, Adjusted R-squared:  0.2327
## F-statistic: 3.022 on 3 and 17 DF,  p-value: 0.05843
```

```r
inr2.ov.eco.p <- signif(inr2.ov.aov$coefficients["ov.grpby.host","Pr(>|t|)"],3)

# FoxO in the dorsal thorax
foxo.dt.morph.wrst <- with(dt, wilcox.test(foxo-actb ~ morph))
foxo.dt.morph.wrst
```

```
##
##  Wilcoxon rank sum test
##
## data:  foxo - actb by morph
## W = 77, p-value = 0.003624
## alternative hypothesis: true location shift is not equal to 0
```

```r
foxo.dt.morph.p <- signif(foxo.dt.morph.wrst$p.value,3)
foxo.dt.bv.morph.p <- signif(with(dt[which(dt$hostplant=="BV"),],
                                  wilcox.test(foxo-actb ~ morph))$p.value,3)
foxo.dt.grt.morph.p <- signif(with(dt[which(dt$hostplant=="GRT"),],
                                   wilcox.test(foxo-actb ~ morph))$p.value,3)
foxo.dt.eco.wrst <- with(dt, wilcox.test(foxo-actb ~ hostplant))
foxo.dt.eco.wrst
```

```
##
##  Wilcoxon rank sum test
##
## data:  foxo - actb by hostplant
## W = 93, p-value = 0.03327
## alternative hypothesis: true location shift is not equal to 0
```

```r
foxo.dt.eco.p <- signif(foxo.dt.eco.wrst$p.value,3)

# Vitellogenin in gonads
vit.sex.wrst <- with(gon, wilcox.test(vit-actb ~ sex))
vit.sex.wrst
```

```
##
##  Wilcoxon rank sum test
##
## data:  vit - actb by sex
## W = 51, p-value = 0.0001708
## alternative hypothesis: true location shift is not equal to 0
```

```r
vit.sex.p <- signif(vit.sex.wrst$p.value,3)
```

Gene expression can be visualized for each gene by morph and sex with individual values overlaid on Tukey's box-whisker plots.

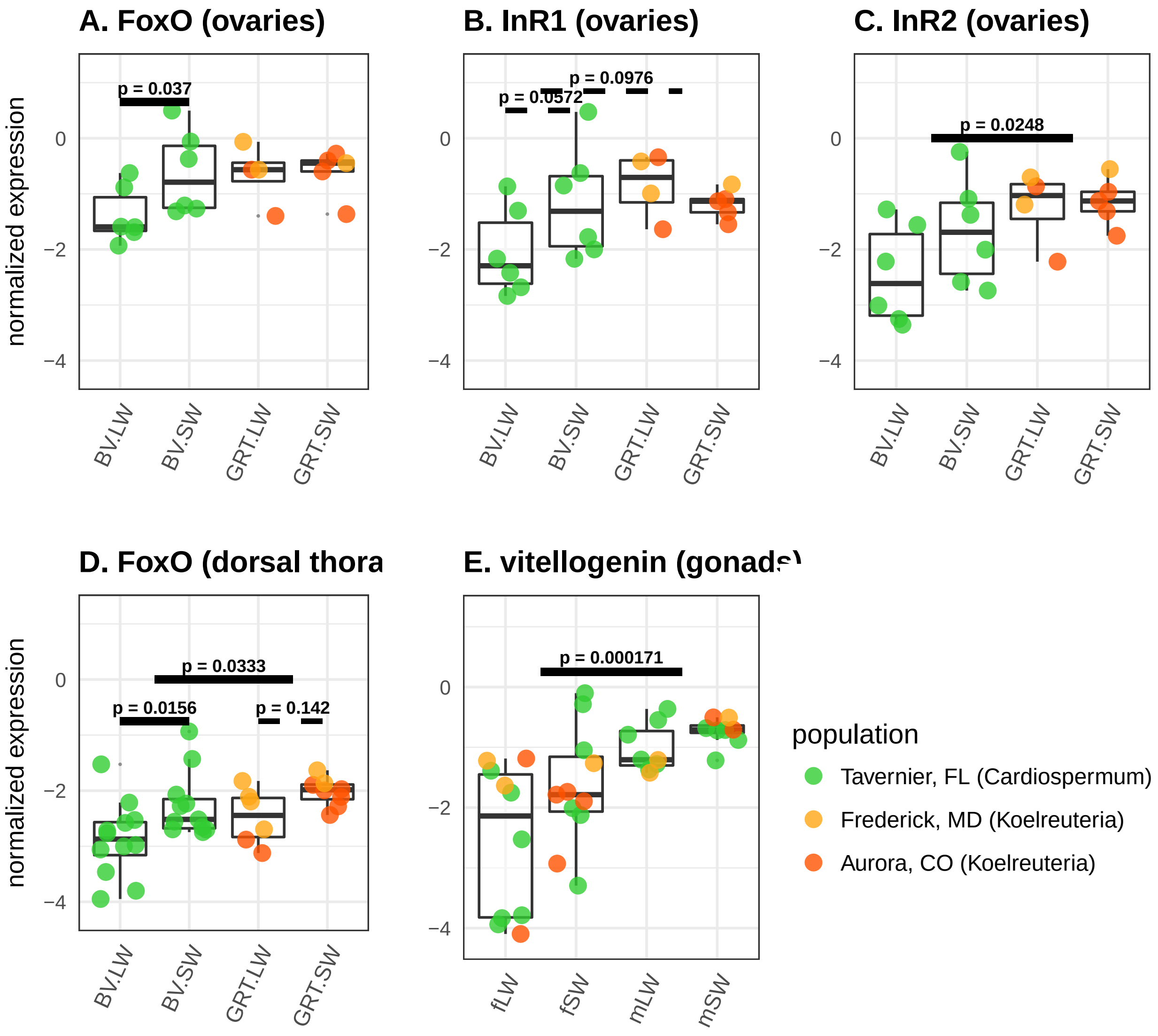

Supplementary Figure 8. Expression of insulin signaling components in *J. haematoloma*. The range of expression is indicated by Tukey's plot: boxes demarcate the upper and lower quartiles, while the heavy bar indicates the median of normalized expression. Whiskers extend to 1.5 times the interquartile range or the most extreme value. Significant differences among groups are denoted by solid bars, while marginal differences are given dashed bars. Comparisons in panels **a-b** are planned ANOVA contrasts. Those in panels **d** and **e** use the Kruskal-Wallis or Wilcoxon tests. Abbreviations: BV, balloon vine / *Cardiospermum*; GRT, goldenrain tree / *Koelreuteria*; LW, long-wing morph; SW, short-wing morph; f, female; m, male.

Expression of candidate genes was examined by qRT-PCR in the dorsal thorax and gonads on the first day of adulthood (Supplementary Figure 8). Expression of the insulin receptor genes did not differ by morph in either tissue. *FoxO* expression was higher on average in samples from short-wing bugs, and this difference was significant in ovaries. Since female fecundity varies among morphs, it is not surprising to find differences in ovary gene expression. Expression of the yolk protein gene *vitellogenin* was higher in short-wing ovaries, but not significantly so. Unexpectedly, *vitellogenin* was also up-regulated in male gonads. Similar expression has also been found in the seminal fluid of the African cotton leaf worm where it is required for sperm function[23].

Gene interactions

We also used qRT-PCR to validate RNAi knockdown and to test for potential gene interactions.

```
qr <- read.csv("qPCR.RNAi.data.csv",header=T)
```

The data were tested for normality and homogeneity of variance and meet assumptions for the use of ANOVA. (These tests are not shown here, but can be viewed in the R markdown code.)

```
# InR1
summary(aov(qr$InR1-qr$actb ~ qr$dsRNA))
```

```
##             Df Sum Sq Mean Sq F value  Pr(>F)
## qr$dsRNA     2  23.33  11.665   61.67 4.4e-15 ***
## Residuals   58  10.97   0.189
## ---
## Signif. codes:  0 '***' 0.001 '**' 0.01 '*' 0.05 '.' 0.1 ' ' 1
## 23 observations deleted due to missingness
```

```
inr1.tukeys <- TukeyHSD(aov(qr$InR1-qr$actb ~ qr$dsRNA))
inr1.tukeys
```

```
##   Tukey multiple comparisons of means
##     95% family-wise confidence level
##
## Fit: aov(formula = qr$InR1 - qr$actb ~ qr$dsRNA)
##
## $`qr$dsRNA`
##                  diff        lwr        upr   p adj
## GFP-FoxO    0.8598624  0.5546048  1.1651200 0.0e+00
## InR12-FoxO -0.7444826 -1.0962935 -0.3926717 1.2e-05
## InR12-GFP  -1.6043451 -1.9589625 -1.2497276 0.0e+00
```

```
inr1.p.gfp.v.foxo <- signif(inr1.tukeys[[1]]['GFP-FoxO','p adj'],3)
inr1.p.gfp.v.inr12 <- signif(inr1.tukeys[[1]]['InR12-GFP','p adj'],3)

# InR2
summary(aov(qr$InR2-qr$actb ~ qr$dsRNA))
```

```
##             Df Sum Sq Mean Sq F value  Pr(>F)
## qr$dsRNA     2  6.933   3.467   19.42 3.5e-07 ***
## Residuals   58 10.354   0.179
## ---
## Signif. codes:  0 '***' 0.001 '**' 0.01 '*' 0.05 '.' 0.1 ' ' 1
## 23 observations deleted due to missingness
```

```
inr2.tukeys <- TukeyHSD(aov(qr$InR2-qr$actb ~ qr$dsRNA))
inr2.p.gfp.v.inr12 <- signif(inr2.tukeys[[1]]['InR12-GFP','p adj'],3)

# FoxO
summary(aov(qr$foxo-qr$actb ~ qr$dsRNA))
```

```
##             Df Sum Sq Mean Sq F value   Pr(>F)
## qr$dsRNA     2  2.204   1.102    9.11 0.000363 ***
## Residuals   58  7.018   0.121
## ---
## Signif. codes:  0 '***' 0.001 '**' 0.01 '*' 0.05 '.' 0.1 ' ' 1
## 23 observations deleted due to missingness
```

```r
foxo.tukeys <- TukeyHSD(aov(qr$foxo-qr$actb ~ qr$dsRNA))
foxo.tukeys
```

```
##   Tukey multiple comparisons of means
##     95% family-wise confidence level
##
## Fit: aov(formula = qr$foxo - qr$actb ~ qr$dsRNA)
##
## $`qr$dsRNA`
##                   diff         lwr        upr     p adj
## GFP-FoxO     0.3003173  0.05618323  0.5444513 0.0122326
## InR12-FoxO -0.1788512 -0.46021687  0.1025145 0.2850322
## InR12-GFP  -0.4791685 -0.76277870 -0.1955582 0.0004267
```

```r
foxo.p.gfp.v.foxo <- signif(foxo.tukeys[[1]]['GFP-FoxO','p adj'],3)
foxo.p.gfp.v.inr12 <- signif(foxo.tukeys[[1]]['InR12-GFP','p adj'],3)

# vitellogenin
summary(aov(qr$vit-qr$actb ~ qr$dsRNA))
```

```
##             Df Sum Sq Mean Sq F value  Pr(>F)
## qr$dsRNA     2  3.735   1.867   6.483 0.00291 **
## Residuals   57 16.418   0.288
## ---
## Signif. codes:  0 '***' 0.001 '**' 0.01 '*' 0.05 '.' 0.1 ' ' 1
## 24 observations deleted due to missingness
```

```r
vit.tukeys <- TukeyHSD(aov(qr$vit-qr$actb ~ qr$dsRNA))
vit.tukeys
```

```
##   Tukey multiple comparisons of means
##     95% family-wise confidence level
##
## Fit: aov(formula = qr$vit - qr$actb ~ qr$dsRNA)
##
## $`qr$dsRNA`
##                   diff        lwr        upr     p adj
## GFP-FoxO   -0.0278004 -0.4090034  0.3534027 0.9831693
## InR12-FoxO -0.6024619 -1.0367886 -0.1681351 0.0041946
## InR12-GFP  -0.5746615 -1.0162017 -0.1331212 0.0076061
```

```r
vit.p.gfp.v.inr12 <- signif(vit.tukeys[[1]]['InR12-GFP','p adj'],3)
```

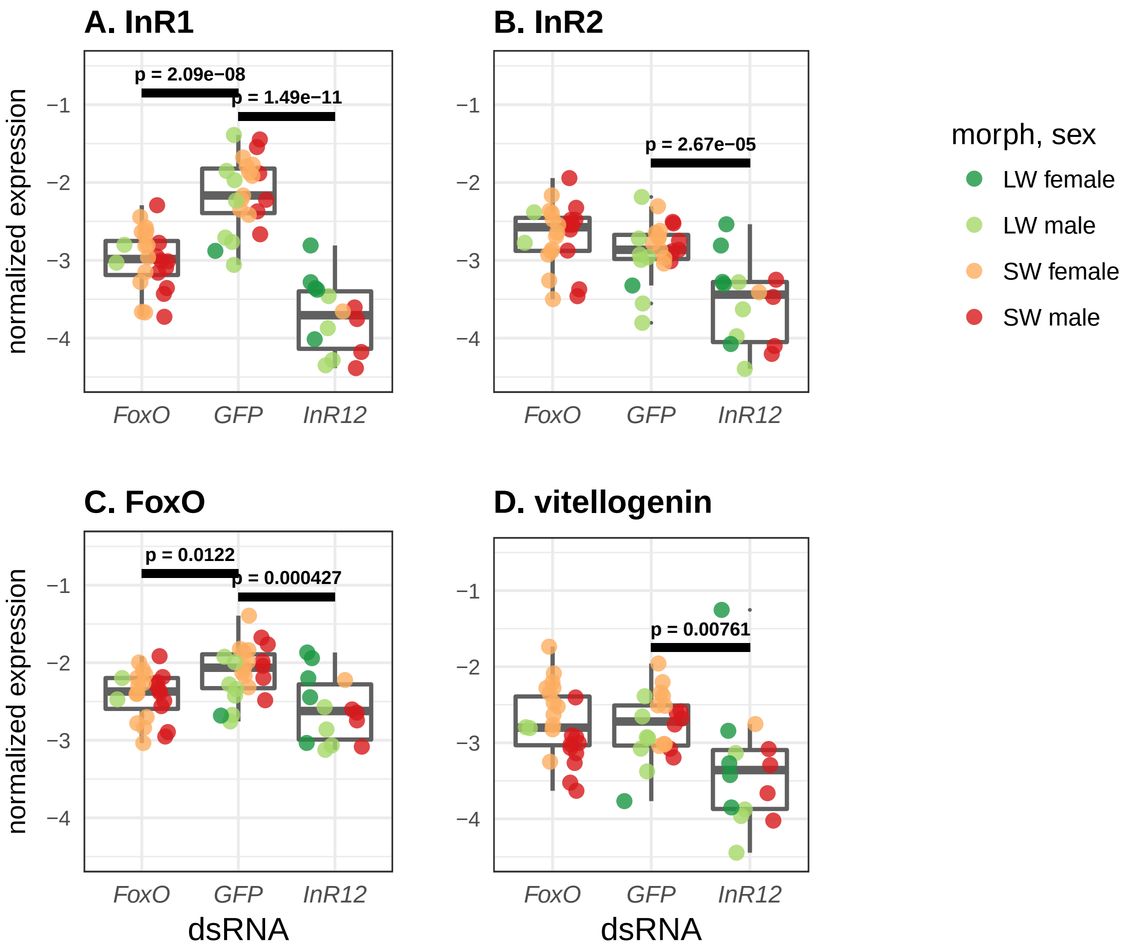

Supplementary Figure 9. Gene interactions as revealed by expression of insulin signaling components in an RNAi background. Normalized gene expression from the whole body of nascent adult RNAi specimens is shown by Tukey's plots. Significant differences identified by Tukey's post hoc test are denoted by bars. Each gene targeted by RNAi showed a significant reduction in expression. In addition, several gene interactions were identified. One or both insulin receptors promote expression of *FoxO* (**c**), and FoxO promotes expression of *InR1* (**a**), but not *InR2* (**b**). Expression of *vitellogenin* is activated by one or both insulin receptors (**d**).

Manipulation of insulin signaling

Since insulin signaling varies in different *J. haematoloma* morphs and ecotypes, we examined how manipulation of insulin signaling affected wing morph frequencies, appendage allometry, and wing shapes. Gene function was tested during juvenile-to-adult development in *J. haematoloma* using RNA interference[24,25]. Knockdown of gene activity was confirmed using quantitative RT-PCR (Supplementary Figure 9).

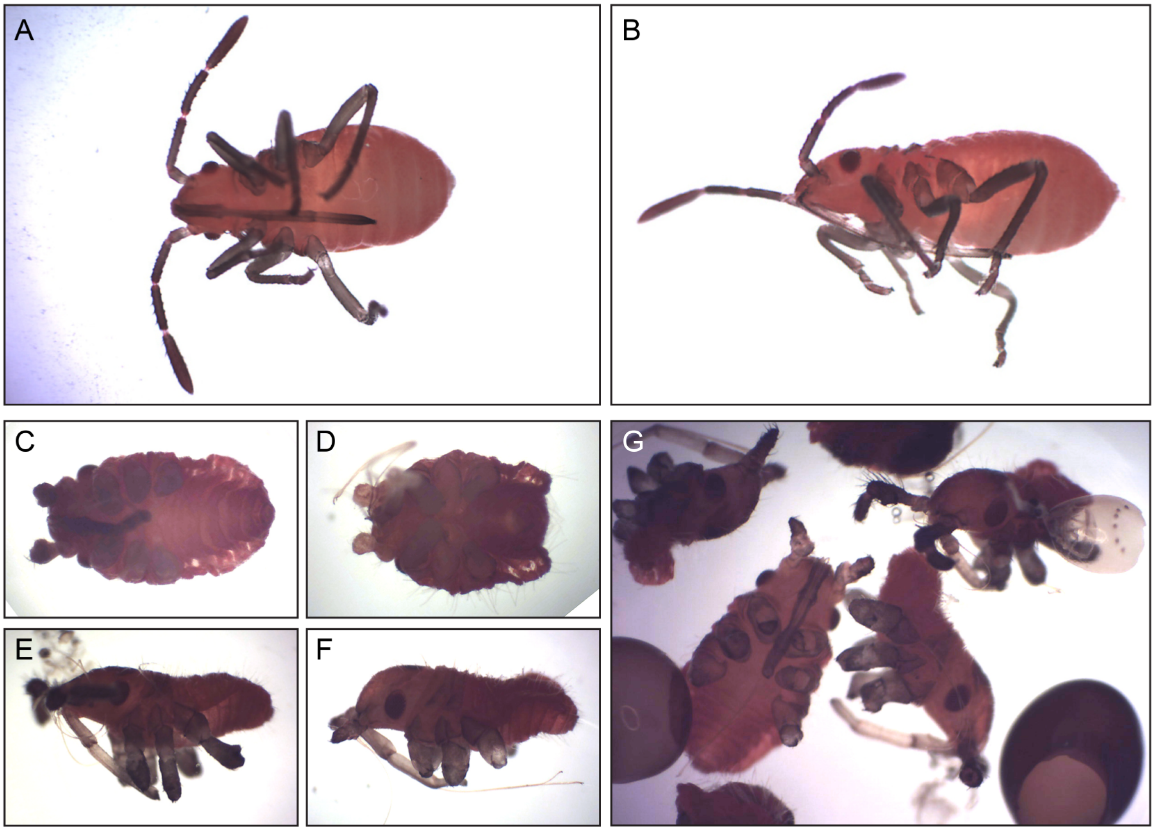

Supplementary Figure 10. Demonstration of RNAi effectiveness during *J. haematoloma* embryonic development. (**a**) Ventral image of an unmanipulated first instar hatchling. (**b**) Lateral image of an unmanipulated hatchling. (**c-g**) Virgin females were injected with dsRNA targeting the appendage-development gene *Distal-less* (*Dll*). (**c-d**) Ventral views of resulting first instars from *Dll* maternal RNAi. These individuals lack development of distal regions of all appendages, except the stylets. (**e-f**) Lateral images of *Dll* RNAi hatchlings. (**g**) Additional *Dll* RNAi hatchlings.

RNA interference is effective in this species during embryonic development as well. Targeting the appendage-patterning gene *Distal-less* in *J. haematoloma* (Supplementary Figure 10), we were able to phenocopy the distal appendage deletion produced by *Distal-less* RNAi in the milkweed bug *Oncopeltus fasciatus*[26].

We also treated juveniles with exogenous insulin[27,28]. Bovine pancreatic insulin (Sigma-Aldrich, St. Louis, Missouri, USA) was prepared in phosphate-buffered saline and injected into the abdomen of fourth instars to deliver roughly 0.5 ng insulin per mg body weight.

```
rxn.all.data <- read.csv("reaction.norms.csv", header=TRUE)
# Subset data for RNAi and control treatments (excludes cross-rearing and hybrids)
rxn.i <- dplyr::filter(rxn.all.data, as.character(ecotype)==as.character(seedspecies) & !is.na(pSW))
# Clean-up and re-order category names
rxn.i$ecotype <- plyr::revalue(rxn.i$ecotype, c("BV"="Cardiospermum","GRT"="Koelreuteria"))
rxn.i$treatment <- factor(rxn.i$treatment, levels = c("unmanip", "GFP",
                                                      "InR01", "InR02", "InR12",
                                                      "FoxO", "insulin"))
rxn.i$treatment <- plyr::revalue(rxn.i$treatment, c("unmanip"="unmanipulated",
                                             "GFP"= "ds GFP",
                                             "InR01"="ds InR1",
                                             "InR02"="ds InR2",
                                             "InR12"="ds InR1&2",
```

```
                                                  "FoxO"="ds FoxO"))
rxn.i$pop <- droplevels(rxn.i$pop)
rxn.i$pop <- factor(rxn.i$pop, levels = c("PlantationKey", "KeyLargo",
                                                "Greenbrier", "Frederick", "Aurora"))
```

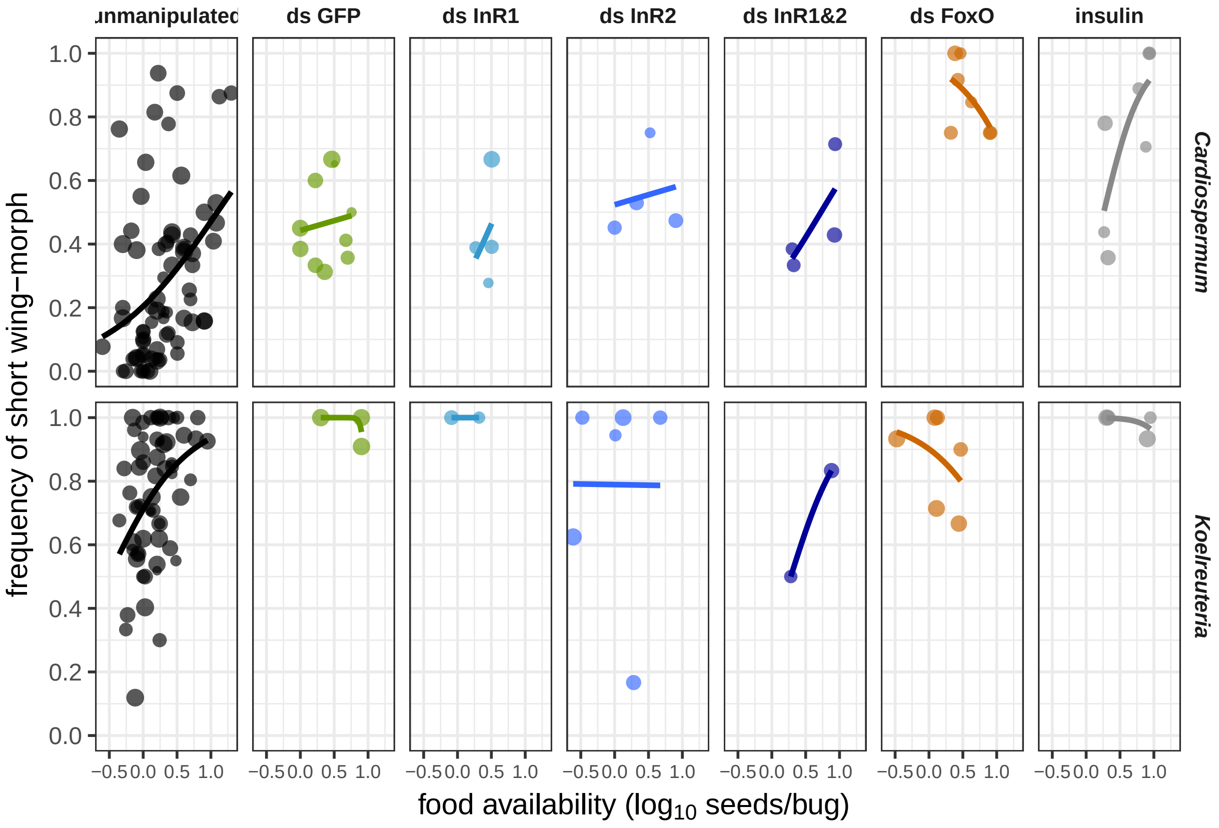

This plot was modified in Adobe *Illustrator* to produce Figure 5.

Modeling experimental effects on morph frequencies

Since we have already established that the wing morph of individual bugs can be modeled by seed number, cohort size and ecotype, we will examine the effects of RNAi and insulin treatments in the context of these factors. In order to make unmanipulated treatments more comparable, we restricted these data to food availability metrics no more than 20% of the maximum used in experimental treatments or less than 80% of the minimum.

```
rxn.i$foodavailability <- rxn.i$seeds / rxn.i$cohort
max.rnai.food <- max(rxn.i$foodavailability[which(rxn.i$treatment!="unmanipulated")])
min.rnai.food <- min(rxn.i$foodavailability[which(rxn.i$treatment!="unmanipulated")])
rxn.i <- dplyr::filter(rxn.i, (foodavailability < 1.2*max.rnai.food) &
                                (foodavailability > 0.8*min.rnai.food))
models.for.rnai <- c("1","0","seeds","cohort","ecotype","seeds + cohort","seeds * cohort",
                     "seeds + ecotype","seeds * ecotype","cohort + ecotype","cohort * ecotype",
                     "seeds + cohort + ecotype","seeds * cohort + ecotype",
                     "seeds + cohort * ecotype","seeds * cohort * ecotype",
                     "seeds + treatment","cohort + treatment","ecotype + treatment",
                     "seeds + cohort + treatment","seeds * cohort + treatment",
                     "seeds + ecotype + treatment","seeds * ecotype + treatment",
```

```
                        "cohort + ecotype + treatment","cohort * ecotype + treatment",
                        "seeds + cohort + ecotype + treatment","seeds * cohort + ecotype + treatment",
                        "seeds + cohort * ecotype + treatment",
                        "seeds * cohort * ecotype + treatment",
                        "seeds * treatment","cohort * treatment","ecotype * treatment",
                        "seeds + cohort * treatment","seeds * cohort * treatment",
                        "seeds + ecotype * treatment","seeds * ecotype * treatment",
                        "cohort + ecotype * treatment","cohort * ecotype * treatment",
                        "seeds + cohort + ecotype * treatment",
                        "seeds * cohort + ecotype * treatment","seeds + cohort * ecotype * treatment",
                        "seeds * cohort * ecotype * treatment")
lra.rnai <- logistic.reg.analysis(rxn.i, models.for.rnai)
knitr::kable(lra.rnai$results.by.aic[1:10,c(1:3,5)]) # Top 10 models
```

	AIC	x^2	df	$R^2{}_{\text{H\&L}}$
seeds * cohort + ecotype * treatment	1361.868	1212.73	16	0.5823
seeds + cohort + ecotype * treatment	1364.297	1208.30	15	0.5802
seeds * cohort * ecotype * treatment	1366.668	1269.93	47	0.6098
seeds * cohort + ecotype + treatment	1370.025	1192.57	10	0.5726
seeds * cohort * ecotype + treatment	1372.290	1196.31	13	0.5744
seeds + cohort + ecotype + treatment	1373.390	1187.21	9	0.5701
seeds + cohort * ecotype + treatment	1374.726	1187.87	10	0.5704
seeds + cohort * ecotype * treatment	1374.846	1219.75	26	0.5857
cohort + ecotype * treatment	1398.710	1171.89	14	0.5627
cohort * ecotype * treatment	1404.822	1187.77	25	0.5703

Supplementary Table 11. Ten best models, based on AIC, evaluated in logistic regression for specification of wing morphs in the context of RNA interference and insulin treatments. Abbreviations as in Supplementary Table 3.

Of the 41 models in this analysis, AIC consistently favored those including experimental treatment as a factor.

```
# Calculate odds ratios and confidence intervals for each factor
lra.rnai.or <- odds.confidence(lra.rnai)
# Compare the influence of each treatment to GFP dsRNA, using the Wald test
lra.rnai.or.wald <- wald.or.comparison(lra.rnai.or, ref=5, comps=c(6:10))
knitr::kable(lra.rnai.or.wald[1:11,1:7])
```

	B	std. err.	p		2.5% CI	odds ratio	97.5% CI
(Intercept)	-0.9349000	0.1734000	0.0000001	***	0.2791	0.3926	0.5509
seeds	0.0062150	0.0015920	0.0000949	***	1.0030	1.0060	1.0090
cohort	-0.0107200	0.0027540	0.0000992	***	0.9840	0.9893	0.9947
ecotypeKoelreuteria	2.4510000	0.0893300	0.0000000	***	9.7500	11.6000	13.8400
treatmentds GFP	0.6875000	0.1786000	0.0001189	***	1.4000	1.9890	2.8220
treatmentds InR1	0.6127000	0.2354000	0.0092410	**	1.1570	1.8450	2.9210
treatmentds InR2	1.0270000	0.2248000	0.0000049	***	1.8010	2.7940	4.3570
treatmentds InR1&2	0.4744000	0.2892000	0.1010000		0.9065	1.6070	2.8330
treatmentds FoxO	2.8920000	0.3442000	0.0000000	***	9.6200	18.0200	37.6000
treatmentinsulin	1.7170000	0.2326000	0.0000000	***	3.5710	5.5670	8.9160
seeds:cohort	-0.0000456	0.0000217	0.0359800	*	0.9999	1.0000	1.0000

Supplementary Table 12. Coefficients (log odds) and odds ratios for factors predicting wing

morph outcomes, including the influence of dsRNA and insulin treatments. Confidence intervals (95%) are given for the odds ratios.

To determine the influence of individual treatments, in the context of effects of food availability, we examined odds ratios for each factor in the favored model, seeds * cohort + ecotype * treatment (Supplementary Table 12). Since comparisons of treatments to unmanipulated controls, as well as to non-specific dsRNA controls are desirable, we define a function to compare log odds directly using the Wald test (Supplementary Table 13).

	z	p	
ds *InR1*	-0.2531	0.8002	
ds *InR2*	1.1820	0.2372	
ds *InR1&2*	-0.6269	0.5307	
ds *FoxO*	5.6850	0.0000	***
insulin	3.5110	0.0004	***

Supplementary Table 13. The influence of RNAi and insulin treatments were compared to non-specific *GFP* dsRNA control treatments using the Wald test. The frequency of short-wing morphs is significantly increased by *FoxO* RNAi and insulin injection. Significance is indicated by asterisks.

As in the unmanipulated dataset, the number of seeds, cohort size and ecotype were significant predictors of wing morph development. The control *GFP* dsRNA appears to be a significant predictor in this analysis, relative to unmanipulated *Cardiospermum* bugs. This may be due to a developmental effect of wounding after injection.

Compared to *GFP* dsRNA treatment, RNAi targeting the insulin receptor genes, alone and in combination, did not significantly influence morph outcomes (Wald test, $p > 0.05$). However knockdown of *FoxO* by RNAi significantly increased the odds of bugs developing to the short-wing morph (odds ratio = 18.0; Wald test vs. *GFP* RNAi $z = 5.69$, $p = 1.31\text{x}10^{-8}$). Similarly, injection of bovine insulin significantly increased the odds of short-wing development (odds ratio = 5.57; Wald test $z = 3.51$, $p = 4.46\text{x}10^{-4}$).

Treatment effects within each ecotype

Ecotypes already differ in their response to food availability, and manipulations of insulin signaling are constrained by the fact that, for example, *Koelreuteria* bugs are already highly prone to short-wing fates. Therefore, we also examined the effects of RNAi and insulin treatments on each ecotype separately. To do so, we applied a model include the factors `seeds * cohort + treatment` after subsetting *Cardiospermum* and *Koelreuteria* samples. The Wald test was then used to compare each treatment to *GFP* dsRNA controls.

```
rxn.bv.i <- dplyr::filter(rxn.i, ecotype=='Cardiospermum')
lra.rnai.or.bv <- odds.confidence(logistic.reg.analysis(rxn.bv.i, "seeds * cohort + treatment"))
lra.rnai.or.bv.wald <- wald.or.comparison(lra.rnai.or.bv, ref=4, comps=c(5:9))

rxn.grt.i <- dplyr::filter(rxn.i, ecotype=='Koelreuteria')
lra.rnai.or.grt <- odds.confidence(logistic.reg.analysis(rxn.grt.i, "seeds * cohort + treatment"))
lra.rnai.or.grt.wald <- wald.or.comparison(lra.rnai.or.grt, ref=4, comps=c(5:9))
```

	Cardiospermum z	p		*Koelreuteria* z	p	
ds *InR1*	-0.2455	0.8061		0.0212	0.9831	
ds *InR2*	1.1670	0.2432		-0.8291	0.4070	
ds *InR1&2*	-0.4392	0.6605		-1.8180	0.0691	.
ds *FoxO*	5.5840	0.0000	***	-0.3820	0.7025	
insulin	3.5250	0.0004	***	0.2759	0.7826	

Supplementary Table 14. Results of Wald's test comparing the effects of RNAi and insulin treatments to controls in each ecotype. *FoxO* RNAi and insulin treatment produce significant increases in the rate of short-wing adults in *Cardiospermum* ecotype bugs, but not in *Koelreuteria* ecotypes, which are already commonly short-winged. In contrast, among *Koelreuteria* ecotypes, RNAi targetting both insulin receptor genes produces a marginal decreases in the frequency of short-wing morphs, compared to *GFP* dsRNA treatments.

FoxO RNAi (z = 5.584, p = 2.35x10^{-8}) and insulin injection (z = 3.525, p = 4.235x10^{-4}) where significant factors in short-wing development within the *Cardiospermum* ecotype, as they were in the overall dataset. In the *Koelreuteria* ecotype, RNAi targeting *InR1* and *InR2* simultaneously produced fewer short-wing bugs compared to controls, although this effect was marginal (z = -1.818, p = 0.0691).

RNAi targeting *chico* and *Akt*

The knockdown of two additional insulin pathway components, encoded by *chico* and *Akt*, extended the fifth instar and prevented the adult molt, precluding a determination of the influence these genes have on morph specification. While the fifth instar normally lasts for 5-10 days, *chico* RNAi individuals remained as fifth instars for up to 87 days. During this time, growth continued slowly without molting. The abdomens of bugs became swollen and dissection revealed large amounts of stored lipids. *Akt* RNAi similarly prevented molting and extended the duration of the fifth instar for up to 54 days, but excessive lipid storage was not observed. Extended lifespan and developmental delay have also been found for *chico* and *Akt* mutants in *Drosophila*[29–31].

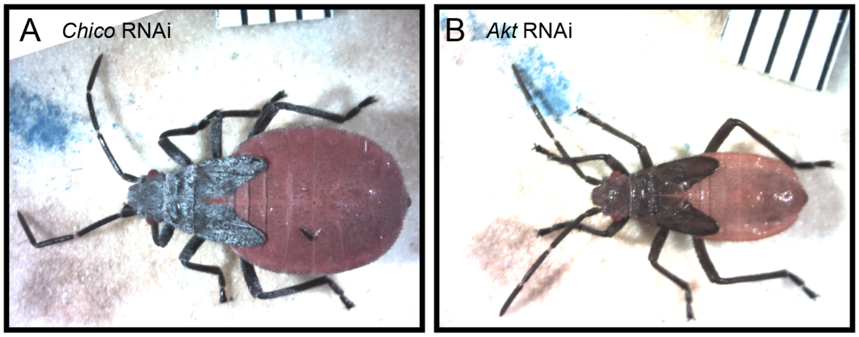

Supplementary Figure 11. RNAi targeting (**a**) *Chico* and (**b**) *Akt* blocked the molt to adulthood (19 of 20 individuals). *Chico* RNAi individuals also became swollen over time (12 of 16 individuals). Both treatments experienced extended lifespans. Ruler divisions are 1 mm.

Effects of RNAi on allometric growth

Since insulin signaling is thought to mediate allometric growth of organs throughout the insect body, we examined RNAi specimens for differences in the growth of appendages and other structures.

```
rnai.linear <- read.csv("RNAi.linear.csv", header = T)
```

This dataset includes measurements of head width (dorsal anterior interocular distance, `daid`), body length (`bl`), labrum length (`lr`), beak length (`lb`), mean antenna length (`an`), mean femora lengths (`f1`, `f2`, `f3`), pronotum width (`pw`), and forewing length (`fwl`) from 0 *InR1* RNAi specimens, 0 *FoxO* RNAi specimens, and 0 *GFP* dsRNA controls. The wing length is already known to vary by morph, long- and short-winged specimens were considered separately.

```
lw <- rnai.linear[which(rnai.linear$morph=="LW"),]
sw <- rnai.linear[which(rnai.linear$morph=="SW"),]
```

Parametric assumptions were tested as described above for gene expression data. See the associated `Rmd` file for details of the code. Results are summarized in Supplementary Tables 15 and 16.

	trait	Homogenious var.	Violates normality	Meets ANOVA assumptions
pronotum width	pw	TRUE	0	TRUE
body length	bl	TRUE	0	TRUE
beak length	lb	TRUE	0	TRUE
labrum length	lr	TRUE	0	TRUE
antenna length	an	TRUE	1	FALSE
femor length (T1)	f1	FALSE	0	FALSE
femor length (T2)	f2	TRUE	1	FALSE
femor length (T3)	f3	TRUE	2	FALSE
forewing length	fwl	TRUE	0	TRUE

Supplementary Table 15. Summary of ANOVA assumptions met by linear morphometrics of long-wing RNAi and control specimens.

	trait	Homogenious var.	Violates normality	Meets ANOVA assumptions
pronotum width	pw	FALSE	0	FALSE
body length	bl	FALSE	1	FALSE
beak length	lb	FALSE	0	FALSE
labrum length	lr	TRUE	0	TRUE
antenna length	an	TRUE	0	TRUE
femor length (T1)	f1	FALSE	0	FALSE
femor length (T2)	f2	TRUE	1	FALSE
femor length (T3)	f3	TRUE	0	TRUE
forewing length	fwl	TRUE	1	FALSE

Supplementary Table 16. Summary of ANOVA assumptions met by linear morphometrics of short-wing RNAi and control specimens.

For each measurement, ANOVA was applied where parametric assumptions were met, otherwise the Kruskal-Wallis rank sum test was used. Where significant effects were found, post hoc tests were made between the *GFP* dsRNA controls and each of the other treatments using either Welch's t-test or the Wilcoxon rank sum test. Significant post-hoc results have been used to annotate Supplementary Figure 12.

```
# pronotum width (pw)
with(lw, summary(aov(pw/daid ~ treatment)))
```

```
##             Df Sum Sq Mean Sq F value  Pr(>F)
## treatment    2 0.2579 0.12896   7.838 0.00207 **
## Residuals   27 0.4442 0.01645
## ---
## Signif. codes:  0 '***' 0.001 '**' 0.01 '*' 0.05 '.' 0.1 ' ' 1
```

```
with(lw[which(lw$treatment=="dsGFP" | lw$treatment=="dsInR01"),],
     t.test(pw/daid ~ treatment))$p.value %>%
  p.adjust(method="bonferroni",n=2) %>% signif(4) %>% print() -> pw.lw.gfp.inr1
```

```
## [1] 0.01264
```

```
with(lw[which(lw$treatment=="dsGFP" | lw$treatment=="dsFoxO"),],
     t.test(pw/daid ~ treatment))$p.value %>%
  p.adjust(method="bonferroni",n=2) %>% signif(4) %>% print() -> pw.lw.gfp.foxo
```

```
## [1] 0.5069
```

```r
with(sw, kruskal.test(pw/daid ~ treatment))
```

```
##
##  Kruskal-Wallis rank sum test
##
## data:  pw/daid by treatment
## Kruskal-Wallis chi-squared = 11.267, df = 2, p-value = 0.003576
```

```r
with(sw[which(sw$treatment=="dsGFP" | sw$treatment=="dsInR01"),],
     wilcox.test(pw/daid ~ treatment))$p.value %>%
  p.adjust(method="bonferroni",n=2) %>% signif(4) %>% print() -> pw.sw.gfp.inr1
```

```
## [1] 0.00934
```

```r
with(sw[which(sw$treatment=="dsGFP" | sw$treatment=="dsFoxO"),],
     wilcox.test(pw/daid ~ treatment))$p.value %>%
  p.adjust(method="bonferroni",n=2) %>% signif(4) %>% print() -> pw.sw.gfp.foxo
```

```
## [1] 0.7345
```

```r
# body length (bl)
with(lw, summary(aov(bl/daid ~ treatment)))
```

```
##             Df Sum Sq Mean Sq F value  Pr(>F)
## treatment    2  5.174  2.5869   12.17 0.00017 ***
## Residuals   27  5.738  0.2125
## ---
## Signif. codes:  0 '***' 0.001 '**' 0.01 '*' 0.05 '.' 0.1 ' ' 1
```

```r
with(lw[which(lw$treatment=="dsGFP" | lw$treatment=="dsInR01"),],
     t.test(bl/daid ~ treatment))$p.value %>%
  p.adjust(method="bonferroni",n=2) %>% signif(4) %>% print() -> bl.lw.gfp.inr1
```

```
## [1] 0.9531
```

```r
with(lw[which(lw$treatment=="dsGFP" | lw$treatment=="dsFoxO"),],
     t.test(bl/daid ~ treatment))$p.value %>%
  p.adjust(method="bonferroni",n=2) %>% signif(4) %>% print() -> bl.lw.gfp.foxo
```

```
## [1] 0.01336
```

```r
with(sw, kruskal.test(bl/daid ~ treatment))
```

```
##
##  Kruskal-Wallis rank sum test
##
## data:  bl/daid by treatment
## Kruskal-Wallis chi-squared = 7.693, df = 2, p-value = 0.02135
```

```r
with(sw[which(sw$treatment=="dsGFP" | sw$treatment=="dsInR01"),],
     wilcox.test(bl/daid ~ treatment))$p.value %>%
  p.adjust(method="bonferroni",n=2) %>% signif(4) %>% print() -> bl.sw.gfp.inr1
```

```
## [1] 0.6228
```

```r
with(sw[which(sw$treatment=="dsGFP" | sw$treatment=="dsFoxO"),],
     wilcox.test(bl/daid ~ treatment))$p.value %>%
  p.adjust(method="bonferroni",n=2) %>% signif(4) %>% print() -> bl.sw.gfp.foxo
```

```
## [1] 0.07131
```

```r
# beak / labium length (lb)
with(lw, summary(aov(lb/daid ~ treatment)))
```

```
##             Df Sum Sq Mean Sq F value Pr(>F)
## treatment    2  0.241  0.1205   0.997  0.382
## Residuals   27  3.263  0.1209
```

```r
with(sw, kruskal.test(lb/daid ~ treatment))
```

```
##
##  Kruskal-Wallis rank sum test
##
## data:  lb/daid by treatment
## Kruskal-Wallis chi-squared = 0.28068, df = 2, p-value = 0.8691
```

```r
# labrum length (lr)
with(lw, summary(aov(lr/daid ~ treatment)))
```

```
##             Df  Sum Sq  Mean Sq F value Pr(>F)
## treatment    2 0.02863 0.014316   1.552   0.23
## Residuals   27 0.24908 0.009225
```

```r
with(sw, summary(aov(lr/daid ~ treatment)))
```

```
##             Df  Sum Sq  Mean Sq F value Pr(>F)
## treatment    2 0.02639 0.013195   2.072  0.138
## Residuals   45 0.28652 0.006367
```

```r
# antenna length (an)
with(lw, kruskal.test(an/daid ~ treatment))
```

```
##
##  Kruskal-Wallis rank sum test
##
## data:  an/daid by treatment
## Kruskal-Wallis chi-squared = 3.3525, df = 2, p-value = 0.1871
```

```r
with(sw, summary(aov(an/daid ~ treatment)))
```

```
##             Df Sum Sq Mean Sq F value Pr(>F)
## treatment    2  0.412  0.2058   1.661  0.203
## Residuals   39  4.831  0.1239
## 6 observations deleted due to missingness
```

```r
# femor length (f1, f2, f3)
with(lw, kruskal.test(f1/daid ~ treatment))
```

```
##
##  Kruskal-Wallis rank sum test
##
## data:  f1/daid by treatment
## Kruskal-Wallis chi-squared = 1.0346, df = 2, p-value = 0.5961
```

```r
with(sw, kruskal.test(f1/daid ~ treatment))
```

```
##
##  Kruskal-Wallis rank sum test
##
```

```
## data:  f1/daid by treatment
## Kruskal-Wallis chi-squared = 1.6964, df = 2, p-value = 0.4282
```

```r
with(lw, kruskal.test(f2/daid ~ treatment))
```

```
##
##  Kruskal-Wallis rank sum test
##
## data:  f2/daid by treatment
## Kruskal-Wallis chi-squared = 1.7524, df = 2, p-value = 0.4164
```

```r
with(sw, kruskal.test(f2/daid ~ treatment))
```

```
##
##  Kruskal-Wallis rank sum test
##
## data:  f2/daid by treatment
## Kruskal-Wallis chi-squared = 0.67711, df = 2, p-value = 0.7128
```

```r
with(lw, kruskal.test(f3/daid ~ treatment))
```

```
##
##  Kruskal-Wallis rank sum test
##
## data:  f3/daid by treatment
## Kruskal-Wallis chi-squared = 1.3858, df = 2, p-value = 0.5001
```

```r
with(sw, summary(aov(f3/daid ~ treatment)))
```

```
##             Df Sum Sq Mean Sq F value Pr(>F)
## treatment    2 0.0342 0.01709   0.479  0.622
## Residuals   44 1.5691 0.03566
## 1 observation deleted due to missingness
```

```r
# forewing length (fwl)
with(lw, summary(aov(fwl/daid ~ treatment)))
```

```
##             Df Sum Sq Mean Sq F value Pr(>F)
## treatment    2  1.146  0.5731   3.925 0.0319 *
## Residuals   27  3.943  0.1460
## ---
## Signif. codes:  0 '***' 0.001 '**' 0.01 '*' 0.05 '.' 0.1 ' ' 1
```

```r
with(lw[which(lw$treatment=="dsGFP" | lw$treatment=="dsInR01"),],
     t.test(fwl/daid ~ treatment))$p.value %>%
  p.adjust(method="bonferroni",n=2) %>% signif(4) %>% print()
```

```
## [1] 0.1773
```

```r
with(lw[which(lw$treatment=="dsGFP" | lw$treatment=="dsFoxO"),],
     t.test(fwl/daid ~ treatment))$p.value %>%
  p.adjust(method="bonferroni",n=2) %>% signif(4) %>% print()
```

```
## [1] 0.5056
```

```r
with(sw, kruskal.test(fwl/daid ~ treatment))
```

```
##
##  Kruskal-Wallis rank sum test
##
```

```
## data:  fwl/daid by treatment
## Kruskal-Wallis chi-squared = 1.3241, df = 2, p-value = 0.5158
```

```
## pdf
##   2
```

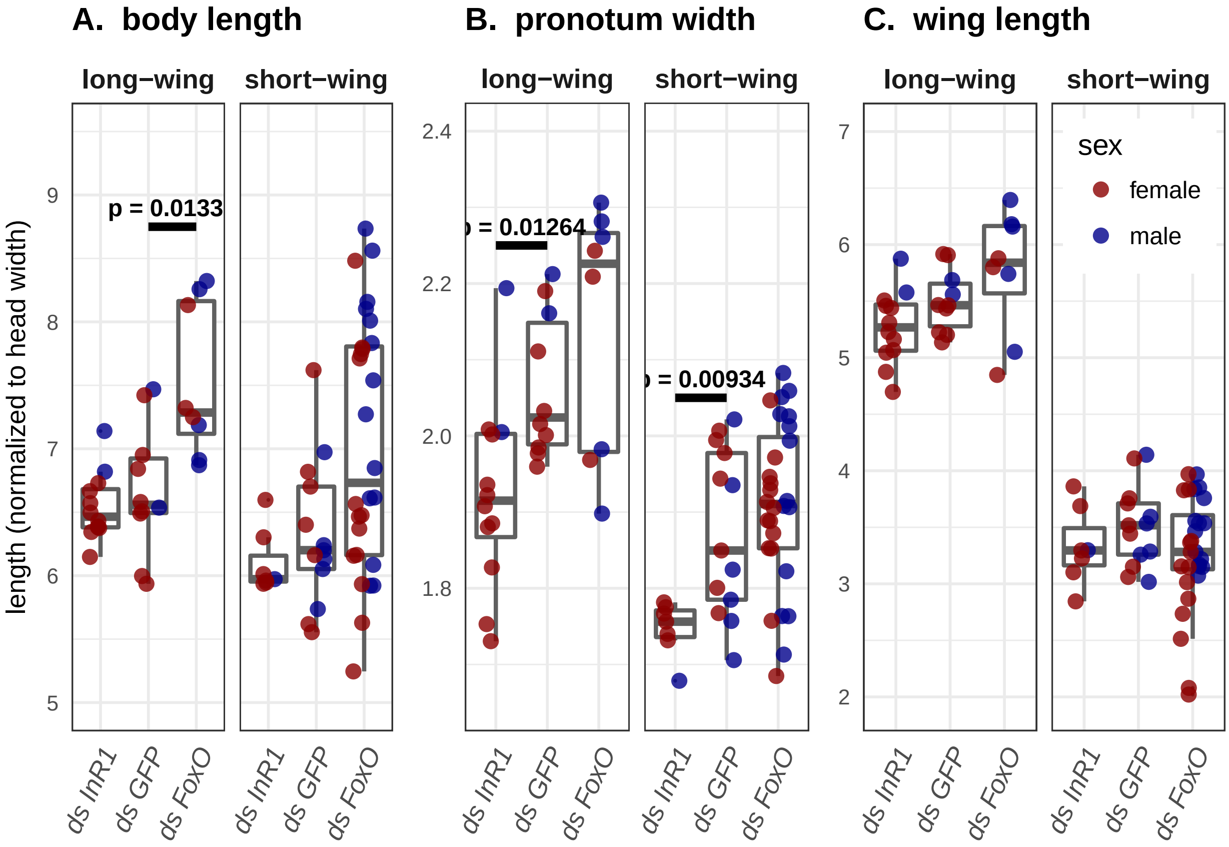

Supplementary Figure 12. Effects of RNAi on adult static allometries. Measurements were normalized by head width, anterior of the eyes. Dots represent individual samples. Tukey plots denote the median (black bar), interquartile range (white box) and full range of the data (whisker). (**a**) Body length is significantly greater for long-wing *FoxO* RNAi specimens, compared to nonspecific *GFP* dsRNA controls (Welch's t-test). (**b**) The width of the pronotum was significantly decreased in *InR1* RNAi compared to *GFP* controls in both morphs (Welch's t-test). (**c**) Wing length within each morph was not significantly different after *InR1* or *FoxO* dsRNA treatment.

Effects of *FoxO* RNAi on wing shape

```
cartesian.coords.rnai <- readland.tps.scale.option("Jhae.42LM.experimental.tps",
                                                    specID="ID", multiplyscale = FALSE)
```

```
##      Scale applied by division
## [1] "Specimen names extracted from line ID="
```

```
metadata.rnai <- read.csv("Jhae.42LM.experimental.tps.meta.csv", header=T)
```

We wished to examine whether *FoxO* RNAi produced changes in wing shape, as well as increases in the frequency of the short-wing morph. Therefore we placed landmarks on the wings of 77 *GFP* dsRNA-treated specimens and 64 *FoxO* RNAi specimens.

As with the unmanipulated data set, specimen landmarks were aligned using Generalized Procrustes Analysis with partial Procrustes superimposition and minimized bending energy (Supplementary Figure 13)[2].

```
wing.rnai.gpa <- gpagen(cartesian.coords.rnai[wing.lm,,],
                        curves=wing.semilandmarks,
                        ProcD=FALSE,
                        print.progress=FALSE)
# Remove centroid size for specimen 102, which had no dorsal scale included
wing.rnai.gpa$Csize[102] <- NA
plot(wing.rnai.gpa)
```

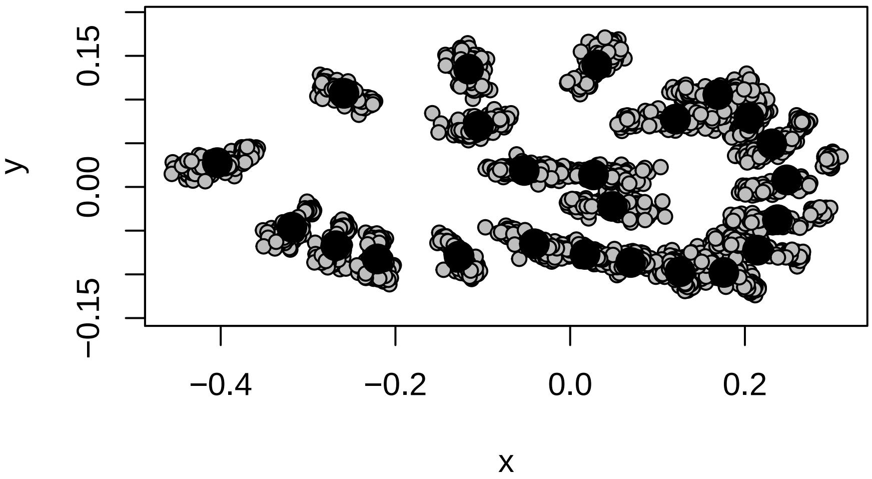

Supplementary Figure 13. Generalized Procrustes alignment of landmarks from 64 *FoxO* RNAi and 77 *GFP* dsRNA control specimens.

Variation in wing shape among RNAi specimens was examined using principal component projection in Kendall's tangent space.

```
rnai.gmm.colors <- c("FoxO"="#cc6600", "GFP"="#669900", "insulin"="#888888")
group.color <- rnai.gmm.colors
names(group.color) <- levels(metadata.rnai$dsRNA)
group.color <- group.color[match(metadata.rnai$dsRNA, names(group.color))]
wing.rnai.pca <- plotTangentSpace(wing.rnai.gpa$coords,
                                  warpgrids = T, groups = group.color, legend = T)
```

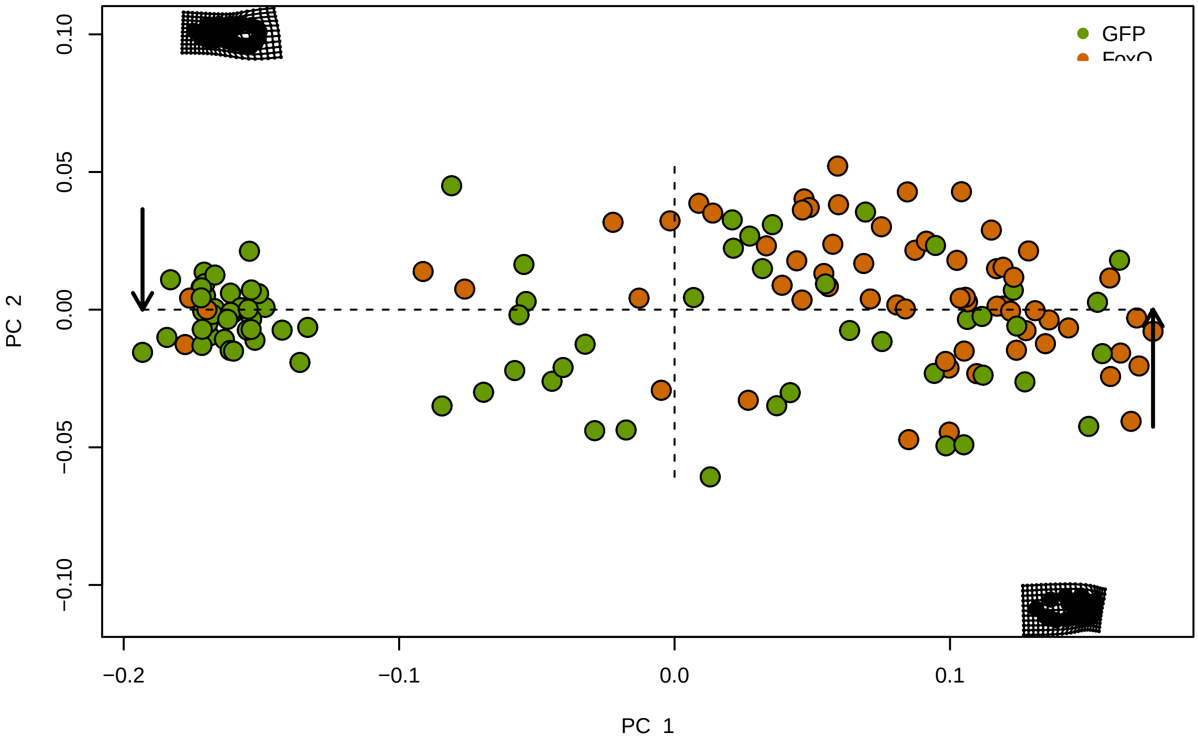

This plot was modified in Adobe *Illustrator* as Figure 6**a**. In that image, the x-axis is inverted to place “long” wing shapes to the right. n = 141.

As in the unmanipulated data set, the first PC axis distinguishes between short and long wing shapes. While *GFP* RNAi wings are evenly distributed in the short-wing morphospace, *FoxO* RNAi wings are clustered towards lower PC1 and higher PC2 values. This corresponds to a relative restriction of the posterior membrane and medial wing area, just proximal of the membrane, respectively (Figure 6b-e).

a. Consensus GFP dsRNA wing shape T103_28_GFP_SWm (Fig. 6e)

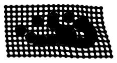

b. Consensus FoxO RNAi wing shape T006H_FoxO_SWm

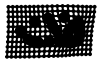

c. Extreme−PC1 GFP dsRNA wing shape T103_23_GFP_SWm (Fig. 6b)

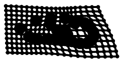

d. Extreme−PC1 FoxO RNAi wing shape T244_05_FoxO_SWf (Fig. 6c)

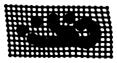

e. Extreme−PC2 GFP dsRNA wing shape T129_03_GFP_SWf

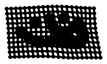

f. Extreme−PC2 FoxO RNAi wing shape T113_04_FoxO_SWm (Fig. 6d)

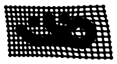

Supplementary Figure 14. Warp grids showing wing shapes from *GFP* and *FoxO* dsRNA treatments. Grid deformations are made in comparison to the mean shape for *GFP* dsRNA control short-wing specimens. (**a-b**) Consensus shapes for *GFP* and *FoxO* dsRNA treatments. (**c-d**) Extreme wing shapes from each treatment based on the first principal component axis. This selection corresponds to specimens on the far left of the morphospace in Figure 6**a**. (**e-f**) Extreme wing shapes from each treatment based on the second principal component axis. This selection corresponds to specimens at the top of the morphospace in Figure 6**a**. Photographs of some of the same specimens rendered here appear in Figure 6**b-e**, as noted.

Comparison of wing shapes for *GFP* and *FoxO* dsRNA treatments. Wing shapes from short-wing specimens in each treatment at the extremes of PC1 and PC2 were plotted as wrap grids, relative to the mean *GFP* treated short-wing shape.

Test of *FoxO* RNAi effects on wing shape

Procrustes ANOVA was used to test for the effect of *FoxO* RNAi on wing shape, while controlling for the influence of wing morph alone, which had previously been identified as a significant factor in wing shape.

```
i <- 9999 # number of iterations
wing.rnai.anova <- advanced.procD.lm(coords ~ morph + dsRNA, ~ morph,
                                     groups = ~ morph + dsRNA,
                                     data=gmm.metadata.rnai,
                                     iter=i, RRPP=TRUE, print.progress=F)
wing.rnai.anova$anova.table
```

```
##                     Df     SSE       SS       R2      F      Z Pr(>F)
## Y ~ morph          139 0.78185
## Y ~ morph + dsRNA 138 0.73567 0.046178 0.019819 8.6624 4.9483  1e-04 ***
## ---
## Signif. codes:  0 '***' 0.001 '**' 0.01 '*' 0.05 '.' 0.1 ' ' 1
```

```
# Print a symmetric matrix of pairwise p-values (no adjustment applied)
wing.rnai.anova$P.means.dist
```

```
##          LW:FoxO LW:GFP SW:FoxO SW:GFP
## LW:FoxO  1.0000 0.9093  0.2139 0.6579
## LW:GFP   0.9093 1.0000  0.0904 0.9806
## SW:FoxO  0.2139 0.0904  1.0000 0.0001
## SW:GFP   0.6579 0.9806  0.0001 1.0000
```

Controlling for the influence of morph on wing shape, *FoxO* RNAi had a significant influence on the shape of short wings (Procrustes ANOVA, $F_{138} = 8.6623522$, $p = 0.0001$; pairwise comparison of *GFP* v. *FoxO* dsRNA short-wing shapes $p = 0.0002$ with Bonferroni adjustment).

Session information

Total run time was 37 min for all analyses, completed on x86_64-apple-darwin15.6.0 (64-bit) running macOS Sierra 10.12.6.

Supplementary Note 2: List of associated files

Accompanying files necessary to repeat all analyses and render this document are available with the publication as a `zip` archive (Supplementary Data File 1). The contents of this file are listed below.

Filename	Description	Format
Fawcett.et.al.Supplementary.Notes.Rmd	Main R markdown code for generateion of this document	Rmd
Jhae.GMM.Rmd	Child R markdown file for geometric morphometric analysis	Rmd
Jhae.OA.Rmd	Child R markdown file for ontogenetic allometry	Rmd
Jhae.wild.Rmd	Child R markdown file for analysis of field data	Rmd
Jhae.modeling.Rmd	Child R markdown file for modeling of reaction norms	Rmd
Jhae.gene.exp.Rmd	Child R markdown file for gene expression analysis	Rmd
Jhae.RNAi.Rmd	Child R markdown file for analysis of RNAi results	Rmd
Jhae.GMM.RNAi.Rmd	Child R markdown file for GMM analysis of RNAi results	Rmd
Jhae.fecundity.Rmd	Child R markdown file for analysis of fecundity data	Rmd
FigS04.range.maps.png	Image file for Supplementary Figure 4	PNG
FigS07.InR.phylogeny.png	Image file for Supplementary Figure 7	PNG
FigS10.Dll.embryonic.RNAi.png	Image file for Supplementary Figure 10	PNG
FigS11.chico.Akt.RNAi.png	Image file for Supplementary Figure 11	PNG
Fawcett.et.al.bib	Bibliography for use by the R markdown file	BibTex
nature_no_etal.csl	Citation style information	CSL
cross.reared.csv	Results of cross-rearing experiments	CSV
Jhae.crosses.csv	Results of single-pair matings	CSV
Jhae.fecundity.csv	Female egg production by day	CSV
Jhae.oa.csv	Length measurements of individual juvenile bugs	CSV
Jhae.wild.ratios.csv	Information on wild populations	CSV
qPCR.data.csv	Gene expression data from unmanipulated bugs	CSV
qPCR.RNAi.data.csv	Gene expression data from RNAi specimens	CSV
reaction.norms.csv	Results for cohorts raised under different food conditions	CSV
RNAi.linear.csv	Length measurements of RNAi specimens	CSV
Jhae.testes.AG.csv	Size (area) measurements of testes and accessory glands	CSV
InR.MrB.tree.nex	InR amino acid alignment and MrBayes commands	Nexus
Jhae.42LM.tps	Landmark coordinate data for morphometric analyses	TPS
Jhae.42LM.tps.meta.csv	Metadata describing specimens in the TPS file	CSV
Jhae.42LM.experimental.tps	Landmark coordinate data from RNAi specimens	TPS
Jhae.42LM.experimental.tps.meta.csv	Metadata describing the RNAi specimens used for GMM	CSV
readland.tps.scale.option.R	R script with a modification of geomorph::readland.tps	text
logistic.regression.analysis.R	R script for logistic regression analysis	text
pairwise.perm.test.R	R script for pairwise permutation tests of multiple groups	text

Abbreviations: CSV, comma seperated values; CSL, citation style language; PNG, portable network graphics; Rmd, R markdown; TPS, thin-plate spline[4].

Supplementary Note 3: Required R Packages

R packages required to run the analysis are listed below with version numbers.

name	version
ape	5.0
arm	1.9-3
bindrcpp	0.2
car	2.1-6
dplyr	0.7.4
geomorph	3.0.5
ggplot2	2.2.1
knitr	1.19
lattice	0.20-35
lme4	1.1-15
lmPerm	2.1.0
magrittr	1.5
maps	3.2.0
MASS	7.3-48
Matrix	1.2-12
multcompView	0.1-7
perm	1.0-0.0
plyr	1.8.4
rgl	0.99.9
Rmisc	1.5

Supplementary References

1. Bookstein, F. L. *Morphometric tools for landmark data: geometry and biology.* **10,** 435 (1991).

2. Adams, D. C., Otárola-Castillo, E. & Paradis, E. geomorph: an R package for the collection and analysis of geometric morphometric shape data. *Methods in Ecology and Evolution* **4,** 393–399 (2013).

3. Abramoff, M. D., Magelhaes, P. J. & Ram, S. Image Processing with ImageJ. *Biophotonics* **11,** 36–42 (2004).

4. Rohlf, F. J. The tps series of software. *Hystrix* **26,** 9–12 (2015).

5. Adams, D. C. Evaluating modularity in morphometric data: challenges with the RV coefficient and a new test measure. *Methods in Ecology and Evolution* **7,** 565–572 (2016).

6. Huxley, J. S. *Problems of Relative Growth.* (Methuen, 1932).

7. Carroll, S. P. & Boyd, C. Host Race Radiation in the Soapberry Bug: Natural History with the History. *Evolution* **46,** 1053–1069 (1992).

8. Tsai, J.-F., Hsieh, Y.-X. & Rédei, D. The soapberry bug, Jadera haematoloma (Insecta, Hemiptera, Rhopalidae): First Asian record, with a review of bionomics. *ZooKeys* 1–41 (2013). doi:10.3897/zookeys.297.4695

9. Gilman, E. F. & Watson, D. G. *Fact Sheet ST-581 Sapindus drummondii Western Soapberry.* 1–3 (Environmental Horticulture Department, Florida Cooperative Extension Service, Institute of Food; Agricultural Sciences, University of Florida, 1994).

10. Gilman, E. F. & Watson, D. G. *Fact Sheet ST-338 Koelreuteria paniculata Goldenraintree.* 1–3 (Environmental Horticulture Department, Florida Cooperative Extension Service, Institute of Food; Agricultural Sciences, University of Florida, 1993).

11. Gilman, E. F. & Watson, D. G. *Fact Sheet ST-337 Koelreuteria elegans Flamegold.* 1–3 (Environmental Horticulture Department, Florida Cooperative Extension Service, Institute of Food; Agricultural Sciences, University of Florida, 1993).

12. Gilman, E. F. & Watson, D. G. *Fact Sheet ST-336 Koelreuteria bipinnata Chinese Flame-Tree.* 1–4 (Environmental Horticulture Department, Florida Cooperative Extension Service, Institute of Food; Agricultural Sciences, University of Florida, 1993).

13. iNaturalist.org. (2017). at <https://www.inaturalist.org/observations?taxon{_}id=127669>

14. Roff, D. A. The Evolution of Threshold Traits in Animals. *The Quarterly Review of Biology* **71,** 3–35 (1996).

15. Harrison, R. G. Dispersal Polymorphisms in Insects. *Annual Review of Ecology and Systematics* **11,** 95–118 (1980).

16. Dingle, H. & Winchell, R. Juvenile hormone as a mediator of plasticity in insect life histories. *Archives of Insect Biochemistry and Physiology* **35,** 359–373 (1997).

17. Akaike, H. A new look at the statistical model identification. *IEEE Transactions on Automatic Control* **19,** 716–723 (1974).

18. Hosmer, D. W. & Lemesbow, S. Goodness of fit tests for the multiple logistic regression model. *Communications in Statistics - Theory and Methods* **9,** 1043–1069 (1980).

19. Gelman, A., Goegebeur, Y., Tuerlinckx, F. & Van Mechelen, I. Diagnostic checks for discrete data regression models using posterior predictive simulations. *Journal of the Royal Statistical Society: Series C (Applied Statistics)* **49,** 247–268 (2000).

20. Carroll, S. P., Dingle, H. & Klassen, S. P. Genetic differentiation of fitness-associated traits among rapidly evolving populations of the soapberry bug. *Evolution* **51,** 1182–1188 (1997).

21. Levins, R. in *Monographs in population biology* (eds. Levin, S. A. & Horn, H. S.) 120 (Princeton University

Press, 1968).

22. Zera, A. J. & Denno, R. F. Physiology and Ecology of Dispersal Polymorphism in Insects. *Annual Review of Entomology* **42,** 207–230 (1997).

23. Bebas, P., Kotwica, J., Joachimiak, E. & Giebultowicz, J. M. Yolk protein is expressed in the insect testis and interacts with sperm. *BMC developmental biology* **8,** 64 (2008).

24. Fire, A., Xu, S., Montgomery, M. K., Kostas, S. A., Driver, S. E. & Mello, C. C. Potent and specific genetic interference by double-stranded RNA in Caenorhabditis elegans. *Nature* **391,** 806–811 (1998).

25. Hughes, C. L. & Kaufman, T. C. RNAi analysis of Deformed, proboscipedia and Sex combs reduced in the milkweed bug Oncopeltusfasciatus: novel roles for Hox genes in the Hemipteran head. *Development (Cambridge, England)* **127,** 3683–3694 (2000).

26. Angelini, D. & Kaufman, T. Functional analyses in the hemipteran Oncopeltus fasciatus reveal conserved and derived aspects of appendage patterning in insects. *Developmental Biology* **271,** (2004).

27. Koyama, T., Syropyatova, M. O. & Riddiford, L. M. Insulin/IGF signaling regulates the change in commitment in imaginal discs and primordia by overriding the effect of juvenile hormone. *Developmental Biology* **324,** 258–265 (2008).

28. Gu, S.-H., Lin, J.-L., Lin, P.-L. & Chen, C.-H. Insulin stimulates ecdysteroidogenesis by prothoracic glands in the silkworm, Bombyx mori. *Insect Biochemistry and Molecular Biology* **39,** 171–179 (2009).

29. Clancy, D. J., Gems, D., Harshman, L. G., Oldham, S., Stocker, H., Hafen, E., Leevers, S. J. & Partridge, L. Extension of life-span by loss of CHICO, a Drosophila insulin receptor substrate protein. *Science (New York, N.Y.)* **292,** 104–106 (2001).

30. Yamamoto, R. & Tatar, M. Insulin receptor substrate chico acts with the transcription factor FOXO to extend Drosophila lifespan. *Aging cell* **10,** 729–732 (2011).

31. Slade, J. D. & Staveley, B. E. Compensatory growth in novel Drosophila Akt1 mutants. *BMC research notes* **8,** 77 (2015).
