# Supplementary material for: Manipulation of insulin signaling phenocopies evolution of a host-associated polyphenism
Source: Nat Commun. 2018 Apr 27;9:1699. doi: 10.1038/s41467-018-04102-1 (PMC5923257; doi:10.1038/s41467-018-04102-1)
Supplement: Supplementary file 2 — Description of Additional Supplementary Files [file 41467_2018_4102_MOESM2_ESM.pdf]

Description of Additional Supplementary Files

File Name: Supplementary Data 1
Description: Supplementary Data 1 is a ZIP format archive file (4 Mb) containing the raw data, images, and scripts necessary to reproduce the analyses described in this study and render the Supplementary Information PDF file from R markdown.

The individual files compressed in Supplementary Data File 1 archive file are listed and described in Supplementary Note 2.
