# Supplementary figures and images for: Manipulation of insulin signaling phenocopies evolution of a host-associated polyphenism
Source: Nat Commun. 2018 Apr 27;9:1699. doi: 10.1038/s41467-018-04102-1 (PMC5923257; doi:10.1038/s41467-018-04102-1)

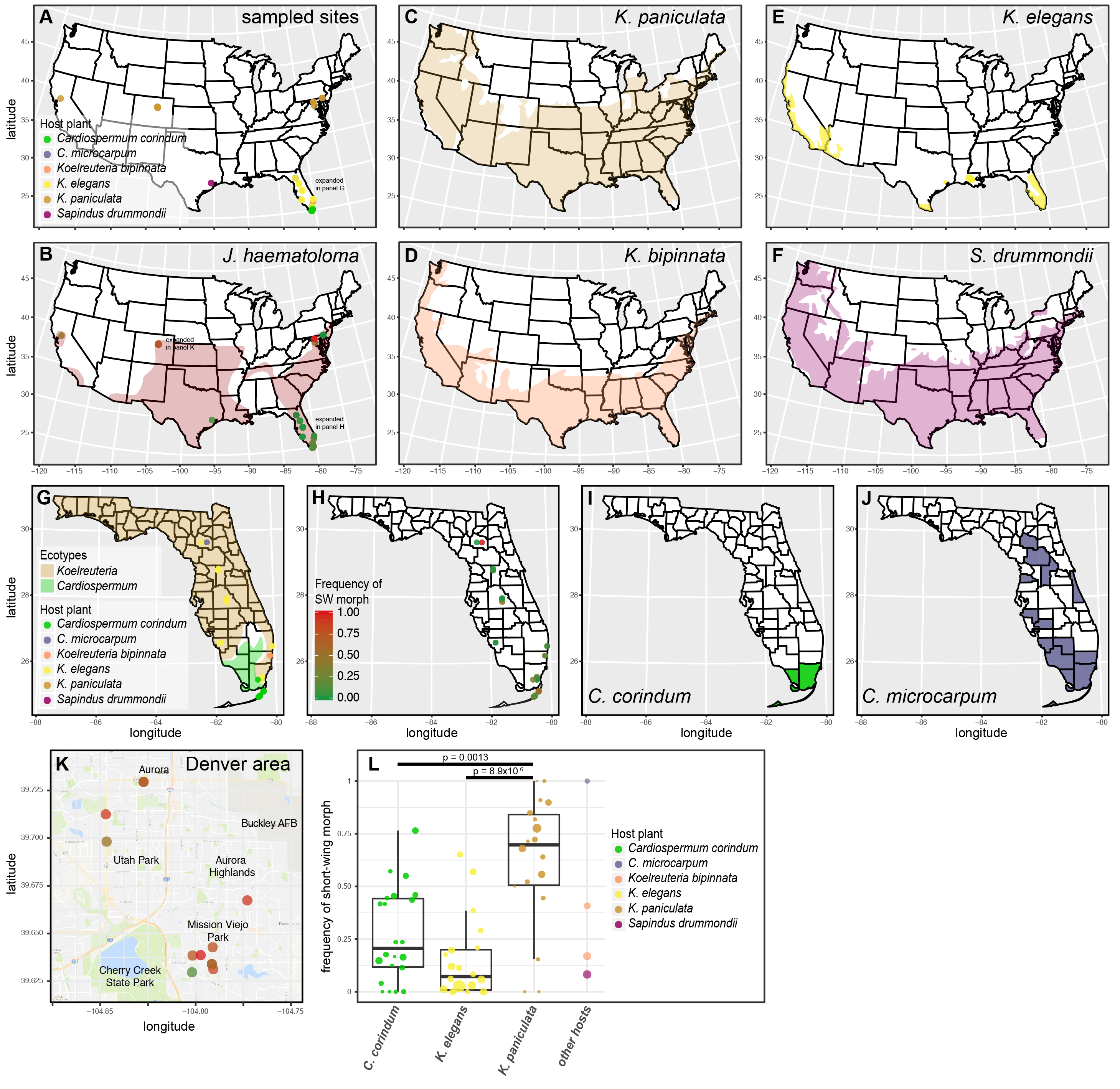

Supplement: Supplementary file 3 — Supplementary Data 1 [file 41467_2018_4102_MOESM3_ESM.zip › FigS04.range.maps.png]

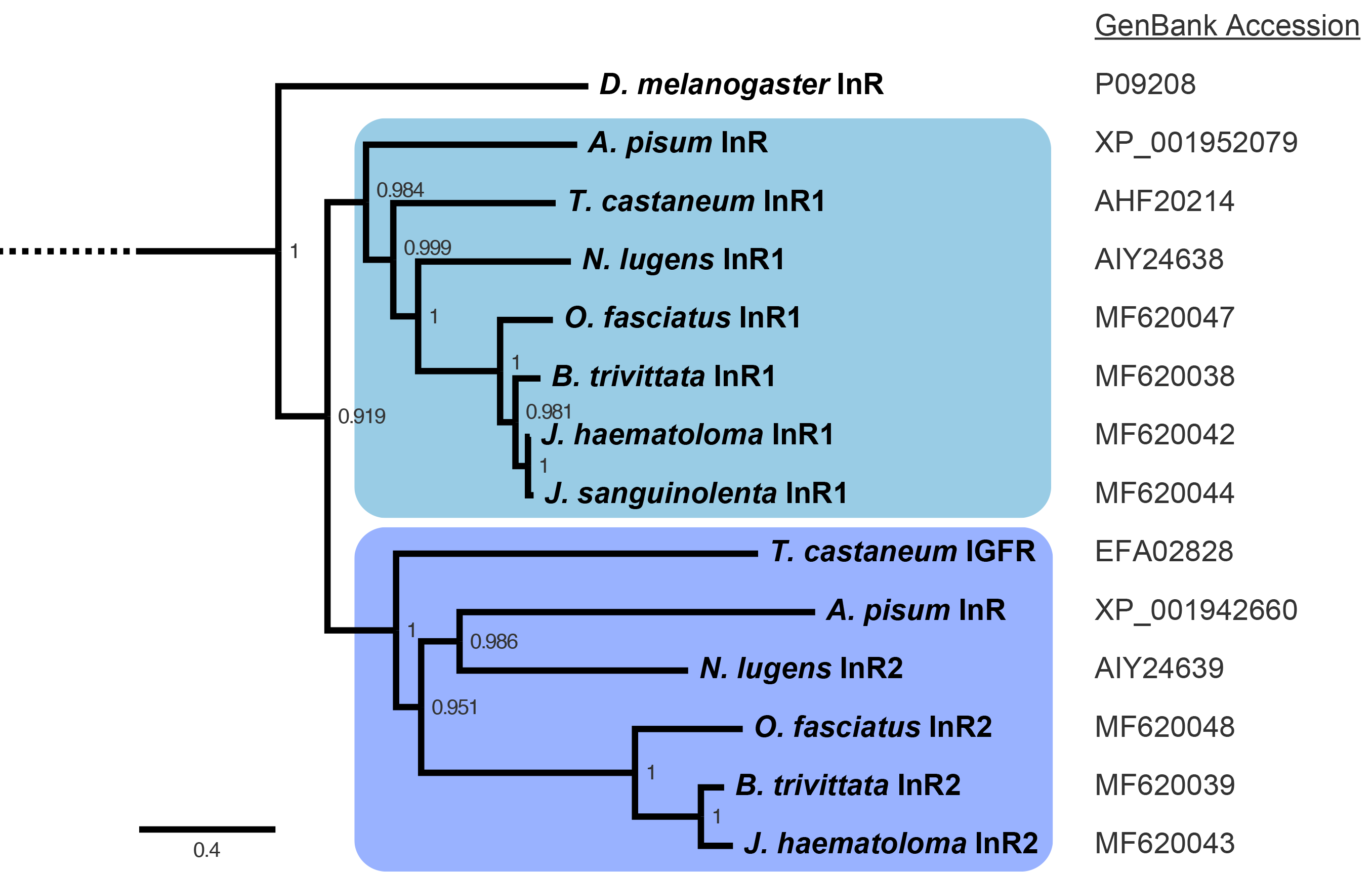

Supplement: Supplementary file 3 — Supplementary Data 1 [file 41467_2018_4102_MOESM3_ESM.zip › FigS07.InR.phylogeny.png]

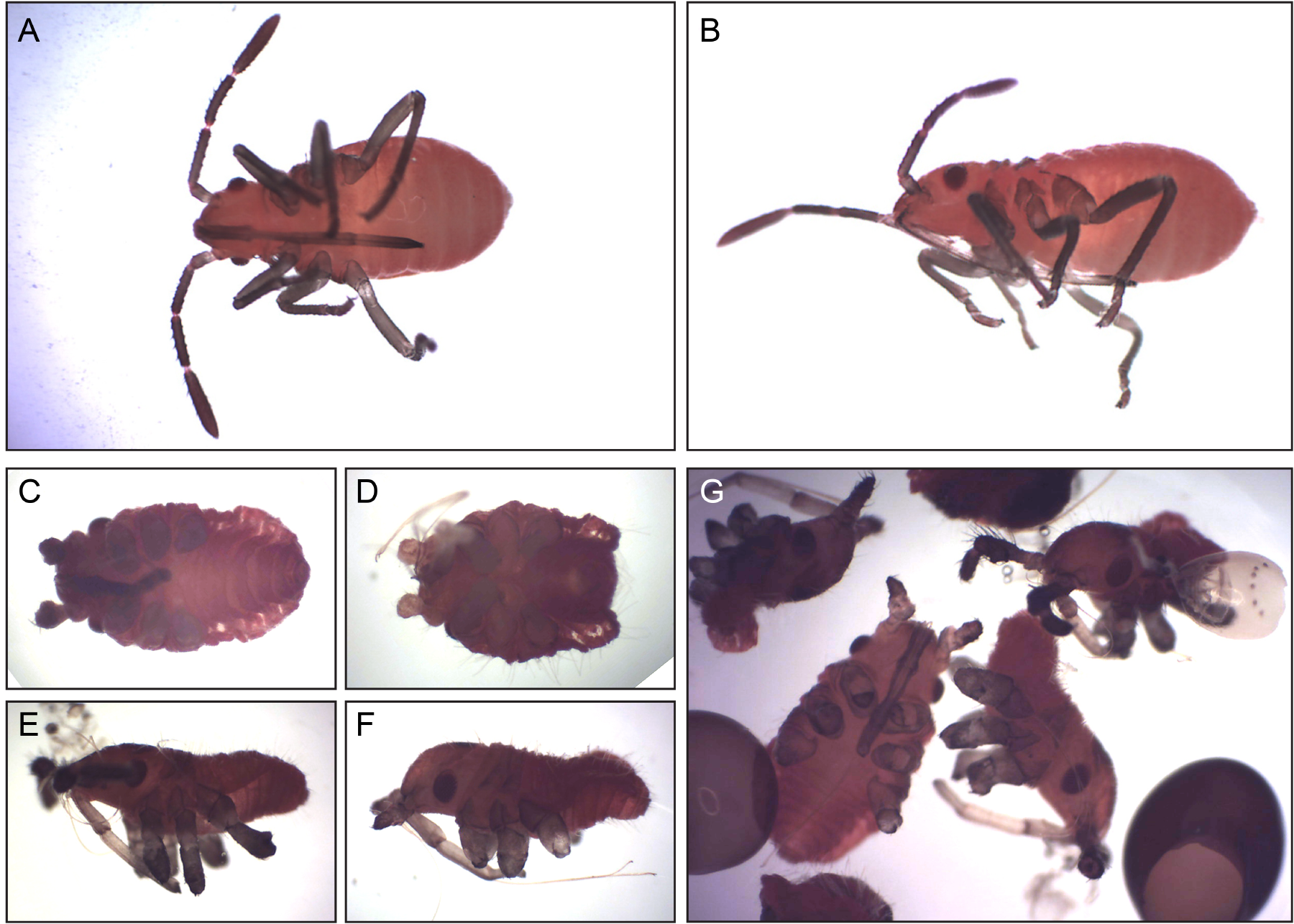

Supplement: Supplementary file 3 — Supplementary Data 1 [file 41467_2018_4102_MOESM3_ESM.zip › FigS10.Dll.embryonic.RNAi.png]

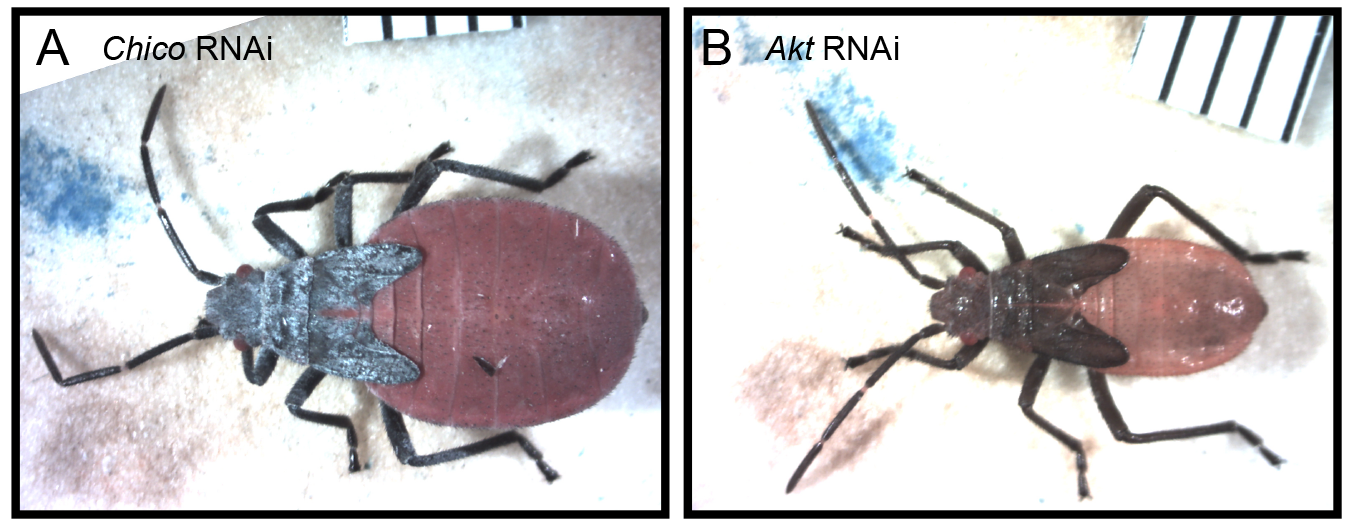

Supplement: Supplementary file 3 — Supplementary Data 1 [file 41467_2018_4102_MOESM3_ESM.zip › FigS11.chico.Akt.RNAi.png]
